# Continuous *n*-valerate formation from propionate and methanol in an anaerobic chain elongation open-culture bioreactor

**DOI:** 10.1186/s13068-019-1468-x

**Published:** 2019-05-27

**Authors:** Sanne M. de Smit, Kasper D. de Leeuw, Cees J. N. Buisman, David P. B. T. B. Strik

**Affiliations:** Environmental Technology, Wageningen University & Research, Axis-Z, Bornse Weilanden 9, 6708 WG Wageningen, The Netherlands

**Keywords:** Chain elongation, Selective pressure, Open-culture fermentation, Mixed-culture fermentation, Biobased chemicals, Methanol, Butyrate, *n*-Valerate

## Abstract

**Background:**

Chain elongation forms a new platform technology for the circular production of biobased chemicals from renewable carbon and energy sources. This study aimed to develop a continuous methanol-based chain elongation process for the open-culture production of a new-generation biofuel precursor and potential platform chemical: *n*-valerate. Propionate was used as a substrate for chain elongation to *n*-valerate in an anaerobic open-culture bioreactor. In addition, the co-production of *n*- and iso-butyrate in addition to *n*-valerate via, respectively, acetate and propionate elongation was investigated.

**Results:**

*n*-Valerate was produced during batch and continuous experiments with a pH in the range 5.5–5.8 and a hydraulic retention time of 95 h. Decreasing the pH from 5.8 to 5.5 caused an increase of the selectivity for *n*-valerate formation (from 58 up to 70 wt%) during methanol-based propionate elongation. *n*-Valerate and both *n*- and iso-butyrate were produced during simultaneous methanol-based elongation of propionate and acetate. Propionate was within the open-culture preferred over acetate as a substrate with 10–30% more consumption. Increasing the methanol concentration in the influent (from 250 to 400 mM) resulted in a higher productivity (from 45 to 58 mmol C/L/day), but a lower relative product selectivity (from 49 to 43 wt%) of *n*-valerate. The addition of acetate as a substrate did not change the average *n*-valerate productivities. Within the continuous bioreactor experiments, 6 to 17 wt% of formed products was methane. The microbial community during all steady-states in both methanol-based elongation bioreactors was dominated by species related to *Clostridium luticellarii* and *Candidatus Methanogranum*. *C. luticellarii* is the main candidate for *n*-valerate formation from methanol and propionate.

**Conclusions:**

*n*-Valerate was for the first time proven to be produced from propionate and methanol by an open-culture bioreactor. Methanogenic activity can be inhibited by decreasing the pH, and the *n*-valerate productivity can be improved by increasing the methanol concentration. The developed process can be integrated with various biorefinery processes from thermochemical, (bio)electrochemical, photovoltaic and microbial technologies. The findings from this study form a useful tool to steer the process of biological production of chemicals from biomass and other carbon and energy sources.

**Electronic supplementary material:**

The online version of this article (10.1186/s13068-019-1468-x) contains supplementary material, which is available to authorized users.

## Background

The growing world population causes arable land to become more scarce, waste to be produced in larger quantities and carbon emissions to rise due to fossil fuel usage [1, 2]. These developments emphasize the need for more sustainable and efficient production of chemicals. Microbial chain elongation processes can contribute to the realization of a more circular economy by providing a versatile approach to convert complex organic waste streams into fatty acids [short (C1–C5) and medium (C6–C10) chain length]. Currently, the company ChainCraft is starting a commercial demonstration factory using ethanol-based chain elongation to produce a mixture of carboxylate salts for using them as the feed additive in the agro-food industry [3].

Several chain elongation microbial pathways are known including homoacetogenesis (Wood–Ljungdahl pathway), the Arnon–Buchanan cycle and reverse beta-oxidation [4]. Chain elongation processes utilize short carbon chains as electron acceptor and by elongating the fatty acids, the carbon atoms are getting increasingly reduced (with a limit to 6 electrons per carbon). The products of the chain elongation conversions are largely dependent on the supplied substrates. Various electron donors can be used for chain elongation such as ethanol, methanol, lactate, sugars or electrons provided via microbial electrosynthesis [4–7]. Currently, the usage of methanol as an electron donor is not sufficiently investigated and therefore is the focus of this study.

Methanol is an available electron donor [8], which could be further mass produced via various thermochemical and electrochemical methods. Substrates for methanol production include lignocellulosic biomass, waste streams (e.g. via syngas) or CO_2_ sources including air [9–11]. Hypothetically, when methanol is used as an electron donor for chain elongation, a cobalamin-dependent methyltransferase system, coupled to the Wood–Ljungdahl pathway, could allow for its oxidation towards acetyl-CoA, which can then be utilized in a reverse beta-oxidation to reduce and elongate short chain fatty acids to longer carbon chains [12]. The short chain fatty acids acetate and propionate, which are substrates for the currently studied chain elongation process, can be produced via a hydrolysis or acidogenesis fermentation process. In addition, the acids can be produced from CO_2_ with pure or open-culture microbial electrosynthesis, from acetyl-CoA by biosynthesis and from amino acids [13–15].

The known products that can be formed within an open-culture methanol-based chain elongation of acidified supermarket waste are *n*-butyrate, iso-butyrate, *n*-valerate and *n*-caproate [16]. *n*-Valerate is a new-generation biofuel precursor and potential platform chemical. The esterification product of protonated *n*-valerate (valeric acid) can be used as an additive to diesel fuels [17, 18]; butyrate and *n*-valerate are also suitable precursors for bioplastic (polyhydroxyalkanoates) production [19] and Kolbe electrolysis of pure *n*-valerate would lead to gasoline (octane) formation [20].

*n*-Valerate production from waste streams is a rarely investigated environmental biorefinery process [21]. *n*-Valerate formation during chain elongation processes occurred in the previous research during methanol-based chain elongation from acidified supermarket waste. However, due to the presence of various electron donors (e.g. methanol and endogenous produced ethanol), it could not be shown whether methanol-based propionate elongation occurred [22]. A pure culture experiment with *Eubacterium limosum* also successfully showed propionate elongation with methanol to *n*-valerate [23, 24]. So far, no study focussed on utilizing an open culture to elongate propionate with methanol to *n*-valerate. Such open-culture microbiome could be advantageous for application since no sterilization is needed and a waste stream (i.e. organic waste) could be used as the feedstock during open-culture operation.

Fermentation of complex/mixed substrates using open-culture microbiomes typically results in a plethora of microbial processes that are either desired for chain elongation or are a competing process [25]. Reactor conditions such as temperature, pH, gas composition and hydraulic retention time (HRT) become important selective pressure tools that will determine the product spectrum [7, 22, 25]. In this study, pH and hydraulic retention time were varied to selectively inhibit competitive methanol consumption processes. Slow-growing methylotrophic methanogens and acetogens [26] are competitive methanol-consuming microbes and should be kept low in numbers by maintaining a suitably low HRT. This effect is enforced by operating at a low pH that causes more maintenance stress and overall lowers the growth rate of all microbes [27]. Moreover, the presence of undissociated acids at a low pH hinders bacteria due to futile cycling caused by diffusion of undissociated acids through the cell membranes [28–30]. Therefore, in combination with a low pH, high concentrations of volatile fatty acids could provide additional selection pressure, assuming that the microorganisms performing the desired chain elongation reactions are better suited to withstand this compared to undesired methanogenic and acetogenic microorganisms [31].

The aim of this study was to develop a continuous *n*-valerate production process using methanol-based chain elongation in an anaerobic open-culture reactor. Also, the co-production of *n*- and iso-butyrate in addition to *n*-valerate from methanol-based elongation using, respectively, acetate and propionate was investigated. Two continuous reactors were developed that successfully performed methanol-based chain elongation where propionate was elongated to *n*-valerate and acetate was elongated to *n*-butyrate and iso-butyrate.

## Results

### *n*-Valerate formation during batch experiments with pH ranging from 5 to 7.5

**Fig. 1:**
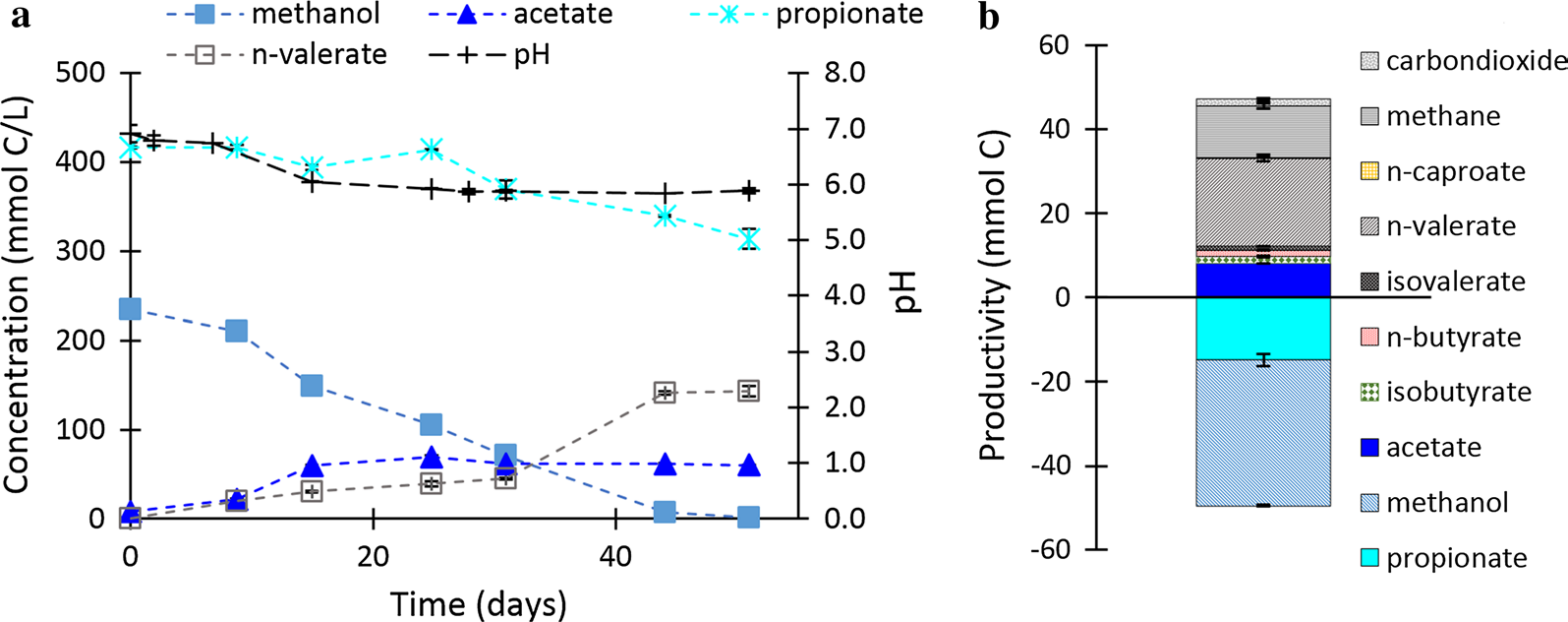
Concentration profile (**a**) during a batch experiment with methanol and propionate with the initial pH 7 at 308 K. Initially (day 0–25), acetate is formed via CO_2_ elongation with methanol concurrently with a pH drop. Propionate elongation to valerate starts slowly in the beginning; however, after 40 days when the pH is 5.8 it is the most prevalent metabolic activity. The total conversions at the end of the batch experiment are shown as well (**b**). The error bars represent the minimum and maximum values measured in the duplo experiments

The open-culture batch experiments showed that indeed methanol-based chain elongation of propionate is feasible. The series of batch experiments started at an initial pH ranging from 5 to 7.5 (with steps of 0.5) and showed propionate elongation with methanol to form *n*-valerate (*n*-C5). Figure 1 shows the results of a batch that started at pH 7. Additional file 1: Figure S2 shows the results of the methanol-based propionate elongation batches with initial pH values ranging from 5.5 to 7.5. Table 1 shows the main conversions that could occur during continuous methanol-based elongation of propionate and acetate.

**Table 1:** Overview of main catabolic conversions shown occurring during continuous methanol-based propionate and acetate elongation under anaerobic conditions at 308 K and pH 5.8 with the Gibbs free energy of the reactions

Nr	Description	Catabolic reaction	Δ*G*_r_^1^ (kJ/reaction)
1	Methylotrophic methanogenesis [32]	$4{\text{CH}}_{3} {\text{OH}} \to 3{\text{CH}}_{4} + {\text{HCO}}_{3}^{ - } + {\text{H}}_{2} {\text{O}} + {\text{H}}^{ + }$	− 310.8
2	Methylotrophic acetogenesis [33]	$4 {\text{CH}}_{3} {\text{OH}} + 2{\text{HCO}}_{3}^{ - } \to 3{\text{CH}}_{3} {\text{COO}}^{ - } + {\text{H}}^{ + } + 4{\text{H}}_{2} {\text{O}}$	− 178.7
3	*n*-Valerate formation from methanol and propionate	$2{\text{CH}}_{3} {\text{OH}} + {\text{C}}_{3} {\text{H}}_{5} {\text{O}}_{2}^{ - } \to {\text{C}}_{5} {\text{H}}_{9} {\text{O}}_{2}^{ - } + 2{\text{H}}_{2} {\text{O}}$	− 106.1
4	*n*-Butyrate formation from methanol and acetate [24]	$2{\text{CH}}_{3} {\text{OH}} + {\text{CH}}_{3} {\text{COO}}^{ - } \to {\text{CH}}_{3} \left( {{\text{CH}}_{2} } \right)_{2} {\text{COO}}^{ - } + 2{\text{H}}_{2} {\text{O}}$	− 106.1
5	Iso-butyrate formation from methanol and acetate	$2{\text{CH}}_{3} {\text{OH}} + {\text{CH}}_{3} {\text{COO}}^{ - } \to \left( {{\text{CH}}_{3} } \right)_{2} {\text{CHCOO}}^{ - } + 2{\text{H}}_{2} {\text{O}}$	− 106.1
6	Acetotrophic methanogenesis [34]	${\text{CH}}_{3} {\text{COO}}^{ - } + {\text{H}}_{2} {\text{O}} \to {\text{CH}}_{4} + {\text{HCO}}_{3}^{ - }$	− 49.9
7	Hydrogenogenic propionate degradation [35, 36]	${\text{CH}}_{3} {\text{CH}}_{2} {\text{COO}}^{ - } + 3{\text{H}}_{2} {\text{O}} \to {\text{CH}}_{3} {\text{COO}}^{ - } + {\text{HCO}}_{3}^{ - } + {\text{H}}^{ + } + 3{\text{H}}_{2}$	26.7 with pH_2_ 100 Pa
8	Hydrogenotrophic methanogenesis [37]	$4{\text{H}}_{2} + {\text{HCO}}_{3}^{ - } + {\text{H}}^{ + } \to {\text{CH}}_{4} + 3{\text{H}}_{2} {\text{O}}$	− 68.4 with pH_2_ 100 Pa
9	Hydrogenotrophic acetogenesis [38]	$4{\text{H}}_{2} + 2{\text{HCO}}_{3}^{ - } + {\text{H}}^{ + } \to {\text{CH}}_{3} {\text{COO}}^{ - } + 4{\text{H}}_{2} {\text{O}}$	− 36.1 with pH_2_ 100 Pa

The calculation of the Gibbs free energy is shown in Additional file 1 [67, 68]

### Continuous *n*-valerate formation with HRT 95 h and pH 5.5–5.8

**Fig. 2:**
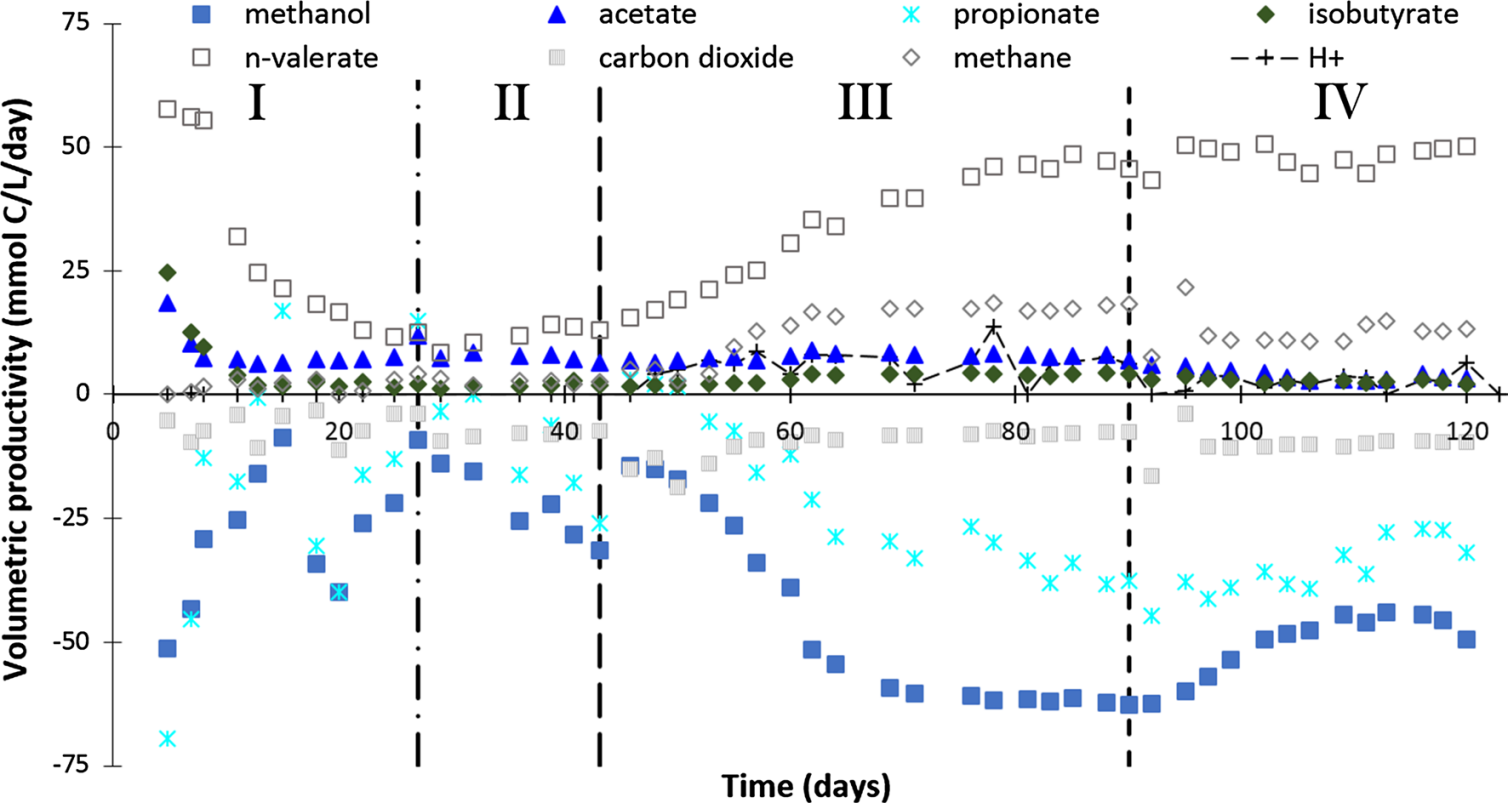
Volumetric productivities in time during continuous methanol-based propionate elongation in an anaerobic open-culture reactor at 309 K. The production of protons in mmol/day is also shown (  ). The vertical lines indicate the major changes in the setup: change of the hydraulic retention time (HRT) from 42 to 95 h (  ), pH change from 6.3 to 5.8 (  ) and pH change from 5.8 to 5.5 (  )

**Fig. 3:**
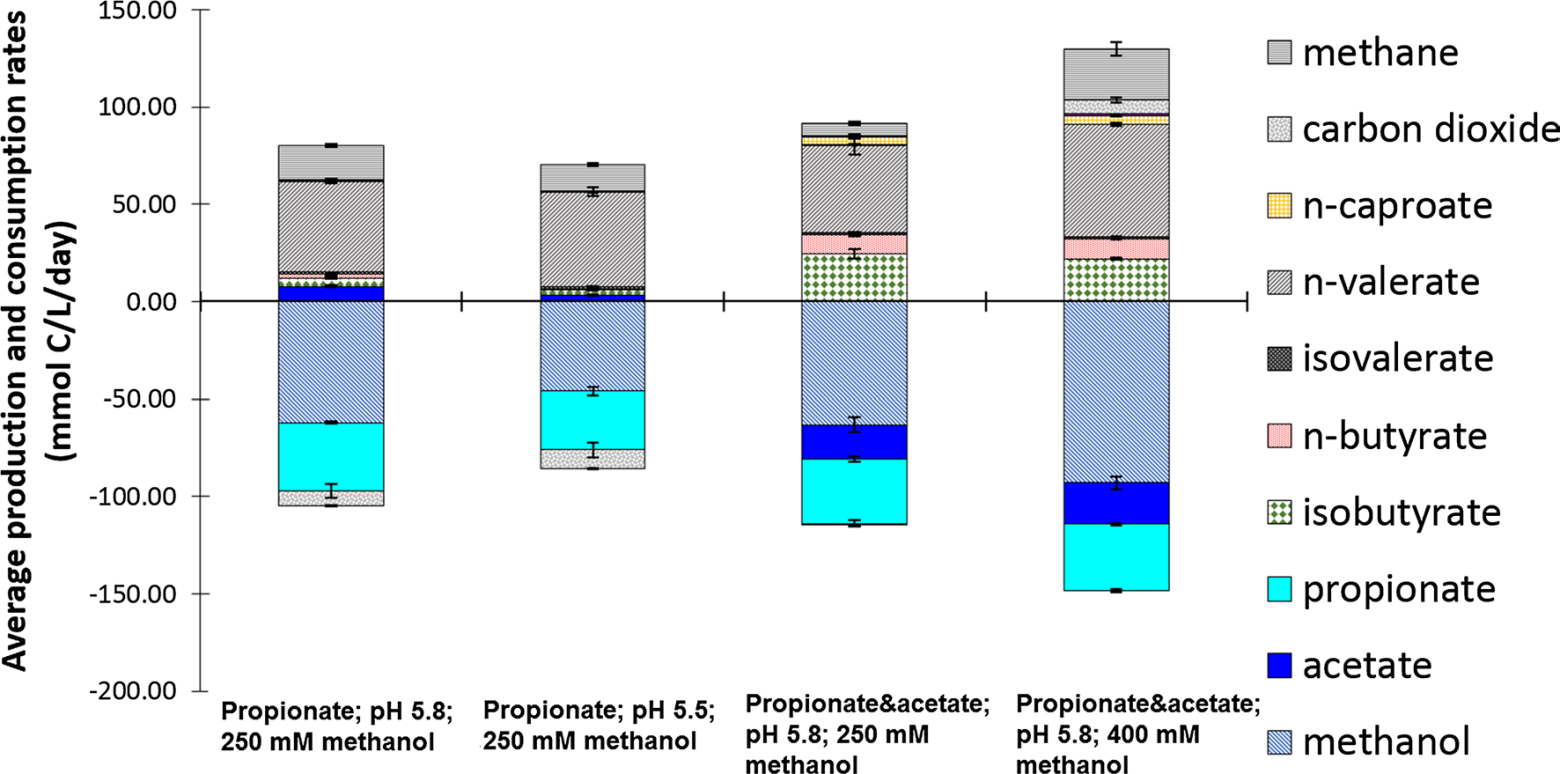
Average volumetric production and consumption rates during the steady-states of phase III (day 78–90) (pH 5.8) and phase IV (day 111–120) (pH 5.5) of continuous methanol-based propionate elongation in an anaerobic open-culture reactor and during the steady-state of phase V (day 64–71) (methanol in influent 250 mM) and during the last days of phase VI (day 97–104) (methanol in influent 400 mM) of continuous methanol-based propionate and acetate elongation in an anaerobic open-culture reactor at 309 K. The error bars represent the minimum and maximum values measured during the phase. Table 2 shows an overview of the average concentrations, productivities and relative product selectivities of *n*-valerate during the same four phases. The carbon balances for the four steady-states were 88 ± 2, 93 ± 4, 88 ± 4 and 92 ± 3% from left to right, the electron balances were 92 ± 2, 98 ± 4, 87 ± 4 and 91 ± 3%, respectively. The balances can be found in Additional file 1: Figure S3

*n*-Valerate was produced continuously during methanol-based propionate elongation by an anaerobic open-culture in a continuous reactor (Fig. 2). No methanol was consumed until the HRT was changed from 42 to 95 h (day 27, start of phase II). At the start of phase III (CO_2_ supply, day 43), the methanol concentration decreased rapidly and increased again after the pH was lowered to 5.5 (Additional file 1: Figure S3, day 90). The propionate consumption increased from the moment the pH was decreased to 5.8 (day 43). The *n*-valerate production was low (± 2.4 mmol/L/day) during phase II with pH 6.3 (day 27–43) and increased after the pH was decreased to 5.8 (day 43) to a value of 9.3 mmol/L/day. A slight increase in the *n*-valerate productivity (to 9.7 mmol/L/day) and concentration (40 mM) followed in phase IV, when the pH was 5.5 (day 111–120) (Fig. 3, Table 2). The concentration profiles of the most important compounds with the carbon and electron balances are shown in Additional file 1: Figure S3A.

**Table 2:** Average concentrations, volumetric productivities and relative product selectivities of *n*-valerate during the steady-states of phase III (day 78–90) (pH 5.8) and phase IV (day 111–120) (pH 5.5) of continuous methanol-based propionate elongation and during the steady-state of phase V (day 64–71) (methanol in influent 250 mM) and during the last days of phase VI (day 97–104) (methanol in influent 400 mM) of continuous methanol-based propionate and acetate elongation in an anaerobic open-culture reactor at 309 K

	Propionate; pH 5.8; 250 mM methanol	Propionate; pH 5.5; 250 mM methanol	Propionate and acetate; pH 5.8; 250 mM methanol	Propionate and acetate; pH 5.8; 400 mM methanol
Average *n*-valerate concentration (mM)	37.1 ± 0.9	38.4 ± 1.4	33.9 ± 2.3	42.8 ± 0.7
Average *n*-valerate productivity (mmol/L/day)	9.3 ± 0.2	9.7 ± 0.4	9.0 ± 1.0	11.5 ± 0.2
Average *n*-valerate selectivity (wt%)	58	70	49	43

### Formation of *n*- and iso-butyrate in addition to *n*-valerate during simultaneous elongation of acetate and propionate

The continuous experiment with simultaneous propionate and acetate elongation with methanol showed formation of both *n*- and iso-butyrate and *n*-valerate. The concentration profile of the most important compounds is shown in Additional file 1: Figure S3B. A steady-state was reached after 64 days; subsequently, the methanol concentration in the influent was increased (from 250 to 400 mM) to study whether a higher methanol concentration would lead to a higher chain elongation productivity. The concentrations of methanol in the reactor were 27 ± 4 mM and 76 ± 14 mM, respectively, during the steady-states with 250 mM methanol and 400 mM methanol in the reactor influent. After the increase of the methanol concentration in the influent from 250 to 400 mM, the *n*-valerate formation increased, whilst the productivities of iso-butyrate and *n*-butyrate stayed constant compared to the phase with 250 mM methanol in the influent (Fig. 3 and Additional file 1: Figure S4). More iso-butyrate was formed compared to *n*-butyrate; the iso-butyrate/*n*-butyrate ratios were 2.5 ± 0.3 and 2.1 ± 0.1, respectively, in the steady-states with 250 and 400 mM methanol in the influent of the reactor with continuous methanol-based propionate and acetate elongation (Fig. 3: right two bars). Small amounts (1 mM) of iso-valerate were observed during both continuous methanol-based chain elongation processes. This iso-valerate is likely produced from the amino acids in the yeast extract present in the medium [39, 40].

### Increase of relative methane selectivity after increase of methanol concentration

**Fig. 4:**
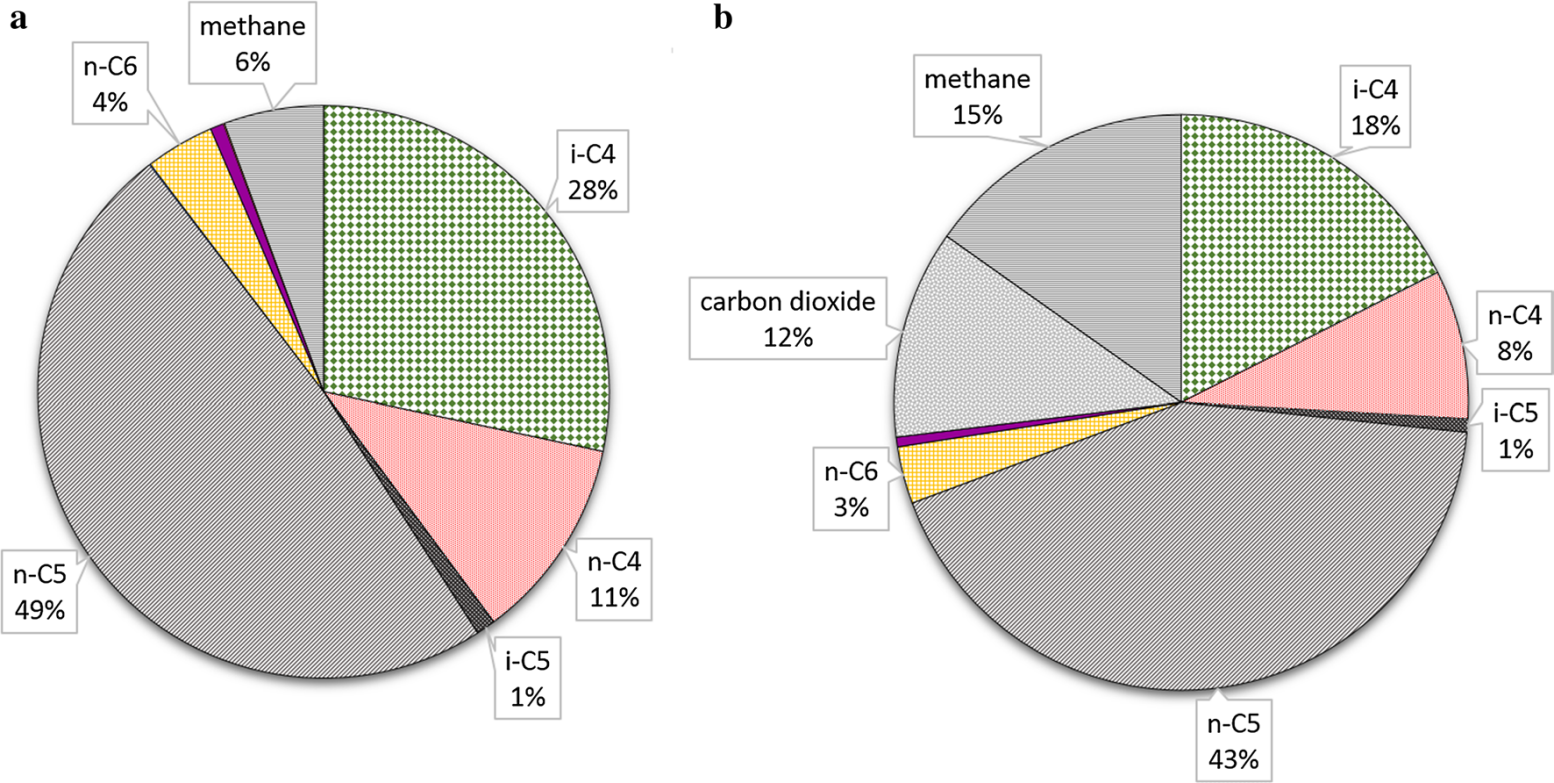
Relative selectivity of the formed *n*-butyrate (n-C4), iso-butyrate (i-C4), *n*-valerate (n-C5), iso-valerate (i-C5), *n*-caproate (n-C6), carbon dioxide (CO_2_) and methane (CH_4_) of continuous methanol-based propionate and acetate elongation in an anaerobic open-culture reactor at 36 °C at the steady-state with 250 mM methanol in the influent (**a**) (day 64–71) and at the last days with 400 mM methanol in the influent (**b**) (day 111–120). The values are calculated based on the production rates in g/L/day; the total production rates were 1.37 ± 0.26 (**a**) and 2.12 ± 0.18 (**b**) g/L/day

The increase of the methanol concentration in the reactor increased the *n*-valerate productivity, but decreased the relative selectivity for *n*-valerate production from 49 to 43 wt% of the total carbon containing products (Fig. 4, Table 2). The main reason for the decreased selectivity is the net carbon dioxide formation and the increased methane production that occurred with a higher methanol concentration in the influent.

### *Clostridium luticellarii* (species) mainly present during both acetate and propionate elongation

Within the methanol chain elongation reactor, two orders predominantly were present: *Clostridiales* and *Thermoplasmatales*. Table 3 shows the composition of the microbial communities at the end of the two last phases in both the continuous methanol-based propionate elongation reactor and the continuous methanol-based acetate and propionate elongation reactor. The most abundant OTU from the *Clostridiales* bacteria (~ 20%, ~ 17%, ~ 43% and ~ 17% of the total OTU count, respectively, for the phases from left to right in Table 3) appeared to be highly similar to *Clostridium luticellarii* (99.72% similarity, Additional file 1: Table S12). In Additional file 1: Tables S10–S13, more extensive information on genus level relative abundances, OTU counts and NCBI Megablast results can be found.

**Table 3:**
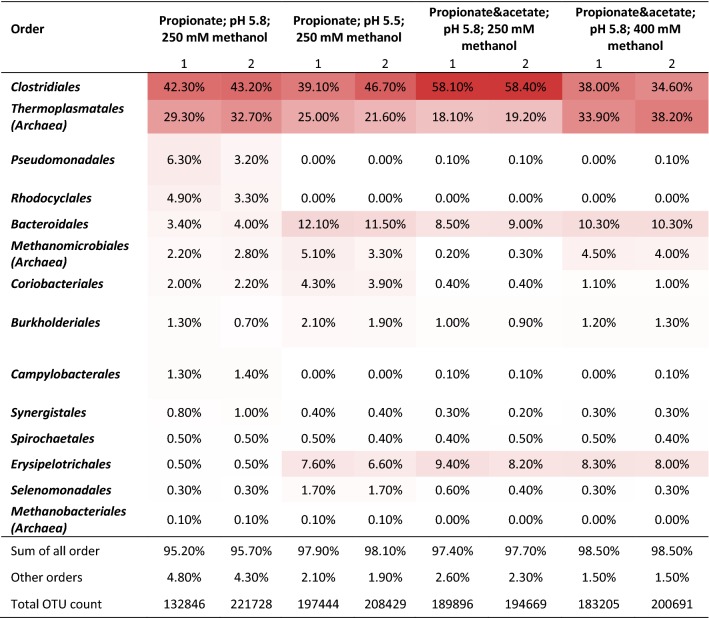
Overview of the relative abundances within the microbiomes, given in percentage at order level

The samples were taken at the end of the steady-states of phase III (day 90) (pH 5.8) and phase IV (day 125) (pH 5.5) of continuous methanol-based propionate elongation in an anaerobic open-culture reactor and at the end of the steady-state of phase V (day 69) (methanol in influent 250 mM) and during the last day of phase VI (day 106) (methanol in influent 400 mM) of continuous methanol-based propionate and acetate elongation in an anaerobic open-culture reactor at 309 K. For all biomass samples, the duplo results are shown indicated by 1 and 2. More detailed information and the relative abundances found for the inoculum samples can be found in Additional file 1: Tables S10 to S13

## Discussion

### *n*-Valerate formation during batch experiments with pH ranging from 5 to 7.5

During the batches, three predominant metabolic groups were deducted from the consumed substrates and produced biochemicals: (1) methylotrophic methanogens, (2) methylotrophic acetogens and (3) methanol-based propionate elongation (Table 1). Also traces of *n*-butyrate and iso-butyrate were found (Fig. 1b) which indicates methanol-based acetate elongation activity.

The degree in which the main metabolic activities occurred depended heavily on the initial pH and final concentrations. An initial pH of 7 and 7.5 caused some methanogenic activity and acetogenesis to occur in the beginning of the batch, whereas methanol-based chain elongation of propionate to *n*-valerate caught on after approximately 30 days when pH had already dropped to 5.8 ± 0.1. In the batch that started at pH 6.5, this pattern was similar but showed a longer lag phase and more extended/slowed production profile. The batches done at pH 6 and lower showed no significant production after 60 days (Additional file 1: Figure S2).

### Continuous *n*-valerate formation with HRT 95 h and pH 5.5–5.8

The hydraulic retention time (HRT) and the pH appeared to be critical for continuous *n*-valerate production. The HRT of 42 h appeared to be too short for the valerate producing organisms, since the concentration of *n*-valerate and the *n*-valerate productivity decreased exponentially after the startup of the experiment (Fig. 2: day 0–27). The *n*-valerate-producing culture probably washed out during the first phase, since a decrease in optical density was by eye observed. For this study, an HRT of 95 h was used, so most microorganisms that carry out competitive methanol-consuming processes (Table 1R1 and R2) could now theoretically grow in the continuous reactor, based on their growth rates found in the literature [26]. The minimum HRT will be between 42 and 95 h.

The chain elongation reactions occurred at pH values between 5.5 and 5.8 during both batch and continuous experiments. A pH of 5.5 is optimal for *n*-valerate formation during continuous methanol-based propionate elongation (Fig. 2). The pH decrease from 5.8 to 5.5 led to selectivity increase for *n*-valerate formation from methanol and propionate (from 58 to 70%, Table 2), which was attributed to two causes.

First, the acetate formation (Table 1R2) decreased after the pH was lowered from 5.8 to 5.5 (day 90) (Fig. 2). The theoretical available amount of dissolved carbon dioxide was maintained equal at pH 5.8 and 5.5, so substrate availability was not the reason for the decreased acetogenic activity (Additional file 1: Figure S1). The acetogenic activity could decrease at lower pH [41] or at higher concentration of undissociated acids present at the lower pH [28]. Less acetate was apparently available for *n*- and iso-butyrate formation at pH 5.5 (Table 1R5), so the *n*- and iso-butyrate formation decreased as well.

Second, the methanogenic productivity decreased after the pH was lowered from 5.8 to 5.5 (day 90) (Fig. 3). This finding is supported by the decreased relative abundance of the *Thermoplasmatales* family at pH 5.5 compared to pH 5.8 in the continuous methanol-based propionate elongation reactor (Table 3). The *Thermoplasmatales* family contains methane-producing archaea; they were identified to fall within uncultured species of the methylotrophic methanogen genus *Candidatus Methanogranum* [42] (Table 1R1). These archaea are known to utilize methanol as a substrate for methane formation, which well fits the observed methanogenesis in our system (Fig. 3) [43]. The methanogenesis inhibition with the pH decrease could, e.g., be caused by either the higher extracellular proton concentration or the higher concentration of undissociated volatile fatty acids [28–30].

### Increase of methanol concentration caused increase of both *n*-valerate productivity and methanogenesis

The *n*-valerate production improved after the increase of the methanol concentration in the influent, whilst the prior methanol concentration did not limit the *n*- and iso-butyrate production (Fig. 3). Methylotrophic methanogenesis (Table 1R1), one of the competing methanol-consuming processes, increased as a result of the high-methanol influent concentration in phase VI. The relative abundance of the *Candidatus Methanogranum* genus also increased with the increasing methanol concentration (Table 3), indicating a growth of the methanogenic bacteria community. The methanol concentration in the influent and reactor was the only parameter that changed between the two steady-states shown in Fig. 4, indicating that the methylotrophic methanogenesis was limited by the methanol concentration (27 ± 4 mM) during the first steady-state. Further, examination of the *K*_S_ value for methylotrophic methanogenesis under the described conditions can verify whether kinetics were indeed limiting at lower methanol concentrations. The yield of *n*-valerate over propionate was 1.01 mol/mol, whilst the yield of *n*- and iso-butyrate over acetate was 0.76 mol/mol during phase VI. The lower yield of butyrate over acetate indicates that acetate was consumed for other processes than butyrate formation; likely, acetate was utilized for biomass formation.

### *Clostridium luticellarii* (species) dominant candidate for continuous methanol-based propionate elongation

**Fig. 5:**
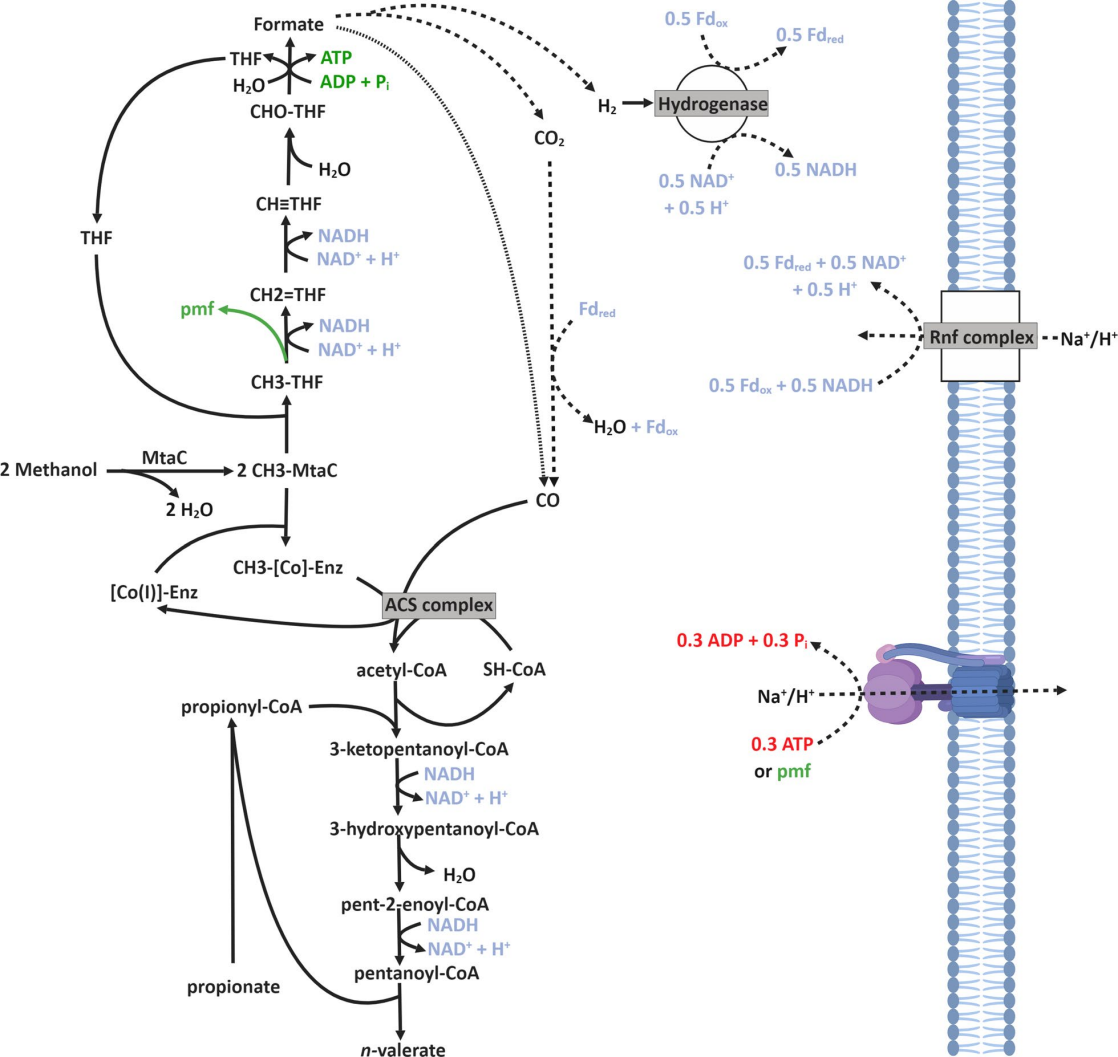
The hypothetically proposed mechanism for methanol-based propionate elongation to *n*-valerate [49]. Within the Wood–Ljungdahl pathway, one methanol is oxidized via the THF route to formate/CO, whilst another methanol is supplied to the ACS complex via a CH_3_–[Co]-enzyme intermediate [45, 46]. The ACS complex then catalyzes the formation of acetyl-CoA. Depending on the intracellular potential, formate could either be directly utilized for the formation of CO (dotted line) [46], or alternatively CO formation would require the bifurcating hydrogenase as well as an Rnf complex to balance the redox compounds (dashed line) [45]. The formed acetyl-CoA is then likely used in a thiolase-driven condensation step with propionyl-CoA to form 3-ketopentanoyl-CoA, similar to the reverse beta-oxidation mechanism in *C. kluyveri* [6]. The two NADHs generated during the oxidation of methanol are subsequently used to reduce 3-ketopentanoyl-CoA to 3-hydroxypentanoyl-CoA and to reduce pent-2-enoyl-CoA to pentanoyl-CoA. Because the methanol-based chain elongation of propionate to *n*-valerate (Table 1R3) has a Δ*G* of − 106.1 kJ/reaction, an ATP yield of 1.5 ATP would be expected (106.1 kJ/~ 70 kJ/ATP [50] = 1.5 ATP). This suggests that additional energy would be gained via a proton/Na^+^ motive force (pmf) that is likely generated at the oxidation of CH_3_-THF [50]. Potentially, additional bifurcation steps within the reverse beta-oxidation part might be necessary, depending on the intracellular redox potentials of the redox cofactors [50, 51]

*Clostridium luticellarii* is a known butyrate-producing strain (Additional file 1: Table S11) within the *Clostridium* sensu stricto 12 genus. It shares the highest similarities with *Clostridium ljungdahlii* and *Clostridium kluyveri* [44]. *C. luticellarii* is the prime candidate for performing the methanol-based chain elongation, which well fits its similarities to a Wood–Ljungdahl-harbouring (*C. ljungdahlii*) microorganism and a reverse β-oxidation-harbouring (*C. kluyverii*) microorganism. The OTU with a high similarity (100% cover, 99.72% identity, Additional file 1: Table S12) to *Clostridium luticellarii* has the highest relative abundance among the *Clostridium* sensu stricto 12 for both the methanol-based propionate elongation reactor and the methanol-based propionate and acetate elongation reactor. This finding suggests that *C*. *luticellarii* is responsible for both the elongation reactions from acetate to *n*- and iso-butyrate (Table 1R4 and R5) and the elongation from propionate to *n*-valerate (Table 1R3). The proposed mechanism by which *C. luticellarii* performs the methanol-based chain elongation reaction (for propionate elongation to *n*-valerate) is shown in Fig. 5. Methanol as electron donor is known to be metabolized within the Wood–Ljungdahl pathway where some methanol is reduced to CO, whilst the rest of the methanol is used for elongation with this CO to form acetyl-CoA [45, 46]. It was observed in this study that the electron acceptor (acetate and propionate) was always elongated with two-carbon units. This stoichiometry suggests that the elongation is executed via an acetyl-CoA thiolase-driven reaction similar to reverse beta-oxidation [47, 48]. The proposed route should be verified by isolating the responsible strain and by performing a genome analysis to identify the corresponding enzymes.

### Shift in catabolic conversions as a result of the pH change and the methanol increase

**Fig. 6:**
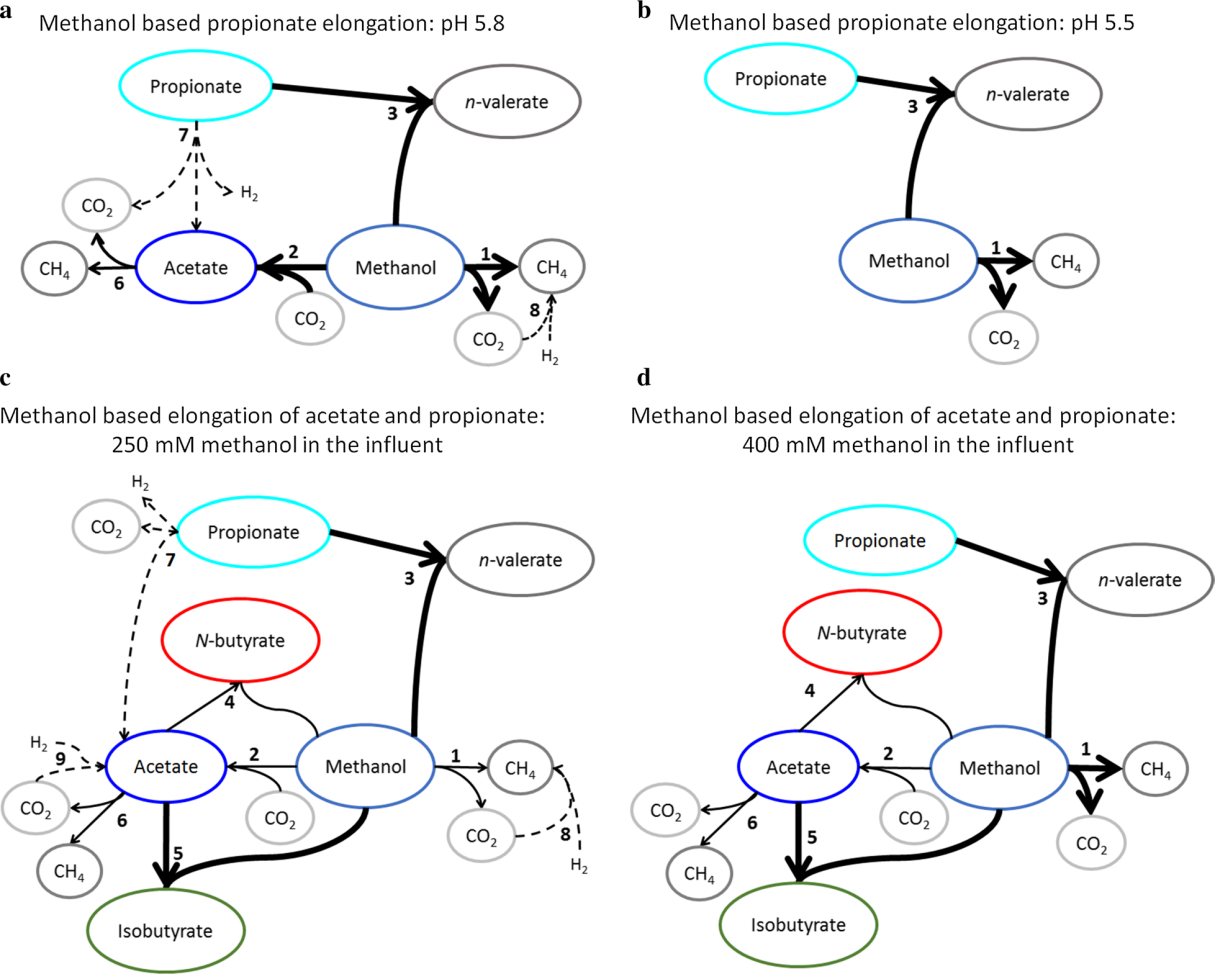
Schematic overview of the proposed main conversions that occurred during continuous methanol-based elongation of propionate (Pro) and simultaneous propionate and acetate (Pro&Ac) elongation at 309 K. The four scenarios represent the steady-states in the propionate elongation reactor at pH 5.8 (**a**) and pH 5.5 (**b**) and in the simultaneous propionate and acetate elongation reactor with 250 (**c**) and 400 (**d**) mM methanol in the reactor influent. The conversions with dashed arrows are proposed to maintain the electron balance. The main reactions are indicated with thicker arrows. For simplicity, only the productivities of the compounds with a value higher than 5 mmol/L/day are shown. The reaction equations and Gibbs free energy of the numbered conversions can be found in Additional file 1

During continuous methanol-based propionate elongation, the pH decrease from 5.8 to 5.5 caused a decrease in both acetate formation and *n*-valerate degradation (Fig. 6a, b). The microbial analysis that was performed during the steady-state at pH 5.8 was combined with the stoichiometric analysis to obtain an overview of the main catabolic conversions (Fig. 6a). The proton production decreased after the pH decrease (Additional file 1: Figure S3), suggesting methylotrophic acetogenesis (Table 1R2) is less likely to occur at pH 5.5.

The change in the molar yield of *n*-valerate over propionate (from 0.80 at pH 5.8 to 0.97 at pH 5.5) indicated that the degradation reaction of propionate (Table 1R7) did not occur during the steady-state at pH 5.5 (Fig. 6b). Only very small traces of H_2_ were observed during the continuous methanol-based elongation of acetate (max 0.02 mmol/L/day) and during the simultaneous elongation of acetate and propionate (max 0.03 mmol/L/day). Some blasted OTUs from the *Clostridium* sensu stricto 21 genus found during this study (Additional file 1: Table S11) were also found in mixed cultures where hydrogen production was observed [52, 53], so possibly propionate degradation by H_2_-producing bacteria took place at pH 5.8. However, the Gibbs free energy of the propionate degradation is positive at 100 Pa H_2_ (becomes < − 20 kJ/reaction at 0.1 Pa) making this reaction unlikely to have occurred. The conversion is dashed since it is unknown whether hydrogen produced during the propionate degradation. The competing methanol and propionate consumption processes (2 and 7) were almost completely inhibited at pH 5.5 (Fig. 6).

The increase of the methylotrophic methanogenesis conversion (Table 1R1) is the main change observed from the stoichiometric analysis of the steady-states in the reactor with simultaneous acetate and propionate elongation (Fig. 6c, d). The molar ratio between the productivity of methane and ${\text{CO}}_{2}$ is 3:1 (Fig. 3), supporting the proposed methylotrophic methanogenesis (Table 1R1). The molar yield of *n*-valerate per propionate increased (from 0.81 to 1.01) after the increased methanol concentration in the influent. This indicates that the propionate degradation reaction (Table 1R7) mainly occurred during the phase with 250 mM methanol in the influent. Since no significant net hydrogen production was measured in the reactor during the continuous acetate and propionate elongation process, a hydrogen-consuming process must have taken place if propionate was degraded to bicarbonate and hydrogen. Hydrogenotrophic methanogenesis (Table 1R8) and hydrogenotrophic acetogenesis (Table 1R9) are suggested to have occurred as hydrogen-consuming process during the steady-state with 250 mM methanol in the influent (Fig. 6c).

The molar yield of *n*- and iso-butyrate over acetate was 0.99 during the steady-state with 250 mM methanol in the influent, so when propionate degradation occurred, the hereby produced acetate (Table 1R7) has been consumed in another process. Acetotrophic methanogenesis (Table 1R6) is suggested to have occurred during the steady-state with 250 mM methanol in the influent (Fig. 6c). The molar yield of *n*- and iso-butyrate over acetate decreased (to 0.76) after the methanol concentration in the influent was increased, so presumably acetate was consumed in other processes than butyrate formation during the phase with 400 mM methanol in the influent (Fig. 6d).

### Outlook on application and process improvements

Microbially formed *n*-valerate can become an additional platform chemical for various applications. Instead of sugarcane molasses [54], more and possible cheaper substrates such as organic waste could be used as a substrate for *n*-valerate production. A recent study from Gonzalez-Garcia et al. [14] showed that propionate production can be achieved via various metabolic pathways. Microbially produced propionate will enter the market in the near future [14], which will make *n*-valerate production easier and more attractive. Mixtures of acetate and propionate can not only be produced from the earlier used supermarket/food waste [22], but are also reported for microbial electrosynthesis from CO_2_ [15, 55]. Methanol can be produced not only from syngas from lignocellulose or waste, but also by thermochemical CO_2_ conversions [9, 11, 56]. The electrosynthesis and thermochemical processes can be driven by electricity obtained from photovoltaics. In summary, with substrates from various waste sources and renewable energy, the developed process can be applied in the integrated and sustainable *n*-valerate biorefinery processes.

The developed *n*-valerate formation process can be further improved. In the experiments, the addition of acetate as a substrate did not lower the effectiveness of propionate elongation with methanol. Using a mixture of acetate and propionate for *n*-valerate production gave similar average *n*-valerate productivities (45.1 ± 4.8 mmol C/L/day with propionate and acetate, and 46.6 ± 1.1 mmol C/L/day with only propionate, at pH 5.8 and 250 mM methanol in the influent). Depending on the desired product spectrum, the decreased selectivity of the *n*-valerate production as a result of the addition of acetate as a substrate (from 58 to 43%), could form a disadvantage during *n*-valerate production with multiple substrates.

Two main findings can be used to increase the selectivity and productivity of methanol-based propionate elongation to *n*-valerate. First, the selectivity was improved by lowering the pH from 5.8 to 5.5 (within the propionate methanol elongation reactor) (Table 2). This increase in selectivity is mainly caused by the decrease of methylotrophic methanogenic activity. Second, the productivity was improved by increasing the methanol concentration in the influent from 250 to 400 mM (Table 2). The methanol concentration prior to increase was a limiting factor for the propionate chain elongation.

By combining these findings that allow for increased selectivity and productivity, the next step in optimizing process performance is to increase the biomass concentration. The use of carrier materials and biomass granulation are efficient strategies to improve the biomass concentration and retention, as shown during other studies [57–59]. Challenges then lie in maintaining the correct selective pressure, as decoupling solid retention time (SRT) from HRT might introduce new problems with methanogen retention in the system. Further optimizing additional factors like pH, volatile fatty acid concentrations, and CO_2_ availability in the bioreactor could provide methods to increase the selective pressure towards methanol chain elongation, by inhibiting methanogenic activity.

Chen et al. [22] used acidified supermarket waste as a substrate for methanol-based chain elongation at approximately pH 6.2 and a hydraulic retention time of 40 h. Based on the results from the current study, it can be suggested to decrease the pH (5.5–5.8) and increase the hydraulic retention time (to 95 h) to stimulate *n*-valerate from propionate-containing supermarket waste. In addition, the methane production could be decreased by decreasing the pH to 5.5. Implementing this knowledge about selective pressure can reduce the costs for *n*-valerate production and create a new open-culture process of biological production of chemicals from biomass and other carbon sources.

## Conclusions

*n*-Valerate was for the first time proven to be produced from propionate and methanol during a continuous methanol-based chain elongation process in an anaerobic open-culture reactor. Acetate formation from methanol occurred at pH 5.8 and decreased at pH 5.5 due to the higher proton concentration and/or the accompanying increased undissociated acids concentration. Methanol consumption did not occur at an HRT of 42 h, whilst an HRT of 95 h showed to be long enough for methanol-consuming chain elongation processes. The product selectivity for *n*-valerate was increased with pH lowering from 5.8 to 5.5 during continuous methanol-based propionate elongation. Propionate elongation to *n*-valerate and acetate elongation to iso-butyrate and *n*-butyrate occurred simultaneously in a continuous methanol-based chain elongation reactor with both propionate and acetate present as a chain elongation substrate. The addition of acetate as a substrate did not cause a decrease of the *n*-valerate productivity. The productivity of *n*-valerate was improved by increasing the methanol concentration in the influent from 250 to 400 mM during methanol-based elongation of propionate and acetate. *Clostridium luticellarii* was suggested to be most abundant during all the steady-states of methanol-based elongation of both propionate and simultaneous elongation of acetate and propionate and is therefore proposed as a main candidate for methanol-based chain elongation.

## Methods

Batch tests and continuous experiments were carried out during this study. A series of batch experiments was carried out in duplo with an initial pH ranging from 5 to 7.5 (with steps of 0.5) to study which pH range allowed propionate elongation to *n*-valerate. Serum bottles (250 mL) were filled with 150 mL medium with 250 mM methanol and 150 mM propionate and 10 ml inoculum from a continuous methanol-based acetate elongation reactor (De Leeuw K, unpublished) and placed in a shaking cabinet at 308 K. The initial headspace consisted of 20% CO_2_ and 80% N_2_. An elaborate overview of the medium composition can be found in Additional file 1.

### Continuous methanol-based chain elongation

**Fig. 7:**
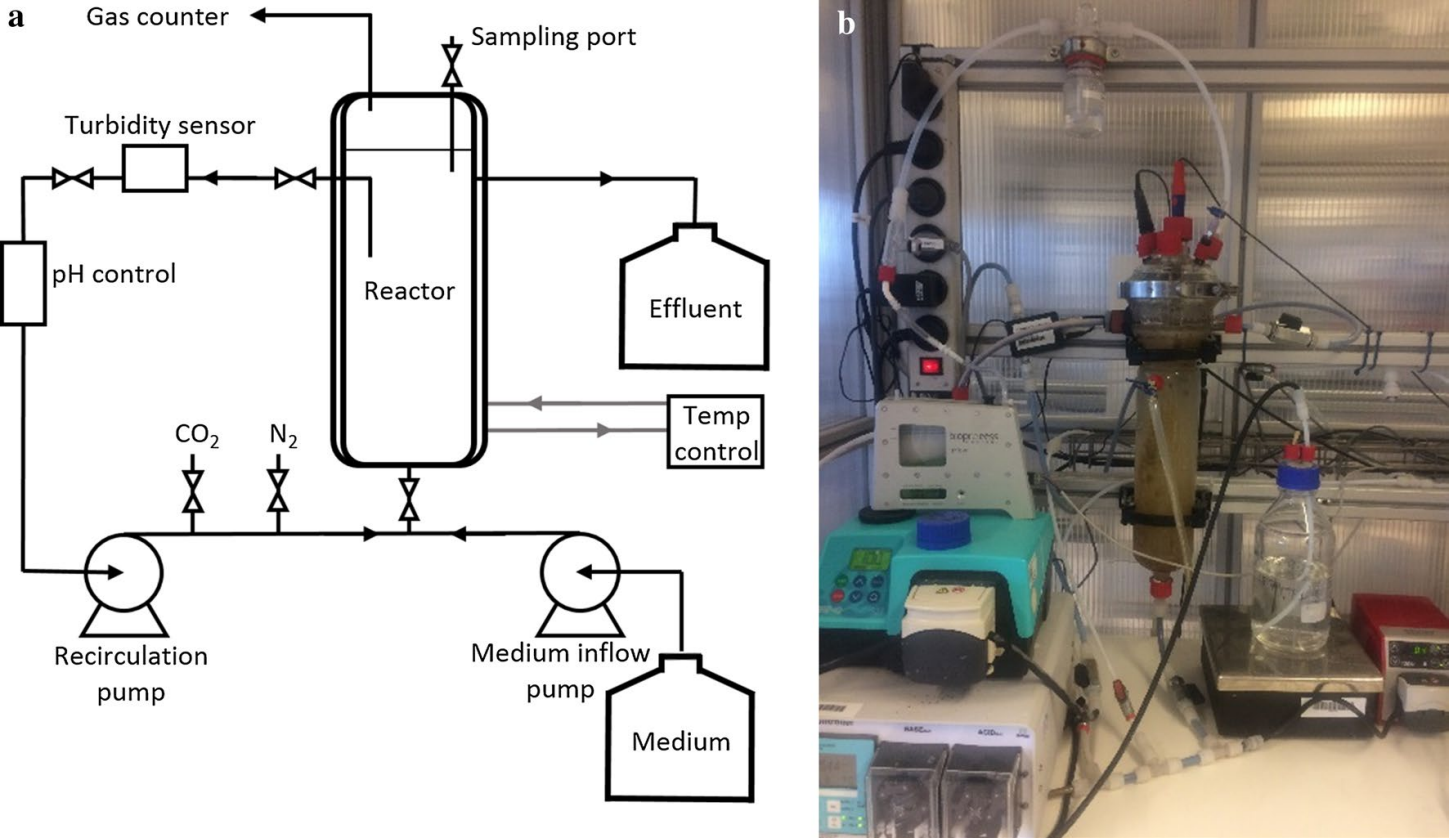
Schematic (**a**) and actual (**b**) setup of upflow anaerobic bioreactor used for continuous methanol-based chain elongation

A 1-L upflow anaerobic bioreactor (UAB) with 0.2-L headspace, as used by Chen et al. [16], was used for continuous methanol-based propionate elongation (Fig. 7). A gas counter was used to measure the amount of produced gas. The reactor content was recirculated with a peristaltic pump (Watson Marlow S CIQ 323, UK) with a velocity of 400 mL/min. In the recirculation loop, the turbidity and the pH (Endress Hauser M, Netherlands) were measured. The pH was maintained constant by automatic addition of potassium hydroxide.

The medium was pumped into the reactor by a peristaltic pump (Watson Marlow IP31, UK), the hydraulic retention time (HRT) could be adjusted by adjusting the pump rate. Carbon dioxide was supplied during the continuous methanol elongation process for acetate formation from methanol and bicarbonate (Table 1R2). The temperature of the reactor was kept at 309 K using a water bath (Julabo 4, Germany). A medium with 250 mM methanol, 150 mM sodium propionate and 1 g/L yeast extract was used. The exact composition of the medium is shown in Additional file 1: Table S1–S8. The medium was stored in a camelbag (MSR Dromedary, 6 L) in a fridge at 279 K that was continuously flushed with nitrogen.

A second reactor was used to study the simultaneous use of acetate and propionate as substrates for methanol-based chain elongation. A 3-L upflow anaerobic bioreactor (UAB) with 0.6-L headspace was used for continuous methanol-based acetate and propionate elongation (Fig. 7a). The recirculation velocity of the reactor content was 300 mL/min (Watson Marlow 505 S, UK); the turbidity and pH were measured in the recirculation loop (Endress Hauser M, Netherlands). The pH was maintained constant by automatic addition of potassium hydroxide.

The medium (250 mM methanol, 75 mM sodium propionate, 75 mM sodium acetate and 1 g/L yeast extract) was pumped into the reactor by a peristaltic pump (Watson Marlow IP31, UK). To compare the substrate use, propionate and acetate were added on a 1:1 molar base. The exact composition of the medium is shown in Additional file 1. The medium was stored in a camelbag in a fridge at 277 K that was continuously flushed with nitrogen. Carbon dioxide gas was let into the reactor continuously and the temperature of the reactor was kept at 309 K using a water bath (Julabo F25, Germany).

### Analysis

Three times a week, a gas sample was measured using gas chromatography to analyze the fractions of oxygen, carbon dioxide, methane, nitrogen and hydrogen in the gas phase (Shimadzu GC-2010, Japan). In addition, a liquid sample was taken, from which the concentrations of volatile fatty acids and alcohols (methanol, ethanol, propanol, butanol, pentanol, hexanol, acetate, propionate, *n*-butyrate, iso-butyrate, *n*-valerate, iso-valerate, *n*-caproate, iso-caproate, heptylate and caprylate) were measured using gas chromatography. A liquid sample was also taken from the fresh and old medium when the medium was replaced. The concentration of volatile fatty acids and alcohols of the medium samples was measured as well using gas chromatography (HP5890, USA). The difference between 2-methylbutyrate and 3-methylbutyrate could not be measured using the available equipment. Therefore, no distinction could be made between the formation of 2- and 3-methylbutyrate during this experiment and both compounds were measured as iso-valerate.

### Reactor setting changes

The setup changes during different phases of the continuous methanol-based propionate elongation process are shown in Table 4. The reactor was inoculated with biomass from the previously mentioned *n*-valerate formation batch experiments at day 0 and day 27. During phase I, decrease of the *n*-valerate formation was observed, the HRT was doubled for phase II. The pH during the *n*-valerate formation phase of the batch experiments was 5.8, so the pH was lowered to 5.8 to stimulate *n*-valerate formation even further in phase III. Steady-state was reached at day 78, and after 12 days of steady-state *n*-valerate formation, the pH of the medium was decreased to 5.5 to inhibit methanogenesis (phase IV). The carbon dioxide supply was changed with changing pH to maintain a constant bicarbonate concentration during the formation process. The change of the concentration of dissolved CO_2_ and the pH in time (Additional file 1: Figure S1) and the calculation of the required CO_2_ gas flow are shown in Additional file 1 [60, 61].

**Table 4:** Setup for the different phases of the continuous formation of valerate from propionate and methanol to study the effect of pH lowering on *n*-valerate formation

Phase conditions	Phase I	Phase II	Phase III	Phase IV
Hydraulic retention time (h)	42.3	*95.2*	95.2	95.2
Phase duration (days)	0–27	27–43	43–91	91–120
pH influent	7.0	7.0	*5.8*	*5.5*
pH reactor	6.4 ± 0.3	6.3 ± 0.2	*5.8*	*5.5*
CO_2_ supply (mL/min)	0.18	0.18	*0.36*	*0.40*

The changed parameters are indicated in italic

The pH in the reactor was controlled during phase III and phase IV

The setup changes during different phases of the continuous methanol-based acetate and propionate elongation process are shown in Table 5. The reactor was inoculated with biomass from the *n*-valerate formation batch experiments at day 0. No conversions of methanol and acetate were observed during phase I, so the pH was lowered from 7.0 to 5.8. Carbon dioxide was supplied to the reactor from phase III and onwards. During phase III, the high methanol concentration in the reactor was presumed to be prohibiting microbial activity. Therefore, the reactor was set in batch mode during phase IV, so that the methanol could be consumed and biomass could be accumulated. The hydraulic retention time was increased after the batch phase (during phase V) to prevent washout of *n*-valerate-producing organisms. A steady-state was reached from day 64 to day 71. In phase VI, the methanol concentration was increased to study whether increase of the concentration of methanol would stimulate the formation of *n*- and iso-butyrate and *n*-valerate at the same biomass retention rate.

**Table 5:** Setup for the different phases of the continuous formation of valerate and *n*- and iso-butyrate from propionate, acetate and methanol to study the effect of methanol increase on *n*- and iso-butyrate and *n*-valerate formation

Phase conditions	Phase I	Phase II	Phase III	Phase IV	Phase V	Phase VI
Hydraulic retention time (h)	46.3	42.5	45.4	**∞**	*90.5*	87.8
Phase duration (days)	0–17	17–20	20–38	38–45	45–71	71–104
pH influent	7.0	*5.8*	5.8	5.8	5.8	5.8
pH reactor	6.9 ± 0.1	*6.1 ± 0.2*	5.8 ± 0.1	5.8 ± 0.0	5.7 ± 0.1	5.8 ± 0.1
CO_2_ supply (mL/min)	0	0	*0.18*	*0.36*	0.36	0.36
Methanol in influent (mM)	250	250	250	**–**	250	*400*

The changed parameters are indicated in italic

The pH in the reactor was controlled during phase II to phase VI. The methanol concentration of the influent was increased from 250 mM to 400 mM in phase VI. The reactor was in batch mode during phase IV

At day 29 (during phase III) and 38 (start of phase IV), the reactor was inoculated with a mix of biomass from the methanol elongation reactor with propionate and a continuous methanol elongation reactor with acetate. The carbon dioxide supply was changed with changing pH to maintain, a similar theoretically supplied bicarbonate concentration constant during the formation process from phase IV (Additional file 1: Figure S1).

### Stoichiometric analysis

A stoichiometric analysis of the occurred conversions was performed based on the main possible conversions shown in Table 1, combined with the observed changes in the compound composition of the reactors and the obtained carbon and electron balances. The carbon balances per data point are shown in Additional file 1: Figure S3. Further indications of the conversions were given by the measured production of protons and gases (methane, carbon dioxide, hydrogen).

### Microbial community analysis

To characterize the enrichment of the biomass during propionate methanol chain elongation, samples of both inoculum and steady-state biomass were used for 16s rDNA analyses. Inoculum biomass was taken 3 months after taking the last sample from one of the batches that was started at pH 6.5. Reactor biomass was taken from the propionate methanol chain elongation reactor at day 90 (last day of steady-state), before the next phase was started. Both biomass samples were taken, in duplo, by spinning down 20 mL in a centrifuge tube and snap-freezing the pellet using liquid hydrogen. From here on, the in duplo taken samples were analyzed separately.

DNA was extracted from the pellets using the Powersoil DNA isolation kit according to their instruction manual. The isolated DNA was then used as the template to amplify the V3–V4 regions of 16s rDNA via PCR using the primer sets provided by Takahashi et al. [62]. This allowed simultaneous amplification of bacterial and archaean 16s rDNA. The illumina library generation [62] methods were subsequently used to generate DNA sequence data.

After acquiring rDNA sequence data, a statistical analysis allowed OTU picking, using the SILVA version 128 16S reference database and uclust [63, 64]. The RDP classifier (version 2.2) [60] was trained with the same SILVA reference database and subsequently used to classify the OTUs. Taxonomic analysis was performed using QIIME software version 1.9.1 [61]. This bioinformatics process was performed on 21 August 2018. From the acquired data, a heat map such as shown in Additional file 1: Table S10 could be made using Microsoft Excel. Open-source software Rstudio v3.5.0 was used to sort the data and create quantitative OTU tables (as in Additional file 1) that belonged to a chosen taxonomic group. This allowed counting the most abundant OTU’s that were classified within a single genus. The rDNA sequences of selected abundant OTUs (as in Additional file 1: Table S12) were then used for Megablast to search within the NCBI nucleotide database on 18 April 2019.

## Additional file

**Additional file 1.** Supplementary information–Continuous *n*-valerate formation from propionate and methanol in an anaerobic chain elongation open-culture bioreactor.

## Data Availability

All data generated or analyzed during this study are included in this published article (and its Additional file). Microbiota raw sequencing data are submitted to the ENA database (https://www.ebi.ac.uk/ena) under accession number PRJEB32281. Raw experimental data is available in the DANS-EASY database (10.17026/dans-zez-ykrc).
