# Supplementary material for: Continuous n-valerate formation from propionate and methanol in an anaerobic chain elongation open-culture bioreactor
Source: Biotechnol Biofuels. 2019 May 27;12:132. doi: 10.1186/s13068-019-1468-x (PMC6535856; doi:10.1186/s13068-019-1468-x)
Supplement: Supplementary file 1 — Additional file 1. Supplementary information–Continuous n-valerate formation from propionate and methanol in an anaerobic chain elongation open-culture bioreactor. [file 13068_2019_1468_MOESM1_ESM.docx]

Supplementary Information

Continuous n-valerate formation from propionate and methanol in an anaerobic chain elongation open-culture bioreactor

Sanne M. de Smit†, Kasper D. de Leeuw†, Cees J. N. Buisman and David P. B. T. B. Strik*

*Correspondence: david.strik@wur.nl

†Sanne M. de Smit and Kasper D. de Leeuw contributed equally to thiswork

Environmental Technology, Wageningen University & Research, Axis-Z,

Bornse Weilanden 9, 6708 WG Wageningen, The Netherlands

Keywords: Chain elongation, Selective pressure, Open-culture fermentation, Mixed-culture fermentation, Biobased

chemicals, Methanol, Butyrate, n-Valerate

The supplementary information section contains 36 pages, with 4 figures and 13 tables.

## Medium composition for batch methanol based elongation with propionate

| **Table S1.** Medium composition for the batch methanol elongation reactor with propionate as chain elongation substrate. The compositions of stock I, stock II, vitamin B solution, trace element 1 and trace element 2 solution are shown in **Table S4** to **Table S8**. | | |
| --- | --- | --- |
| **Compound** | **Amount per L** | **Unit** |
| Sodium propionate | 14.4 | g |
| Yeast Extract | 1.00 | g |
| Stock I | 20 | ml |
| Stock II | 20 | ml |
| Methanol (liquid) | 8.0 | g |
| Vitamin B solution | 1 | ml |
| Trace element 1 | 0.5 | ml |
| Trace element 2 | 0.5 | ml |

## Medium composition for continuous methanol based elongation with propionate

| **Table S2.** Medium composition for the continuous methanol elongation reactor with propionate as chain elongation substrate. The compositions of stock I, stock II, vitamin B solution, trace element 1 and trace element 2 solution are shown in **Table S4** to **Table S8**. | | |
| --- | --- | --- |
| **Compound** | **Amount per L** | **Unit** |
| Sodium propionate | 14.4 | g |
| Yeast Extract | 1.00 | g |
| Stock I | 20 | ml |
| Stock II | 20 | ml |
| Methanol (liquid) | 8.0 | g |
| Vitamin B solution | 1 | ml |
| Trace element 1 | 0.5 | ml |
| Trace element 2 | 0.5 | ml |

## Medium composition for continuous methanol based elongation with propionate and acetate

| **Table S3.** Medium composition for the continuous methanol elongation reactor with propionate and acetate as chain elongation substrates. The compositions of stock I, stock II, vitamin B solution, trace element 1 and trace element 2 solution are shown in **Table S4** to **Table S8**. | | |
| --- | --- | --- |
| **Compound** | **Amount per L** | **Unit** |
| Sodium propionate | 7.2 | g |
| Sodium acetate trihydrate | 10.2 | g |
| Yeast Extract | 1.00 | g |
| Stock I | 20 | ml |
| Stock II | 20 | ml |
| Methanol (liquid) | 8.0 | g |
| Vitamin B solution | 1 | ml |
| Trace element 1 | 0.5 | ml |
| Trace element 2 | 0.5 | ml |

###

## Composition of stock solutions

The composition of the stock solutions is given in the tables below.

| **Table S4**. Composition of “Stock I” solution. | |
| --- | --- |
| **Compound** | **Concentration (g/L)** |
| NH_4_H_2_PO_4_ | 180.0 |
| MgCl_2_*6H_2_O | 16.5 |
| MgSO_4_*7H_2_O | 10.0 |

| **Table S5**. Composition of “Stock II” solution. | |
| --- | --- |
| **Compound** | **Concentration (g/L)** |
| KCl | 7.5 |
| CaCl_2_*2H_2_O | 10.0 |

| **Table S6**. Composition of “Vitamin solution”. | |
| --- | --- |
| **Compound** | **Concentration (g/L)** |
| Biotin | 35.33 |
| Folic acid | 1.67 |
| Pyridoxal-HCl | 0.83 |
| Lipoic acid | 5.00 |
| Riboflavin | 4.17 |
| Thiamine HCl | 88.67 |
| Ca-D-Pantothenate | 137.67 |
| Cyanocobalamin | 4.17 |
| P-aminobenzoic acid | 4.17 |
| Nicotinic acid | 4.17 |

| **Table S7**. Composition of “Trace element I” solution. | |
| --- | --- |
| **Compound** | **Concentration (g/L)** |
| FeCl_2_*4H_2_O | 10000 |
| HCl (1 M) |  |
| MnCl_2_*4H_2_O | 200 |
| H_3_BO_3_ | 2000 |
| CoCl_2_*6H_2_O | 1333 |
| CuCl_2_*H_2_O | 67 |
| NiCl_2_*6H_2_O | 133 |
| ZnSO_4_*7H_2_O | 667 |

| **Table S8**. Composition of “Trace element II” solution. | |
| --- | --- |
| **Compound** | **Concentration (g/L)** |
| Na_2_MoO_4_*2H_2_O | 200 |
| Na_2_SeO_3_ | 67 |
| NaOH (4 M) | 167 |

## Calculation of the concentration dissolved CO­_2_

When CO_2_ comes in contact with water, the following reaction takes place:

$$CO_{2}+H_{2}O\to H_{2}CO_{3}$$

Carbonic acid dissociates into HCO_3_^-^ and CO_3_^2-^:

$$H_{2}CO_{3}\to HCO_{3}^{-}+H^{+} with pK_{A_{1}}=6.35$$

$HCO_{3}^{-}\to CO_{3}^{2-}+H^{+} with pK_{A_{2}}=10.33$ [65]

This gives the following equations (with all concentrations in M):

| $K_{A_{1}}=\frac{\left[ H^{+} \right]*[HCO_{3}^{-}]}{[H_{2}CO_{3}]}$ | Equation 1 |
| --- | --- |
| $K_{A_{2}}=\frac{\left[ H^{+} \right]*[CO_{3}^{2-}]}{[HCO_{3}^{-}]}$ | *Equation 2* |

The total dissolved inorganic carbon is given by:

| $\left[ total inorganic carbon \left( aq \right) \right]=\left[ HCO_{3}^{-} \right]+\left[ CO_{3}^{2-} \right]+[H_{2}CO_{3}]$ | Equation 3 |
| --- | --- |

H_2_CO_3_ and CO_3_^2-^ can be expressed in H^+^, HCO_3_^-^ and K_A_ from Equation 1 and Equation 2:

| $\left[ H_{2}CO_{3} \right]=\frac{\left[ H^{+} \right]*[HCO_{3}^{-}]}{K_{A_{1}}}$ | Equation 4 |
| --- | --- |
| $\left[ CO_{3}^{2-} \right]=\frac{K_{A_{2}}*[HCO_{3}^{-}]}{[H^{+}]}$ | Equation 5 |

Combining Equation 3, Equation 4 and Equation 5 gives:

| $\left[ total inorganic carbon \left( aq \right) \right]=\left[ HCO_{3}^{-} \right]+\frac{K_{A_{2}}*[HCO_{3}^{-}]}{[H^{+}]}+\frac{\left[ H^{+} \right]*[HCO_{3}^{-}]}{K_{A_{1}}}$ | *Equation 6* |
| --- | --- |

Dividing [H_2_CO_3_] (Equation 4) by the total CO_2_ (Equation 6) gives:

$$\frac{[H_{2}CO_{3}]}{[total inorganic carbon \left( aq \right)]}=\frac{\frac{\left[ H^{+} \right]*[HCO_{3}^{-}]}{K_{A_{1}}}}{\left[ HCO_{3}^{-} \right]+\frac{K_{A_{2}}*[HCO_{3}^{-}]}{[H^{+}]}+\frac{\left[ H^{+} \right]*[HCO_{3}^{-}]}{K_{A_{1}}}}$$

Simplifying gives:

$$\frac{[H_{2}CO_{3}]}{[total inorganic carbon \left( aq \right)]}=\frac{\left[ H^{+} \right]^{2}}{\left[ H^{+} \right]^{2}+K_{A_{1}}*\left[ H^{+} \right]+K_{A_{1}}*K_{A_{2}}}$$

The total dissolved inorganic carbon can be calculated:

$$\left[ total inorganic carbon \left( aq \right) \right]=\frac{\left[ H_{2}CO_{3} \right]*\left( \left[ H^{+} \right]^{2}+K_{A_{1}}*\left[ H^{+} \right]+K_{A_{2}}*K_{A_{1}} \right)}{\left[ H^{+} \right]^{2}}$$

With ([H^+^] in M):

$$\left[ H^{+} \right]={10}^{-pH}$$

And:

$$\left[ H_{2}CO_{3}(M) \right]=\frac{\gamma_{CO2}\left( - \right)*P_{tot}(atm)}{K_{H}}$$

γ_CO2_ = fraction CO_2_  in the headspace

P_tot_ = total pressure in the batch bottle

K_H_ = Henry constant = 29.41 atm/M [66]

## Calculation of the required CO_2_ inflow

The concentration of total dissolved CO_2_ at t=1 ([CO_2_]_1_ in M) was calculated using the equations above and the CO_2_ concentration in the headspace of the bioreactor (F_CO2,1_ in atm*M^2^) with a known CO_2_ inflow rate (R_CO2,1_ in ml/min) and pH (pH_1_). For the total pressure (P_tot_), 1 atm was assumed. Changing the pH will change the amount of total dissolved CO_2_. To maintain the same concentration of total dissolved [CO_2_], the CO_2_ supply needs to be changed. The fraction of CO_2_ (F_CO2,2_) needed in the headspace at pH_2_ and t=2 can be calculated from [CO_2_]_2_=[CO_2_]_1_:

$$F_{CO2,2}=\frac{\left[ total CO_{2}\left( aq \right) \right]_{2}*K_{H}*{\left[ H^{+} \right]_{2}}^{2}}{P_{tot}*\left( {\left[ H^{+} \right]_{2}}^{2}+K_{A_{1}}*\left[ H^{+} \right]_{2}+{K_{A}}_{1}*K_{A_{2}} \right)}$$

The rate of CO_2_ supply needed to provide this concentration in the headspace was calculated by:

$$R_{CO2,2}=\frac{R_{CO2,1}*F_{CO2,2}}{F_{CO2,1}}$$

| 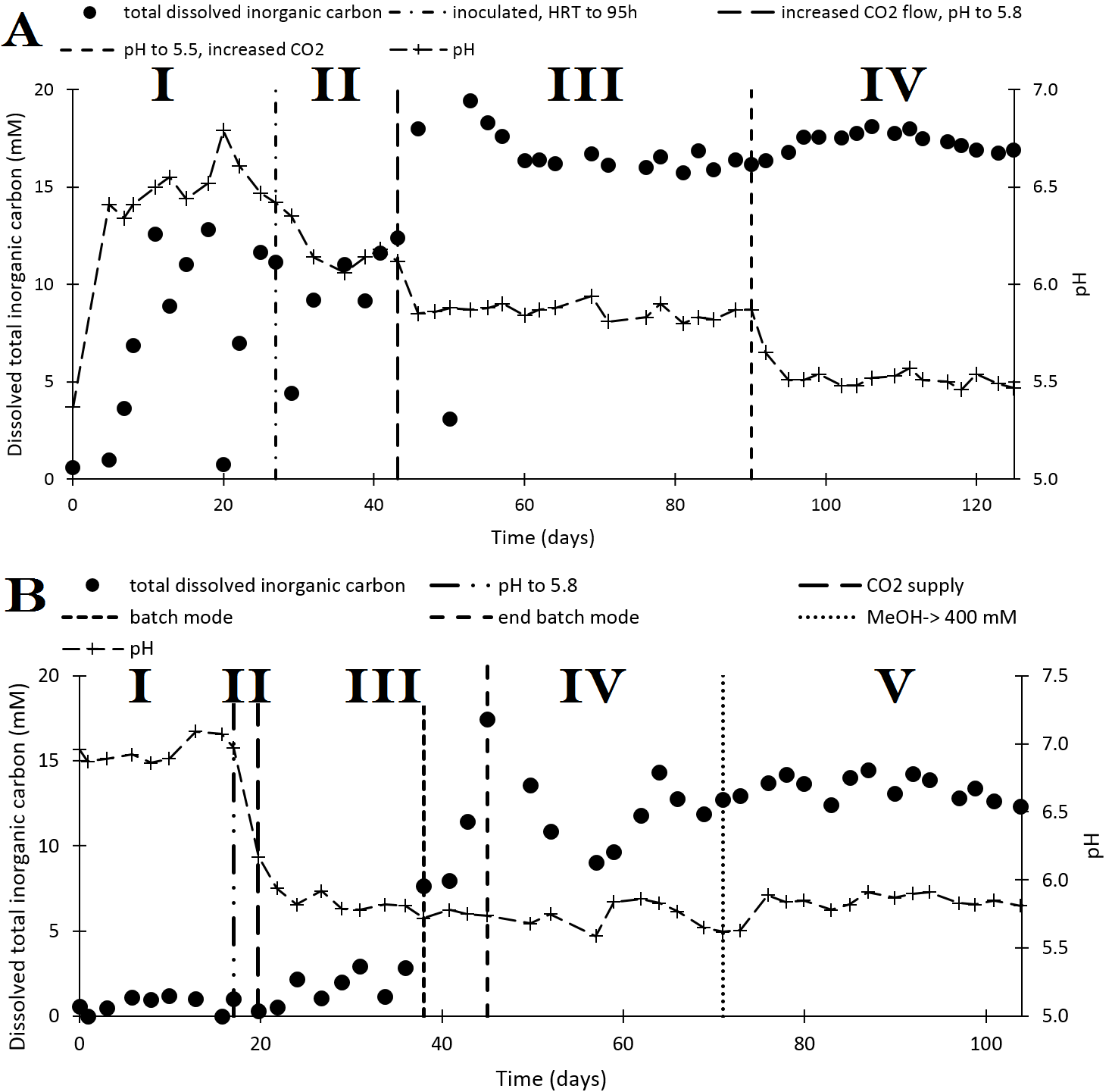 |
| --- |
| **Figure S1.** Change of the concentration of dissolved total inorganic carbon in the reactor and pH in time during the different phases in the continuous open culture reactors with methanol based propionate elongation (A) and simultaneous methanol based elongation of propionate and acetate (B). The vertical lines indicate the major setup changes. The required CO_2_ inflow was calculated as described above. |

## Calculation of Gibbs free energy

| $a\left[ W \right]+b\left[ X \right]\to c\left[ Y \right]+d\left[ Z \right]$ | Equation 7 |
| --- | --- |
| $\Delta_{r}G^{0}=c\Delta_{f}G_{Y}^{0}+d\Delta_{f}G_{Z}^{0}-a\Delta_{f}G_{W}^{0}-d\Delta_{f}G_{X}^{0}$ | Equation 8 |
| $\Delta_{r}G=\Delta_{r}G^{0}+RTln\frac{\left[ Y \right]^{c}\left[ Z \right]^{d}}{\left[ W \right]^{a}\left[ X \right]^{b}}$ | Equation 9 |

The Gibbs free energy from a reaction (Equation 7) can be calculated from the formation energy of the substrates and products from the reaction (Table S9), as shown in Equation 8. When the concentrations of the substrates and products are taken into account, the Gibbs free energy for a reaction can be calculated as shown in Equation 9, with R the gas constant (kJ/(K*mol)) and T the temperature (K).

| **Table S9.** Gibbs free energy values used for the calculation of the Gibbs reaction energy values given in Table 1. | | | |
| --- | --- | --- | --- |
| **Compound** | **Name** | **Phase** | **G_f_^0^ (kJ/mol)** |
| H^+^ | Proton | Aq | 0.0 [67] |
| H_2_ | Hydrogen | G | 0.0 [67] |
| H_2_O | Water | L | -237.2 [67] |
| CHO_3_^-^ | Bicarbonate | Aq | -586.9 [68] |
| CH_4_ | Methane | G | -50.8 [67] |
| CH_4_O | Methanol | Aq | -175.4 [68] |
| C_2_H_3_O_2_^-^ | Acetate | Aq | -369.4 [68] |
| C_3_H_5_O_3_^-^ | Propionate | Aq | -361.1 [68] |
| C_4_H_7_O_2_^-^ | Butyrate | Aq | -352.6 [68] |
| C_5_H_9_O_2_^-^ | Valerate | Aq | -344.3 [67] |

## Concentration and conversion profile of batch experiments with initial pH from 5 to 7.5

|  |
| --- |
| 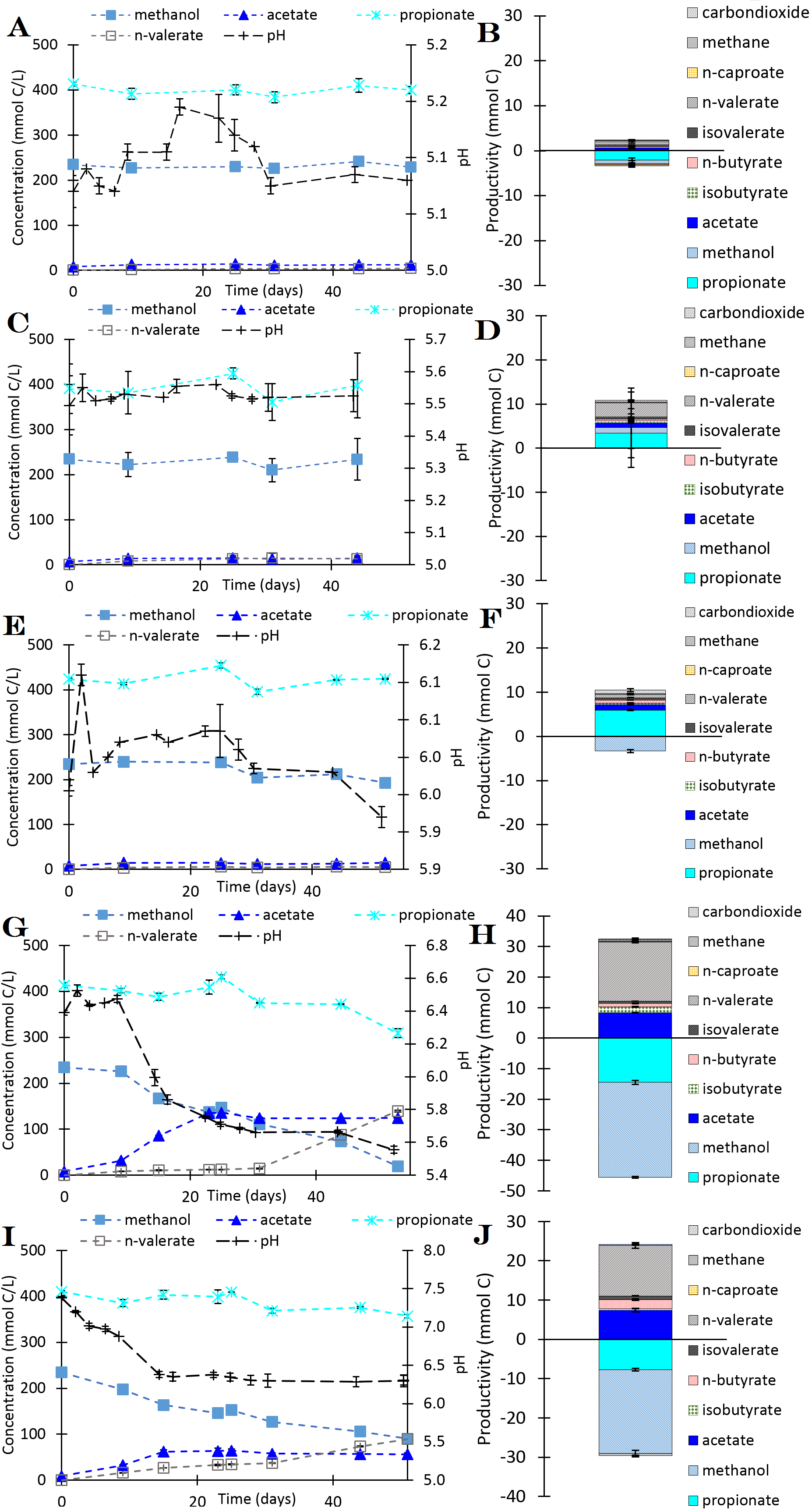 |
| **Figure S2.** Concentration profiles and total conversions during batch experiments with methanol and propionate with initial pH values of 5.0 (A and B), 5.5 (C and D), 6.0 (E and F), 6.5 (G and H) and 7.5 (I and J) at 308 K. The error bars represent the minimum and maximum values measured in the duplo experiments. |

## Concentration profiles of continuous methanol based chain elongation reactors

| 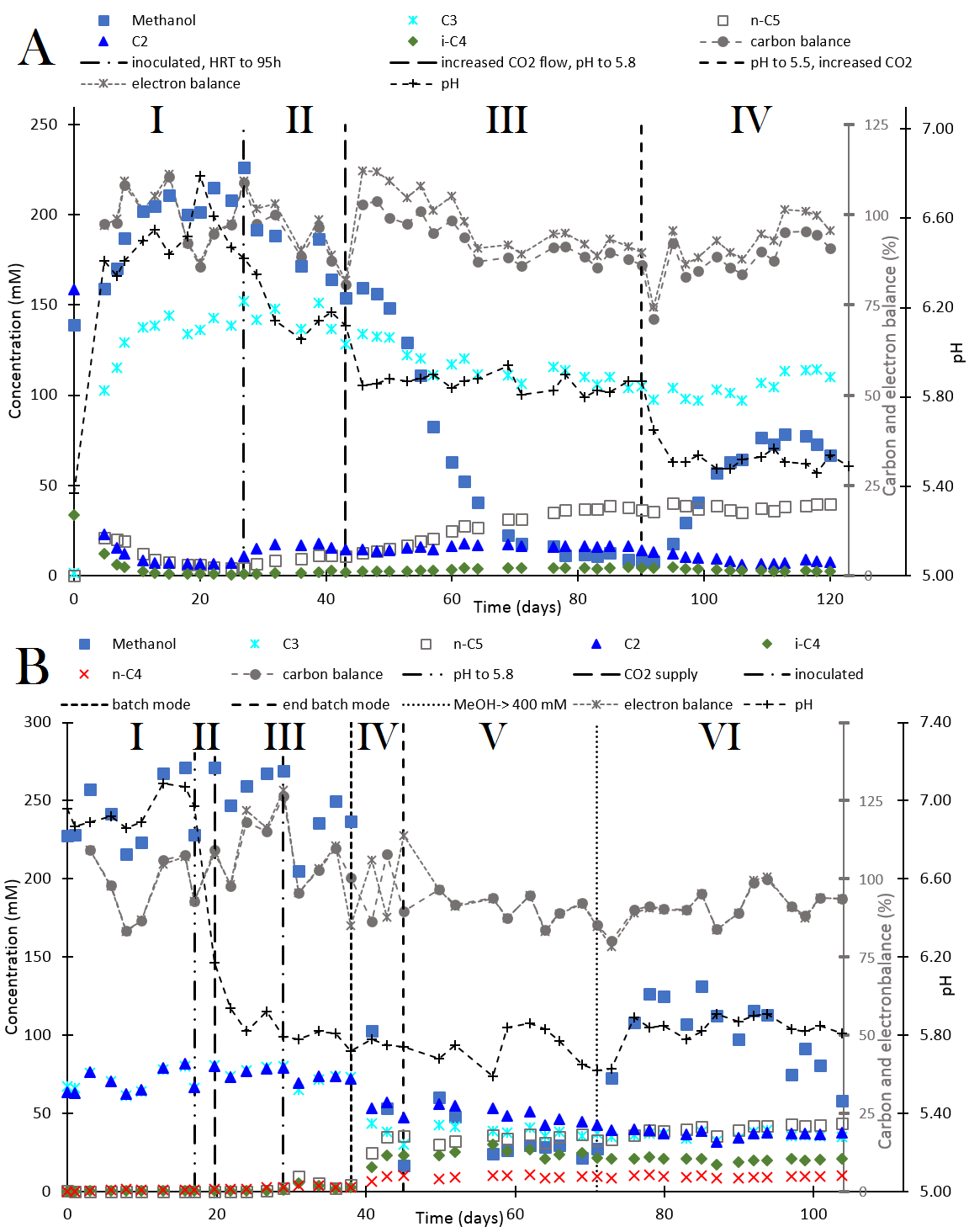 |
| --- |
| ***Figure S3.*** *Change in concentrations of methanol, acetate (C2), propionate (C3), n-butyrate (n-C4), iso-butyrate (i-C4) and n-valerate (n-C5) in time during continuous methanol based* ***propionate*** *elongation (A) and simultaneous* ***acetate*** *and* ***propionate*** *elongation (B). The change of the pH, the carbon balance and the electron balance are shown as well. The vertical lines indicate the major setup changes. The carbon balance was calculated by dividing the total amount of carbon in mmol C/day in the liquid and gas effluent streams over the total amount of carbon in mmol C/day in the liquid and gas influent. The electron balance was calculated by dividing the total amount of electrons in mmol e/day in the outgoing liquid and gas stream by the total amount of electrons in mmol e/day in the ingoing liquid stream. The amount of electrons per compound is equal to the degree of reduction of that compound. The compounds taken into account for the carbon and electron balance are: methanol, ethanol, propanol, butanol, pentanol, hexanol, acetate, propionate, iso-butyrate, n-butyrate, isovalerate, n-valerate, isocaproate, caproate, heptylate, caprylate, methane and carbon dioxide.* |

## Volumetric productivities during continuous methanol based elongation of acetate and propionate

| 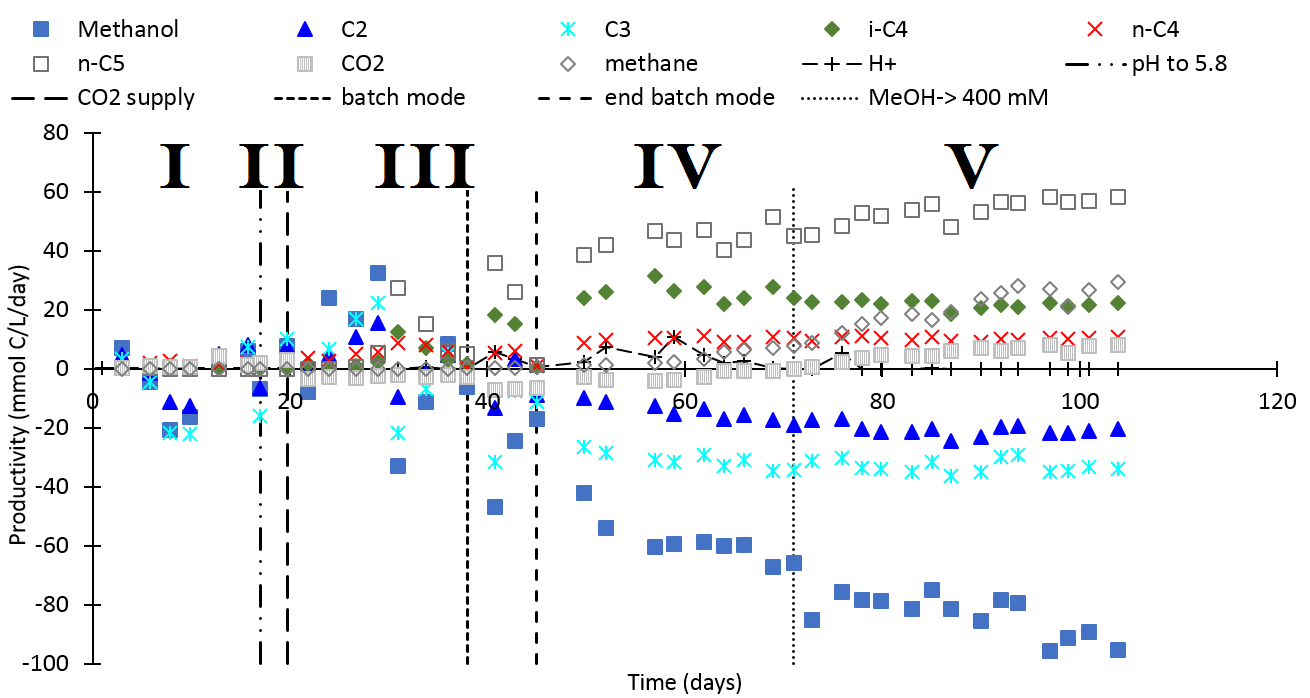 |
| --- |
| **Figure S4.** Volumetric productivities of methane, carbon dioxide (CO2), methanol, acetate (C2), propionate (C3), n-butyrate (n-C4), iso-butyrate (i-C4) and n-valerate (n-C5) in time during continuous **methanol** based elongation of **acetate** and **propionate** in an anaerobic open-culture reactor at 309 K. The production of protons in mmol/day is also shown (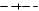). |

| Results of the microbial analysis ***Table S10.*** *Relative abundances of the genera within the microbiome shown in a heat map. At the bottom the total OTU counts are given. The samples were taken at the end of the steady states of phase III (day 90) (pH 5.8) and phase IV (day 125) (pH 5.5) of continuous* ***methanol*** *based* ***propionate*** *elongation in an anaerobic open-culture reactor and at the end of the steady state of phase V (day 69) (methanol in influent 250 mM) and during the last day of phase VI (day 106) (methanol in influent 400 mM) of continuous* ***methanol*** *based* ***propionate and acetate*** *elongation in an anaerobic open-culture reactor at 309 K. The inocula samples from the continuous methanol based acetate elongation reactor and the propionate elongation batch experiment with initial pH 7 (Figure 1) are shown as well. For all biomass samples the duplo results are shown indicated by 1 &2. The inoculum was kept anaerobically on room temperature for three months prior to inoculating the chain elongation reactor. Therefore it should be emphasized that the measured microbiome composition no longer completely represents the composition at the end of the presented batch (Figure 1, day 50). It does, however, provide good reference data with which enrichment of important microorganisms within the continuous system can be identified.* | | | | | | | | | | | | | | |
| --- | --- | --- | --- | --- | --- | --- | --- | --- | --- | --- | --- | --- | --- | --- |
| Order | Family | Genus | Inoculum: continuous methanol based acetate elongation | | Inoculum: Propionate; pH 5.8 (Figure 1) | | Propionate; **pH 5.8**; 250 mM methanol | | Propionate; **pH 5.5**; 250 mM methanol | | Propionate&acetate; pH 5.8; **250 mM methanol** | | Propionate&acetate; pH 5.8; **400 mM methanol** | |
|  |  |  | 1 | 2 | 1 | 2 | 1 | 2 | 1 | 2 | 1 | 2 | 1 | 2 |
| ***Clostridiales*** |  |  | **74.4%** | **72.9%** | **35.3%** | **36.0%** | **42.3%** | **43.2%** | **39.1%** | **46.7%** | **58.1%** | **58.4%** | **38.0%** | **34.6%** |
|  | *Clostridiaceae 1* | *Clostridium sensu stricto 12* | 63.1% | 59.6% | 3.3% | 3.8% | 20.9% | 21.0% | 15.7% | 23.8% | 42.3% | 45.8% | 18.6% | 16.7% |
|  |  | *Oxobacter* | 0.1% | 0.2% | 4.2% | 4.0% | 0.0% | 0.0% | 0.2% | 0.1% | 0.8% | 0.7% | 0.0% | 0.1% |
|  | *Ruminococcaceae* | *Caproiciproducens* | 4.0% | 4.6% | 2.9% | 3.2% | 1.8% | 2.0% | 3.5% | 3.2% | 0.8% | 0.8% | 0.8% | 0.9% |
|  |  | *Oscillibacter* | 1.6% | 1.8% | 3.1% | 3.7% | 1.8% | 1.4% | 0.9% | 0.8% | 1.4% | 1.3% | 2.2% | 2.7% |
|  |  | *uncultured* | 0.5% | 0.7% | 0.3% | 0.3% | 0.3% | 0.3% | 0.3% | 0.2% | 0.3% | 0.3% | 0.2% | 0.2% |
|  |  | *Ruminiclostridium 1* | 0.4% | 0.4% | 2.6% | 3.0% | 1.6% | 1.6% | 4.6% | 4.4% | 2.3% | 2.3% | 3.6% | 3.6% |
|  |  | *Ruminococcaceae NK4A214 group* | 0.2% | 0.2% | 0.1% | 0.2% | 1.1% | 1.3% | 0.9% | 0.8% | 0.1% | 0.1% | 0.1% | 0.1% |
|  |  | *Anaerotruncus* | 0.1% | 0.2% | 0.1% | 0.1% | 0.6% | 0.6% | 0.6% | 0.5% | 0.3% | 0.3% | 0.4% | 0.5% |
|  | *Family XI* | *uncultured* | 2.3% | 3.0% | 5.5% | 4.7% | 3.3% | 3.8% | 9.1% | 9.4% | 0.8% | 0.5% | 1.4% | 1.4% |
|  |  | *Sporanaerobacter* | 1.1% | 1.4% | 2.8% | 2.9% | 4.0% | 4.3% | 1.8% | 1.8% | 7.0% | 4.4% | 7.8% | 5.3% |
|  | *Eubacteriaceae* | *Eubacterium* | 0.0% | 0.0% | 1.4% | 1.2% | 0.3% | 0.3% | 0.0% | 0.0% | 0.0% | 0.0% | 0.1% | 0.2% |
|  | *Lachnospiraceae* | *Lachnospiraceae UCG-010* | 0.0% | 0.0% | 0.0% | 0.0% | 0.5% | 0.5% | 0.1% | 0.0% | 0.0% | 0.0% | 0.1% | 0.1% |
|  | *Peptococcaceae* | *uncultured* | 0.0% | 0.0% | 6.4% | 6.1% | 2.7% | 2.7% | 0.2% | 0.3% | 0.0% | 0.0% | 0.1% | 0.1% |
|  | *Other* | *Other* | *0.9%* | *0.9%* | *2.5%* | *2.9%* | *3.4%* | *3.4%* | *1.3%* | *1.2%* | *1.9%* | *1.8%* | *2.6%* | *2.9%* |
| ***Coriobacteriales*** |  |  | **1.9%** | **2.3%** | **31.2%** | **28.4%** | **2.0%** | **2.2%** | **4.3%** | **3.9%** | **0.4%** | **0.4%** | **1.1%** | **1.0%** |
|  | *Coriobacteriaceae* | *uncultured* | 0.9% | 1.2% | 30.6% | 27.8% | 1.3% | 1.5% | 3.3% | 3.2% | 0.3% | 0.3% | 1.0% | 0.9% |
|  | *Other* | *Other* | 0.9% | 1.0% | 0.2% | 0.3% | 0.6% | 0.6% | 0.9% | 0.5% | 0.1% | 0.1% | 0.0% | 0.0% |
| ***Thermoplasmatales (Archaea)*** |  |  | **6.2%** | **6.2%** | **11.0%** | **13.2%** | **29.3%** | **32.7%** | **25.0%** | **21.6%** | **18.1%** | **19.2%** | **33.9%** | **38.2%** |
|  | *Thermoplasmatales Incertae Sedis* | *Candidatus Methanogranum* | 6.2% | 6.2% | 11.0% | 13.2% | 29.3% | 32.7% | 25.0% | 21.6% | 18.1% | 19.2% | 33.9% | 38.2% |
| ***Synergistales*** |  |  | **0.0%** | **0.0%** | **9.3%** | **7.4%** | **0.8%** | **1.0%** | **0.4%** | **0.4%** | **0.3%** | **0.2%** | **0.3%** | **0.3%** |
|  | *D_4__Synergistaceae* | *D_5__Aminobacterium* | 0.0% | 0.0% | 9.3% | 7.4% | 0.8% | 1.0% | 0.4% | 0.4% | 0.3% | 0.2% | 0.3% | 0.3% |
| ***Burkholderiales*** |  |  | **0.8%** | **0.7%** | **2.8%** | **3.0%** | **1.3%** | **0.7%** | **2.1%** | **1.9%** | **1.0%** | **0.9%** | **1.2%** | **1.3%** |
|  | *D_4__Alcaligenaceae* | *D_5__Sutterella* | 0.7% | 0.7% | 2.3% | 2.5% | 0.0% | 0.1% | 2.1% | 1.8% | 0.8% | 0.7% | 1.2% | 1.2% |
|  | *D_4__Alcaligenaceae* | *D_5__Alcaligenes* | 0.0% | 0.0% | 0.5% | 0.5% | 1.0% | 0.5% | 0.0% | 0.0% | 0.2% | 0.2% | 0.0% | 0.0% |
|  | *Other* | *Other* | *0.0%* | *0.1%* | *0.0%* | *0.0%* | *0.3%* | *0.1%* | *0.0%* | *0.0%* | *0.1%* | *0.1%* | *0.0%* | *0.0%* |
| ***Bacteroidales*** |  |  | **8.3%** | **9.6%** | **2.5%** | **2.8%** | **3.4%** | **4.0%** | **12.1%** | **11.5%** | **8.5%** | **9.0%** | **10.3%** | **10.3%** |
|  | *Rikenellaceae* | *Rikenellaceae RC9 gut group* | 4.0% | 4.2% | 1.0% | 1.2% | 1.3% | 1.6% | 3.7% | 3.0% | 3.2% | 3.9% | 3.1% | 3.2% |
|  | *Prevotellaceae* | *Prevotella* | 3.0% | 3.7% | 0.1% | 0.1% | 1.4% | 1.6% | 7.5% | 7.6% | 4.6% | 4.6% | 5.7% | 5.4% |
|  |  | *Prevotella 7* | 1.1% | 1.6% | 0.1% | 0.2% | 0.0% | 0.0% | 0.3% | 0.4% | 0.0% | 0.0% | 0.0% | 0.0% |
|  | *Porphyromonadaceae* | *Parabacteroides* | 0.0% | 0.0% | 0.2% | 0.2% | 0.5% | 0.5% | 0.3% | 0.2% | 0.4% | 0.3% | 0.9% | 1.1% |
|  |  | *Proteiniphilum* | 0.0% | 0.0% | 0.3% | 0.4% | 0.2% | 0.1% | 0.2% | 0.1% | 0.0% | 0.0% | 0.3% | 0.4% |
|  |  | *Petrimonas* | 0.0% | 0.0% | 0.7% | 0.8% | 0.0% | 0.0% | 0.0% | 0.0% | 0.0% | 0.0% | 0.0% | 0.0% |
|  | *Other* | *Other* | *0.1%* | *0.2%* | *0.0%* | *0.0%* | *0.1%* | *0.1%* | *0.1%* | *0.1%* | *0.2%* | *0.2%* | *0.2%* | *0.2%* |
| ***Spirochaetales*** |  |  | **0.4%** | **0.4%** | **2.1%** | **2.1%** | **0.5%** | **0.5%** | **0.5%** | **0.4%** | **0.4%** | **0.5%** | **0.5%** | **0.4%** |
|  | *Spirochaetaceae* | *Sphaerochaeta* | 0.4% | 0.4% | 2.1% | 2.1% | 0.5% | 0.5% | 0.5% | 0.4% | 0.4% | 0.5% | 0.5% | 0.4% |
| ***Methanomicrobiales (Archaea)*** |  |  | **0.3%** | **0.4%** | **1.6%** | **2.0%** | **2.2%** | **2.8%** | **5.1%** | **3.3%** | **0.2%** | **0.3%** | **4.5%** | **4.0%** |
|  | *Methanomicrobiaceae* | *Methanoculleus* | 0.3% | 0.4% | 1.6% | 2.0% | 2.2% | 2.8% | 5.1% | 3.3% | 0.2% | 0.3% | 4.5% | 4.0% |
| ***Methanobacteriales (Archaea)*** |  |  | **0.0%** | **0.0%** | **1.5%** | **1.9%** | **0.1%** | **0.1%** | **0.1%** | **0.1%** | **0.0%** | **0.0%** | **0.0%** | **0.0%** |
|  | *Methanobacteriaceae* | *Methanobacterium* | 0.0% | 0.0% | 1.5% | 1.9% | 0.0% | 0.0% | 0.0% | 0.0% | 0.0% | 0.0% | 0.0% | 0.0% |
| ***Erysipelotrichales*** |  |  | **4.4%** | **4.2%** | **0.5%** | **0.6%** | **0.5%** | **0.5%** | **7.6%** | **6.6%** | **9.4%** | **8.2%** | **8.3%** | **8.0%** |
|  | *Erysipelotrichaceae* | *Erysipelotrichaceae UCG-004* | 3.6% | 3.2% | 0.2% | 0.3% | 0.4% | 0.4% | 6.8% | 5.9% | 9.3% | 8.1% | 8.1% | 7.7% |
|  | *Other* | *Other* | *0.8%* | *1.1%* | *0.3%* | *0.3%* | *0.2%* | *0.1%* | *0.9%* | *0.7%* | *0.1%* | *0.1%* | *0.2%* | *0.3%* |
| ***Pseudomonadales*** |  |  | **0.0%** | **0.0%** | **0.0%** | **0.0%** | **6.3%** | **3.2%** | **0.0%** | **0.0%** | **0.1%** | **0.1%** | **0.0%** | **0.1%** |
|  | *Pseudomonadaceae* | *Pseudomonas* | 0.0% | 0.0% | 0.0% | 0.0% | 6.3% | 3.2% | 0.0% | 0.0% | 0.1% | 0.1% | 0.0% | 0.1% |
| ***Selenomonadales*** |  |  | **1.3%** | **1.2%** | **0.1%** | **0.0%** | **0.3%** | **0.3%** | **1.7%** | **1.7%** | **0.6%** | **0.4%** | **0.3%** | **0.3%** |
|  | *Veillonellaceae* | *Dialister* | 1.3% | 1.2% | 0.1% | 0.0% | 0.1% | 0.1% | 1.7% | 1.7% | 0.6% | 0.4% | 0.3% | 0.3% |
| ***Rhodocyclales*** |  |  | **0.0%** | **0.0%** | **0.0%** | **0.0%** | **4.9%** | **3.3%** | **0.0%** | **0.0%** | **0.0%** | **0.0%** | **0.0%** | **0.0%** |
|  | *Rhodocyclaceae* | *Azoarcus* | 0.0% | 0.0% | 0.0% | 0.0% | 4.7% | 3.2% | 0.0% | 0.0% | 0.0% | 0.0% | 0.0% | 0.0% |
| ***Campylobacterales*** |  |  | **0.0%** | **0.0%** | **0.0%** | **0.0%** | **1.3%** | **1.4%** | **0.0%** | **0.0%** | **0.1%** | **0.1%** | **0.0%** | **0.1%** |
|  | *Campylobacteraceae* | *Arcobacter* | 0.0% | 0.0% | 0.0% | 0.0% | 1.3% | 1.4% | 0.0% | 0.0% | 0.0% | 0.0% | 0.0% | 0.0% |
| Sum of all order |  |  | 98.0% | 98.0% | 98.0% | 97.6% | 95.2% | 95.7% | 97.9% | 98.1% | 97.4% | 97.7% | 98.5% | 98.5% |
| Other orders |  |  | 2.0% | 2.0% | 2.0% | 2.4% | 4.8% | 4.3% | 2.1% | 1.9% | 2.6% | 2.3% | 1.5% | 1.5% |
| Total OTU count |  |  | 199344 | 145130 | 145477 | 140499 | 132846 | 221728 | 197444 | 208429 | 189896 | 194669 | 183205 | 200691 |

| **Table S11.** The table shows the OTU counts as classified within the Clostridium sensu stricto 12 genus. The samples were taken at the end of the steady states of phase III (day 90) (pH 5.8) and phase IV (day 125) (pH 5.5) of continuous **methanol** based **propionate** elongation in an anaerobic open-culture reactor and at the end of the steady state of phase V (day 69) (methanol in influent 250 mM) and during the last day of phase VI (day 106) (methanol in influent 400 mM) of continuous **methanol** based **propionate and acetate** elongation in an anaerobic open-culture reactor at 309 K. The inocula samples from the continuous methanol based acetate elongation reactor and the propionate elongation batch experiment with initial pH 7 (Figure 1) are shown as well. For all biomass samples the duplo results are shown indicated by 1 &2. OTUs where only one hit was found in total in all 12 samples were omitted due to the size of the table. | | | | | | | | | | | | |
| --- | --- | --- | --- | --- | --- | --- | --- | --- | --- | --- | --- | --- |
|  | **OTU counts** | | | | | | | | | | | |
|  | Inoculum: continuous methanol based acetate elongation | | Inoculum: Propionate; pH 5.8 (Figure 1) | | Propionate; **pH 5.8**; 250 mM methanol | | Propionate; **pH 5.5**; 250 mM methanol | | Propionate&acetate; pH 5.8; **250 mM methanol** | | Propionate&acetate; pH 5.8; **400 mM methanol** | |
| **OTU identifiers** | 1 | 2 | 1 | 2 | 1 | 2 | 1 | 2 | 1 | 2 | 1 | 2 |
| **AB509213.1.1431** | 115492 | 77123 | 4606 | 5054 | 26595 | 44552 | 24975 | 42658 | 77725 | 86904 | 32590 | 32224 |
| **CBXI010000035.40.1552** | 1605 | 1125 | 46 | 59 | 356 | 657 | 3908 | 4337 | 3 | 3 | 9 | 15 |
| **GU907811.1.1429** | 2 | 0 | 0 | 0 | 35 | 78 | 570 | 946 | 0 | 0 | 0 | 0 |
| **KM251144.1.1452** | 253 | 229 | 0 | 0 | 6 | 9 | 341 | 318 | 42 | 26 | 43 | 40 |
| **EU828395.1.1425** | 6453 | 6129 | 3 | 6 | 37 | 26 | 156 | 143 | 1159 | 800 | 495 | 406 |
| **New.ReferenceOTU249** | 105 | 153 | 15 | 15 | 101 | 235 | 119 | 185 | 293 | 185 | 243 | 148 |
| **EU307088.1.1475** | 265 | 153 | 8 | 6 | 63 | 88 | 67 | 100 | 116 | 115 | 66 | 44 |
| **New.ReferenceOTU457** | 50 | 66 | 0 | 1 | 8 | 19 | 57 | 41 | 1 | 1 | 0 | 0 |
| **New.CleanUp.ReferenceOTU150215** | 4 | 1 | 0 | 0 | 0 | 8 | 41 | 19 | 0 | 0 | 0 | 0 |
| **New.ReferenceOTU894** | 7 | 17 | 0 | 5 | 48 | 79 | 40 | 40 | 36 | 63 | 94 | 104 |
| **New.CleanUp.ReferenceOTU55863** | 73 | 91 | 0 | 1 | 10 | 7 | 33 | 35 | 36 | 97 | 22 | 41 |
| **New.ReferenceOTU8** | 43 | 14 | 0 | 0 | 0 | 2 | 33 | 19 | 57 | 115 | 39 | 48 |
| **New.ReferenceOTU41** | 26 | 61 | 0 | 0 | 0 | 0 | 32 | 35 | 3 | 8 | 4 | 2 |
| **New.CleanUp.ReferenceOTU6389** | 8 | 3 | 8 | 0 | 17 | 33 | 31 | 48 | 53 | 66 | 32 | 58 |
| **AB818583.1.1382** | 128 | 90 | 4 | 3 | 23 | 37 | 30 | 50 | 78 | 108 | 27 | 35 |
| **New.CleanUp.ReferenceOTU181811** | 24 | 6 | 0 | 3 | 20 | 24 | 28 | 18 | 0 | 0 | 0 | 5 |
| **AB818610.1.1368** | 141 | 61 | 2 | 7 | 27 | 39 | 22 | 52 | 90 | 90 | 34 | 20 |
| **New.CleanUp.ReferenceOTU143098** | 24 | 16 | 49 | 70 | 9 | 16 | 19 | 52 | 3 | 4 | 9 | 10 |
| **JN650261.1.1431** | 4 | 4 | 0 | 1 | 3 | 3 | 19 | 15 | 0 | 0 | 0 | 0 |
| **New.ReferenceOTU822** | 6 | 13 | 0 | 0 | 6 | 5 | 18 | 14 | 2 | 6 | 0 | 2 |
| **New.ReferenceOTU1081** | 78 | 59 | 3 | 1 | 23 | 28 | 17 | 28 | 55 | 41 | 14 | 26 |
| **New.CleanUp.ReferenceOTU179680** | 12 | 24 | 2 | 0 | 7 | 8 | 16 | 36 | 33 | 37 | 34 | 22 |
| **New.ReferenceOTU646** | 41 | 55 | 2 | 3 | 14 | 43 | 15 | 13 | 61 | 39 | 37 | 45 |
| **New.CleanUp.ReferenceOTU99082** | 3 | 7 | 0 | 0 | 2 | 1 | 15 | 7 | 19 | 13 | 8 | 8 |
| **New.CleanUp.ReferenceOTU179467** | 1 | 3 | 38 | 37 | 7 | 3 | 13 | 20 | 3 | 2 | 6 | 9 |
| **New.CleanUp.ReferenceOTU196169** | 0 | 0 | 0 | 0 | 3 | 5 | 10 | 0 | 1 | 6 | 4 | 7 |
| **New.CleanUp.ReferenceOTU235970** | 22 | 19 | 0 | 1 | 6 | 9 | 9 | 15 | 12 | 15 | 3 | 4 |
| **New.CleanUp.ReferenceOTU5879** | 0 | 2 | 13 | 31 | 56 | 143 | 8 | 9 | 0 | 0 | 0 | 1 |
| **New.ReferenceOTU579** | 17 | 14 | 1 | 0 | 2 | 16 | 8 | 6 | 5 | 3 | 1 | 0 |
| **New.CleanUp.ReferenceOTU234587** | 4 | 1 | 0 | 1 | 3 | 4 | 8 | 11 | 0 | 3 | 0 | 2 |
| **DQ168831.1.1438** | 3 | 6 | 0 | 0 | 0 | 2 | 8 | 14 | 0 | 0 | 0 | 0 |
| **New.CleanUp.ReferenceOTU79711** | 1 | 1 | 0 | 0 | 0 | 0 | 8 | 6 | 5 | 1 | 0 | 0 |
| **New.CleanUp.ReferenceOTU180827** | 10 | 10 | 3 | 1 | 1 | 6 | 7 | 5 | 11 | 11 | 3 | 2 |
| **New.CleanUp.ReferenceOTU14440** | 0 | 0 | 0 | 0 | 0 | 0 | 7 | 7 | 0 | 0 | 0 | 0 |
| **New.CleanUp.ReferenceOTU73562** | 1 | 4 | 0 | 0 | 1 | 1 | 7 | 0 | 0 | 0 | 0 | 0 |
| **New.CleanUp.ReferenceOTU234863** | 0 | 0 | 0 | 0 | 1 | 2 | 7 | 3 | 0 | 0 | 0 | 0 |
| **New.CleanUp.ReferenceOTU118867** | 0 | 0 | 0 | 0 | 0 | 0 | 7 | 0 | 0 | 0 | 0 | 0 |
| **New.ReferenceOTU969** | 36 | 51 | 3 | 4 | 8 | 6 | 6 | 4 | 10 | 11 | 3 | 8 |
| **New.CleanUp.ReferenceOTU148182** | 6 | 5 | 2 | 2 | 10 | 16 | 6 | 4 | 6 | 2 | 15 | 7 |
| **New.CleanUp.ReferenceOTU165501** | 6 | 13 | 0 | 0 | 0 | 1 | 6 | 5 | 1 | 0 | 1 | 0 |
| **New.CleanUp.ReferenceOTU20318** | 0 | 0 | 0 | 1 | 0 | 2 | 6 | 4 | 0 | 0 | 2 | 2 |
| **New.CleanUp.ReferenceOTU151028** | 2 | 2 | 0 | 0 | 0 | 1 | 6 | 1 | 1 | 0 | 0 | 0 |
| **New.CleanUp.ReferenceOTU97716** | 0 | 1 | 0 | 0 | 0 | 1 | 6 | 2 | 0 | 0 | 0 | 0 |
| **New.ReferenceOTU250** | 11 | 4 | 2 | 0 | 7 | 13 | 5 | 3 | 0 | 7 | 3 | 6 |
| **New.ReferenceOTU108** | 9 | 7 | 0 | 0 | 3 | 5 | 5 | 2 | 9 | 10 | 2 | 1 |
| **New.CleanUp.ReferenceOTU128023** | 2 | 0 | 0 | 0 | 0 | 0 | 5 | 17 | 0 | 0 | 0 | 0 |
| **New.CleanUp.ReferenceOTU227971** | 0 | 0 | 6 | 7 | 0 | 4 | 5 | 1 | 0 | 1 | 0 | 0 |
| **New.CleanUp.ReferenceOTU113982** | 0 | 3 | 0 | 0 | 0 | 0 | 5 | 6 | 0 | 0 | 0 | 0 |
| **New.CleanUp.ReferenceOTU40546** | 0 | 0 | 0 | 0 | 1 | 2 | 5 | 1 | 3 | 0 | 0 | 0 |
| **New.CleanUp.ReferenceOTU118741** | 3 | 4 | 0 | 0 | 3 | 0 | 4 | 0 | 3 | 4 | 5 | 6 |
| **New.CleanUp.ReferenceOTU97427** | 0 | 0 | 0 | 0 | 4 | 16 | 4 | 0 | 4 | 1 | 1 | 1 |
| **New.CleanUp.ReferenceOTU127080** | 0 | 0 | 0 | 0 | 2 | 10 | 4 | 0 | 0 | 0 | 2 | 13 |
| **New.ReferenceOTU806** | 6 | 3 | 1 | 0 | 3 | 6 | 4 | 2 | 2 | 2 | 1 | 0 |
| **New.CleanUp.ReferenceOTU64089** | 0 | 0 | 0 | 0 | 1 | 3 | 4 | 0 | 0 | 1 | 5 | 4 |
| **New.CleanUp.ReferenceOTU194750** | 4 | 0 | 0 | 0 | 0 | 0 | 4 | 3 | 2 | 1 | 1 | 1 |
| **New.CleanUp.ReferenceOTU10132** | 3 | 0 | 0 | 0 | 0 | 0 | 4 | 0 | 3 | 3 | 1 | 1 |
| **LN998007.1.1354** | 2 | 0 | 0 | 0 | 0 | 1 | 4 | 2 | 0 | 0 | 0 | 0 |
| **New.CleanUp.ReferenceOTU107051** | 0 | 0 | 0 | 0 | 0 | 1 | 4 | 0 | 0 | 0 | 0 | 0 |
| **New.CleanUp.ReferenceOTU113926** | 4 | 13 | 0 | 0 | 18 | 25 | 3 | 0 | 15 | 13 | 11 | 11 |
| **GQ487959.1.1446** | 50 | 31 | 0 | 1 | 1 | 1 | 3 | 12 | 4 | 0 | 4 | 1 |
| **New.ReferenceOTU804** | 15 | 6 | 0 | 2 | 4 | 8 | 3 | 6 | 6 | 9 | 1 | 1 |
| **KT337634.1.1465** | 7 | 1 | 5 | 1 | 2 | 2 | 3 | 6 | 6 | 2 | 1 | 1 |
| **New.ReferenceOTU1204** | 0 | 3 | 1 | 1 | 0 | 0 | 3 | 7 | 0 | 2 | 1 | 7 |
| **New.CleanUp.ReferenceOTU56379** | 1 | 1 | 0 | 0 | 5 | 2 | 3 | 0 | 0 | 1 | 0 | 1 |
| **New.CleanUp.ReferenceOTU190152** | 0 | 0 | 0 | 0 | 0 | 0 | 3 | 5 | 0 | 0 | 0 | 0 |
| **New.ReferenceOTU413** | 0 | 0 | 0 | 0 | 1 | 0 | 3 | 0 | 0 | 0 | 0 | 0 |
| **New.CleanUp.ReferenceOTU208562** | 0 | 0 | 0 | 0 | 0 | 0 | 3 | 1 | 0 | 0 | 0 | 0 |
| **New.CleanUp.ReferenceOTU46919** | 0 | 0 | 0 | 0 | 0 | 0 | 3 | 0 | 0 | 0 | 0 | 0 |
| **New.CleanUp.ReferenceOTU166594** | 0 | 0 | 0 | 0 | 0 | 0 | 3 | 0 | 0 | 0 | 0 | 0 |
| **New.CleanUp.ReferenceOTU177422** | 0 | 0 | 0 | 0 | 0 | 0 | 3 | 0 | 0 | 0 | 0 | 0 |
| **New.CleanUp.ReferenceOTU157013** | 7 | 9 | 14 | 14 | 0 | 0 | 2 | 4 | 39 | 47 | 0 | 1 |
| **New.CleanUp.ReferenceOTU240726** | 18 | 8 | 0 | 2 | 4 | 5 | 2 | 8 | 12 | 12 | 4 | 4 |
| **New.ReferenceOTU1171** | 2 | 4 | 0 | 0 | 2 | 3 | 2 | 12 | 5 | 18 | 7 | 13 |
| **New.CleanUp.ReferenceOTU10260** | 10 | 6 | 0 | 0 | 0 | 0 | 2 | 0 | 6 | 10 | 4 | 3 |
| **New.CleanUp.ReferenceOTU207538** | 6 | 13 | 1 | 0 | 1 | 2 | 2 | 9 | 0 | 1 | 3 | 1 |
| **New.CleanUp.ReferenceOTU186018** | 0 | 0 | 1 | 2 | 0 | 5 | 2 | 7 | 3 | 2 | 0 | 4 |
| **New.CleanUp.ReferenceOTU42989** | 10 | 2 | 0 | 1 | 0 | 1 | 2 | 3 | 1 | 2 | 2 | 1 |
| **New.ReferenceOTU376** | 0 | 0 | 0 | 0 | 0 | 0 | 2 | 9 | 0 | 0 | 0 | 1 |
| **New.CleanUp.ReferenceOTU16457** | 0 | 1 | 0 | 0 | 0 | 0 | 2 | 2 | 2 | 0 | 0 | 0 |
| **New.CleanUp.ReferenceOTU250762** | 0 | 2 | 0 | 0 | 0 | 0 | 2 | 0 | 0 | 0 | 2 | 0 |
| **New.CleanUp.ReferenceOTU188334** | 0 | 0 | 0 | 0 | 0 | 0 | 2 | 3 | 0 | 0 | 0 | 0 |
| **New.CleanUp.ReferenceOTU200361** | 0 | 0 | 0 | 0 | 0 | 0 | 2 | 3 | 0 | 0 | 0 | 0 |
| **New.CleanUp.ReferenceOTU206932** | 0 | 0 | 0 | 0 | 0 | 0 | 2 | 3 | 0 | 0 | 0 | 0 |
| **New.CleanUp.ReferenceOTU213151** | 0 | 0 | 0 | 0 | 0 | 3 | 2 | 0 | 0 | 0 | 0 | 0 |
| **JN650233.1.1437** | 1 | 0 | 0 | 0 | 0 | 0 | 2 | 1 | 0 | 0 | 0 | 0 |
| **KM251133.1.1417** | 0 | 0 | 0 | 0 | 0 | 0 | 2 | 1 | 0 | 0 | 0 | 0 |
| **New.CleanUp.ReferenceOTU108394** | 0 | 0 | 0 | 0 | 0 | 0 | 2 | 1 | 0 | 0 | 0 | 0 |
| **New.CleanUp.ReferenceOTU157857** | 1 | 0 | 0 | 0 | 0 | 0 | 2 | 0 | 0 | 0 | 0 | 0 |
| **New.CleanUp.ReferenceOTU24964** | 0 | 0 | 0 | 0 | 0 | 0 | 2 | 0 | 0 | 0 | 0 | 0 |
| **New.CleanUp.ReferenceOTU26440** | 0 | 0 | 0 | 0 | 0 | 0 | 2 | 0 | 0 | 0 | 0 | 0 |
| **New.CleanUp.ReferenceOTU66476** | 0 | 0 | 0 | 0 | 0 | 0 | 2 | 0 | 0 | 0 | 0 | 0 |
| **New.CleanUp.ReferenceOTU88160** | 0 | 0 | 0 | 0 | 0 | 0 | 2 | 0 | 0 | 0 | 0 | 0 |
| **New.CleanUp.ReferenceOTU93200** | 0 | 0 | 0 | 0 | 0 | 0 | 2 | 0 | 0 | 0 | 0 | 0 |
| **New.CleanUp.ReferenceOTU175749** | 0 | 0 | 0 | 0 | 0 | 0 | 2 | 0 | 0 | 0 | 0 | 0 |
| **New.CleanUp.ReferenceOTU185891** | 0 | 0 | 0 | 0 | 0 | 0 | 2 | 0 | 0 | 0 | 0 | 0 |
| **New.CleanUp.ReferenceOTU197792** | 0 | 0 | 0 | 0 | 0 | 0 | 2 | 0 | 0 | 0 | 0 | 0 |
| **New.CleanUp.ReferenceOTU223948** | 0 | 0 | 0 | 0 | 0 | 0 | 2 | 0 | 0 | 0 | 0 | 0 |
| **New.CleanUp.ReferenceOTU74697** | 5 | 9 | 1 | 12 | 0 | 0 | 1 | 0 | 31 | 31 | 2 | 1 |
| **New.CleanUp.ReferenceOTU214964** | 4 | 2 | 4 | 5 | 0 | 0 | 1 | 1 | 24 | 25 | 0 | 1 |
| **New.CleanUp.ReferenceOTU89447** | 11 | 8 | 0 | 0 | 0 | 2 | 1 | 9 | 6 | 8 | 9 | 4 |
| **New.CleanUp.ReferenceOTU180386** | 5 | 13 | 0 | 0 | 2 | 4 | 1 | 1 | 5 | 9 | 5 | 0 |
| **New.CleanUp.ReferenceOTU184492** | 6 | 10 | 0 | 0 | 0 | 1 | 1 | 4 | 3 | 11 | 1 | 2 |
| **New.ReferenceOTU889** | 12 | 7 | 0 | 0 | 3 | 9 | 1 | 0 | 0 | 0 | 0 | 6 |
| **New.CleanUp.ReferenceOTU94055** | 9 | 14 | 0 | 0 | 4 | 5 | 1 | 2 | 0 | 0 | 0 | 0 |
| **JN650257.1.1438** | 6 | 1 | 0 | 0 | 2 | 0 | 1 | 0 | 7 | 6 | 1 | 0 |
| **New.CleanUp.ReferenceOTU223315** | 1 | 5 | 0 | 0 | 7 | 7 | 1 | 0 | 0 | 0 | 0 | 0 |
| **New.ReferenceOTU731** | 0 | 2 | 0 | 0 | 0 | 8 | 1 | 5 | 0 | 0 | 0 | 0 |
| **New.CleanUp.ReferenceOTU65807** | 1 | 2 | 0 | 4 | 0 | 0 | 1 | 0 | 5 | 0 | 0 | 0 |
| **New.CleanUp.ReferenceOTU15629** | 2 | 5 | 0 | 0 | 0 | 0 | 1 | 2 | 0 | 0 | 0 | 2 |
| **New.CleanUp.ReferenceOTU116384** | 2 | 0 | 0 | 2 | 0 | 5 | 1 | 1 | 0 | 0 | 1 | 0 |
| **New.CleanUp.ReferenceOTU178615** | 0 | 4 | 0 | 0 | 0 | 1 | 1 | 0 | 0 | 3 | 1 | 0 |
| **New.CleanUp.ReferenceOTU162869** | 5 | 2 | 0 | 0 | 1 | 0 | 1 | 0 | 0 | 0 | 0 | 0 |
| **DQ168846.1.1437** | 0 | 0 | 0 | 0 | 0 | 3 | 1 | 3 | 0 | 0 | 0 | 0 |
| **New.CleanUp.ReferenceOTU77502** | 0 | 2 | 0 | 0 | 1 | 1 | 1 | 2 | 0 | 0 | 0 | 0 |
| **New.CleanUp.ReferenceOTU119512** | 0 | 0 | 0 | 2 | 0 | 1 | 1 | 0 | 1 | 1 | 0 | 1 |
| **New.CleanUp.ReferenceOTU33323** | 1 | 1 | 0 | 0 | 0 | 0 | 1 | 2 | 1 | 0 | 0 | 0 |
| **New.CleanUp.ReferenceOTU101384** | 1 | 1 | 0 | 0 | 0 | 0 | 1 | 0 | 2 | 1 | 0 | 0 |
| **New.CleanUp.ReferenceOTU21944** | 1 | 1 | 0 | 0 | 0 | 0 | 1 | 0 | 2 | 0 | 0 | 0 |
| **New.CleanUp.ReferenceOTU93030** | 0 | 0 | 0 | 0 | 0 | 0 | 1 | 1 | 2 | 1 | 0 | 0 |
| **New.CleanUp.ReferenceOTU145535** | 0 | 2 | 0 | 0 | 0 | 0 | 1 | 1 | 1 | 0 | 0 | 0 |
| **New.CleanUp.ReferenceOTU18864** | 0 | 3 | 0 | 0 | 0 | 0 | 1 | 0 | 0 | 0 | 0 | 0 |
| **New.CleanUp.ReferenceOTU94475** | 1 | 0 | 0 | 0 | 1 | 0 | 1 | 0 | 1 | 0 | 0 | 0 |
| **New.CleanUp.ReferenceOTU107434** | 0 | 3 | 0 | 0 | 0 | 0 | 1 | 0 | 0 | 0 | 0 | 0 |
| **New.CleanUp.ReferenceOTU125957** | 0 | 0 | 0 | 0 | 0 | 0 | 1 | 2 | 0 | 0 | 0 | 0 |
| **New.CleanUp.ReferenceOTU150209** | 1 | 0 | 0 | 0 | 0 | 0 | 1 | 1 | 0 | 0 | 0 | 0 |
| **New.CleanUp.ReferenceOTU185651** | 1 | 0 | 0 | 0 | 0 | 0 | 1 | 0 | 1 | 0 | 0 | 0 |
| **New.CleanUp.ReferenceOTU248204** | 2 | 0 | 0 | 0 | 0 | 0 | 1 | 0 | 0 | 0 | 0 | 0 |
| **GU227148.1.1230** | 0 | 0 | 0 | 0 | 0 | 0 | 1 | 1 | 0 | 0 | 0 | 0 |
| **New.CleanUp.ReferenceOTU2417** | 0 | 1 | 0 | 0 | 0 | 0 | 1 | 0 | 0 | 0 | 0 | 0 |
| **New.CleanUp.ReferenceOTU18341** | 1 | 0 | 0 | 0 | 0 | 0 | 1 | 0 | 0 | 0 | 0 | 0 |
| **New.CleanUp.ReferenceOTU95562** | 0 | 0 | 0 | 0 | 0 | 0 | 1 | 0 | 0 | 0 | 0 | 1 |
| **New.CleanUp.ReferenceOTU170475** | 0 | 0 | 0 | 0 | 0 | 0 | 1 | 0 | 1 | 0 | 0 | 0 |
| **New.ReferenceOTU1158** | 177 | 279 | 0 | 0 | 0 | 0 | 0 | 3 | 28 | 41 | 6 | 4 |
| **New.CleanUp.ReferenceOTU253056** | 108 | 96 | 0 | 0 | 1 | 0 | 0 | 2 | 12 | 7 | 6 | 0 |
| **New.CleanUp.ReferenceOTU22748** | 2 | 3 | 0 | 0 | 12 | 9 | 0 | 1 | 23 | 26 | 15 | 6 |
| **New.CleanUp.ReferenceOTU67681** | 36 | 45 | 0 | 0 | 0 | 0 | 0 | 0 | 7 | 8 | 0 | 0 |
| **New.CleanUp.ReferenceOTU82444** | 4 | 5 | 0 | 0 | 17 | 10 | 0 | 6 | 3 | 7 | 2 | 4 |
| **New.CleanUp.ReferenceOTU117824** | 8 | 29 | 0 | 0 | 1 | 2 | 0 | 1 | 4 | 5 | 0 | 2 |
| **New.CleanUp.ReferenceOTU117170** | 0 | 0 | 2 | 7 | 6 | 26 | 0 | 0 | 0 | 0 | 0 | 0 |
| **New.CleanUp.ReferenceOTU180483** | 1 | 1 | 0 | 0 | 1 | 19 | 0 | 0 | 3 | 2 | 1 | 6 |
| **New.CleanUp.ReferenceOTU127765** | 0 | 1 | 0 | 0 | 10 | 20 | 0 | 0 | 0 | 0 | 0 | 0 |
| **New.CleanUp.ReferenceOTU23120** | 3 | 1 | 4 | 1 | 2 | 5 | 0 | 1 | 3 | 3 | 2 | 1 |
| **New.CleanUp.ReferenceOTU56444** | 0 | 0 | 0 | 0 | 7 | 15 | 0 | 0 | 1 | 0 | 0 | 0 |
| **New.CleanUp.ReferenceOTU152325** | 3 | 3 | 0 | 0 | 1 | 0 | 0 | 0 | 3 | 3 | 1 | 2 |
| **New.CleanUp.ReferenceOTU154094** | 1 | 4 | 0 | 0 | 0 | 5 | 0 | 1 | 0 | 3 | 0 | 0 |
| **New.CleanUp.ReferenceOTU222574** | 0 | 0 | 0 | 0 | 0 | 2 | 0 | 0 | 5 | 3 | 2 | 1 |
| **JQ423945.1.1395** | 6 | 4 | 0 | 0 | 0 | 0 | 0 | 0 | 0 | 0 | 0 | 1 |
| **New.CleanUp.ReferenceOTU79140** | 0 | 2 | 0 | 0 | 0 | 4 | 0 | 4 | 0 | 0 | 1 | 0 |
| **New.CleanUp.ReferenceOTU134554** | 3 | 0 | 0 | 0 | 0 | 2 | 0 | 2 | 0 | 0 | 1 | 2 |
| **New.CleanUp.ReferenceOTU215664** | 2 | 0 | 0 | 0 | 0 | 0 | 0 | 0 | 0 | 7 | 1 | 0 |
| **New.CleanUp.ReferenceOTU229533** | 1 | 4 | 0 | 0 | 0 | 0 | 0 | 3 | 1 | 0 | 0 | 1 |
| **New.CleanUp.ReferenceOTU60665** | 0 | 0 | 0 | 0 | 0 | 0 | 0 | 0 | 0 | 5 | 4 | 0 |
| **New.CleanUp.ReferenceOTU79467** | 3 | 0 | 0 | 0 | 0 | 0 | 0 | 1 | 3 | 2 | 0 | 0 |
| **New.CleanUp.ReferenceOTU81818** | 1 | 1 | 0 | 1 | 0 | 0 | 0 | 1 | 0 | 0 | 2 | 3 |
| **New.CleanUp.ReferenceOTU65973** | 0 | 0 | 0 | 0 | 2 | 6 | 0 | 0 | 0 | 0 | 0 | 0 |
| **New.CleanUp.ReferenceOTU214253** | 0 | 0 | 0 | 1 | 0 | 4 | 0 | 0 | 0 | 0 | 3 | 0 |
| **New.CleanUp.ReferenceOTU224239** | 0 | 0 | 0 | 0 | 1 | 7 | 0 | 0 | 0 | 0 | 0 | 0 |
| **New.ReferenceOTU198** | 0 | 6 | 0 | 0 | 0 | 0 | 0 | 1 | 0 | 0 | 0 | 0 |
| **New.CleanUp.ReferenceOTU16719** | 0 | 0 | 0 | 0 | 0 | 0 | 0 | 0 | 5 | 2 | 0 | 0 |
| **New.CleanUp.ReferenceOTU89906** | 0 | 0 | 0 | 0 | 2 | 2 | 0 | 0 | 0 | 0 | 0 | 3 |
| **New.CleanUp.ReferenceOTU125915** | 0 | 1 | 0 | 0 | 0 | 0 | 0 | 0 | 3 | 2 | 0 | 1 |
| **New.CleanUp.ReferenceOTU199968** | 3 | 4 | 0 | 0 | 0 | 0 | 0 | 0 | 0 | 0 | 0 | 0 |
| **New.CleanUp.ReferenceOTU244529** | 0 | 0 | 0 | 0 | 1 | 0 | 0 | 0 | 0 | 1 | 1 | 4 |
| **New.CleanUp.ReferenceOTU252325** | 0 | 1 | 0 | 0 | 1 | 0 | 0 | 0 | 0 | 4 | 1 | 0 |
| **New.CleanUp.ReferenceOTU2791** | 4 | 2 | 0 | 0 | 0 | 0 | 0 | 0 | 0 | 0 | 0 | 0 |
| **New.CleanUp.ReferenceOTU58613** | 2 | 0 | 0 | 0 | 0 | 0 | 0 | 0 | 0 | 1 | 3 | 0 |
| **New.CleanUp.ReferenceOTU77120** | 0 | 1 | 0 | 0 | 0 | 0 | 0 | 0 | 3 | 2 | 0 | 0 |
| **New.CleanUp.ReferenceOTU77348** | 0 | 0 | 0 | 0 | 2 | 1 | 0 | 0 | 0 | 3 | 0 | 0 |
| **New.CleanUp.ReferenceOTU116481** | 0 | 0 | 0 | 0 | 1 | 4 | 0 | 0 | 0 | 1 | 0 | 0 |
| **New.CleanUp.ReferenceOTU132623** | 0 | 0 | 3 | 0 | 0 | 0 | 0 | 0 | 0 | 2 | 0 | 1 |
| **New.CleanUp.ReferenceOTU134523** | 0 | 0 | 4 | 0 | 0 | 0 | 0 | 0 | 2 | 0 | 0 | 0 |
| **New.CleanUp.ReferenceOTU152929** | 3 | 3 | 0 | 0 | 0 | 0 | 0 | 0 | 0 | 0 | 0 | 0 |
| **New.CleanUp.ReferenceOTU155799** | 2 | 3 | 0 | 0 | 0 | 0 | 0 | 0 | 0 | 0 | 0 | 1 |
| **New.CleanUp.ReferenceOTU160500** | 1 | 4 | 0 | 0 | 0 | 0 | 0 | 0 | 0 | 1 | 0 | 0 |
| **New.CleanUp.ReferenceOTU169518** | 0 | 0 | 0 | 0 | 0 | 3 | 0 | 2 | 1 | 0 | 0 | 0 |
| **New.CleanUp.ReferenceOTU199190** | 1 | 0 | 0 | 1 | 0 | 0 | 0 | 3 | 0 | 0 | 1 | 0 |
| **EU828368.1.1424** | 1 | 0 | 0 | 0 | 0 | 0 | 0 | 4 | 0 | 0 | 0 | 0 |
| **New.CleanUp.ReferenceOTU5583** | 3 | 0 | 0 | 0 | 0 | 0 | 0 | 0 | 0 | 0 | 0 | 2 |
| **New.CleanUp.ReferenceOTU8579** | 1 | 3 | 0 | 0 | 0 | 0 | 0 | 0 | 0 | 0 | 0 | 1 |
| **New.CleanUp.ReferenceOTU39916** | 1 | 1 | 0 | 0 | 1 | 1 | 0 | 0 | 0 | 0 | 1 | 0 |
| **New.CleanUp.ReferenceOTU45010** | 0 | 0 | 0 | 0 | 0 | 0 | 0 | 5 | 0 | 0 | 0 | 0 |
| **New.CleanUp.ReferenceOTU63194** | 5 | 0 | 0 | 0 | 0 | 0 | 0 | 0 | 0 | 0 | 0 | 0 |
| **New.CleanUp.ReferenceOTU104450** | 0 | 2 | 0 | 0 | 0 | 0 | 0 | 3 | 0 | 0 | 0 | 0 |
| **New.CleanUp.ReferenceOTU143039** | 0 | 0 | 0 | 0 | 4 | 1 | 0 | 0 | 0 | 0 | 0 | 0 |
| **New.CleanUp.ReferenceOTU155645** | 0 | 0 | 0 | 0 | 0 | 1 | 0 | 4 | 0 | 0 | 0 | 0 |
| **New.CleanUp.ReferenceOTU180189** | 0 | 0 | 0 | 0 | 0 | 3 | 0 | 0 | 0 | 0 | 2 | 0 |
| **New.CleanUp.ReferenceOTU184704** | 2 | 0 | 0 | 0 | 0 | 3 | 0 | 0 | 0 | 0 | 0 | 0 |
| **New.CleanUp.ReferenceOTU187401** | 0 | 5 | 0 | 0 | 0 | 0 | 0 | 0 | 0 | 0 | 0 | 0 |
| **New.CleanUp.ReferenceOTU187574** | 0 | 0 | 0 | 2 | 1 | 2 | 0 | 0 | 0 | 0 | 0 | 0 |
| **New.CleanUp.ReferenceOTU194404** | 0 | 2 | 0 | 0 | 0 | 0 | 0 | 0 | 2 | 1 | 0 | 0 |
| **New.CleanUp.ReferenceOTU212000** | 0 | 0 | 0 | 0 | 0 | 0 | 0 | 1 | 1 | 3 | 0 | 0 |
| **ASZX01000084.7.1496** | 3 | 0 | 0 | 0 | 0 | 0 | 0 | 1 | 0 | 0 | 0 | 0 |
| **New.ReferenceOTU893** | 0 | 0 | 0 | 0 | 2 | 1 | 0 | 1 | 0 | 0 | 0 | 0 |
| **New.CleanUp.ReferenceOTU3592** | 2 | 0 | 0 | 0 | 0 | 0 | 0 | 0 | 0 | 0 | 1 | 1 |
| **New.CleanUp.ReferenceOTU11833** | 0 | 0 | 0 | 0 | 2 | 2 | 0 | 0 | 0 | 0 | 0 | 0 |
| **New.CleanUp.ReferenceOTU21757** | 1 | 0 | 0 | 0 | 0 | 1 | 0 | 0 | 0 | 2 | 0 | 0 |
| **New.CleanUp.ReferenceOTU29207** | 0 | 0 | 0 | 0 | 2 | 2 | 0 | 0 | 0 | 0 | 0 | 0 |
| **New.CleanUp.ReferenceOTU40426** | 0 | 0 | 0 | 0 | 0 | 0 | 0 | 4 | 0 | 0 | 0 | 0 |
| **New.CleanUp.ReferenceOTU49559** | 1 | 0 | 0 | 0 | 0 | 0 | 0 | 2 | 0 | 1 | 0 | 0 |
| **New.CleanUp.ReferenceOTU74052** | 0 | 1 | 0 | 0 | 0 | 0 | 0 | 0 | 1 | 1 | 1 | 0 |
| **New.CleanUp.ReferenceOTU75943** | 1 | 1 | 0 | 0 | 2 | 0 | 0 | 0 | 0 | 0 | 0 | 0 |
| **New.CleanUp.ReferenceOTU103924** | 0 | 4 | 0 | 0 | 0 | 0 | 0 | 0 | 0 | 0 | 0 | 0 |
| **New.CleanUp.ReferenceOTU113459** | 4 | 0 | 0 | 0 | 0 | 0 | 0 | 0 | 0 | 0 | 0 | 0 |
| **New.CleanUp.ReferenceOTU121554** | 1 | 0 | 0 | 0 | 0 | 0 | 0 | 1 | 0 | 1 | 0 | 1 |
| **New.CleanUp.ReferenceOTU139760** | 0 | 0 | 0 | 0 | 0 | 0 | 0 | 1 | 0 | 0 | 1 | 2 |
| **New.CleanUp.ReferenceOTU141055** | 0 | 0 | 1 | 1 | 0 | 0 | 0 | 0 | 0 | 0 | 2 | 0 |
| **New.CleanUp.ReferenceOTU143376** | 0 | 0 | 0 | 0 | 0 | 0 | 0 | 0 | 0 | 4 | 0 | 0 |
| **New.CleanUp.ReferenceOTU150432** | 0 | 0 | 0 | 0 | 0 | 1 | 0 | 0 | 1 | 1 | 0 | 1 |
| **New.CleanUp.ReferenceOTU154212** | 0 | 0 | 0 | 0 | 0 | 0 | 0 | 4 | 0 | 0 | 0 | 0 |
| **New.CleanUp.ReferenceOTU159295** | 1 | 1 | 0 | 0 | 0 | 0 | 0 | 0 | 2 | 0 | 0 | 0 |
| **New.CleanUp.ReferenceOTU177194** | 0 | 0 | 0 | 0 | 0 | 0 | 0 | 4 | 0 | 0 | 0 | 0 |
| **New.CleanUp.ReferenceOTU197952** | 0 | 0 | 0 | 0 | 0 | 0 | 0 | 0 | 2 | 2 | 0 | 0 |
| **New.CleanUp.ReferenceOTU199886** | 1 | 2 | 0 | 0 | 0 | 0 | 0 | 0 | 0 | 0 | 1 | 0 |
| **New.CleanUp.ReferenceOTU203172** | 2 | 0 | 0 | 0 | 0 | 0 | 0 | 0 | 2 | 0 | 0 | 0 |
| **New.CleanUp.ReferenceOTU212822** | 0 | 0 | 0 | 0 | 4 | 0 | 0 | 0 | 0 | 0 | 0 | 0 |
| **GU227151.1.1472** | 1 | 0 | 0 | 0 | 0 | 1 | 0 | 1 | 0 | 0 | 0 | 0 |
| **AB509218.1.1449** | 1 | 0 | 0 | 0 | 0 | 0 | 0 | 0 | 1 | 1 | 0 | 0 |
| **New.CleanUp.ReferenceOTU13092** | 3 | 0 | 0 | 0 | 0 | 0 | 0 | 0 | 0 | 0 | 0 | 0 |
| **New.CleanUp.ReferenceOTU16955** | 0 | 0 | 0 | 0 | 0 | 0 | 0 | 0 | 3 | 0 | 0 | 0 |
| **New.CleanUp.ReferenceOTU24113** | 0 | 0 | 0 | 0 | 0 | 3 | 0 | 0 | 0 | 0 | 0 | 0 |
| **New.CleanUp.ReferenceOTU28156** | 3 | 0 | 0 | 0 | 0 | 0 | 0 | 0 | 0 | 0 | 0 | 0 |
| **New.CleanUp.ReferenceOTU28873** | 0 | 0 | 0 | 0 | 0 | 0 | 0 | 1 | 2 | 0 | 0 | 0 |
| **New.CleanUp.ReferenceOTU31653** | 0 | 0 | 0 | 0 | 0 | 0 | 0 | 0 | 0 | 3 | 0 | 0 |
| **New.CleanUp.ReferenceOTU32857** | 2 | 0 | 0 | 0 | 0 | 0 | 0 | 0 | 0 | 1 | 0 | 0 |
| **New.CleanUp.ReferenceOTU36239** | 1 | 0 | 0 | 0 | 0 | 0 | 0 | 1 | 0 | 0 | 1 | 0 |
| **New.CleanUp.ReferenceOTU38063** | 0 | 0 | 0 | 0 | 0 | 0 | 0 | 1 | 0 | 0 | 1 | 1 |
| **New.CleanUp.ReferenceOTU38803** | 0 | 0 | 0 | 0 | 0 | 0 | 0 | 0 | 3 | 0 | 0 | 0 |
| **New.CleanUp.ReferenceOTU45847** | 0 | 0 | 0 | 3 | 0 | 0 | 0 | 0 | 0 | 0 | 0 | 0 |
| **New.CleanUp.ReferenceOTU47231** | 0 | 0 | 0 | 0 | 0 | 3 | 0 | 0 | 0 | 0 | 0 | 0 |
| **New.CleanUp.ReferenceOTU51345** | 0 | 0 | 0 | 0 | 0 | 0 | 0 | 2 | 1 | 0 | 0 | 0 |
| **New.CleanUp.ReferenceOTU64711** | 2 | 0 | 0 | 0 | 0 | 1 | 0 | 0 | 0 | 0 | 0 | 0 |
| **New.CleanUp.ReferenceOTU70177** | 0 | 0 | 0 | 0 | 2 | 1 | 0 | 0 | 0 | 0 | 0 | 0 |
| **New.CleanUp.ReferenceOTU70399** | 0 | 0 | 0 | 0 | 1 | 0 | 0 | 0 | 2 | 0 | 0 | 0 |
| **New.CleanUp.ReferenceOTU79991** | 0 | 3 | 0 | 0 | 0 | 0 | 0 | 0 | 0 | 0 | 0 | 0 |
| **New.CleanUp.ReferenceOTU82108** | 1 | 0 | 0 | 0 | 0 | 0 | 0 | 1 | 0 | 0 | 1 | 0 |
| **New.CleanUp.ReferenceOTU82370** | 0 | 0 | 0 | 0 | 3 | 0 | 0 | 0 | 0 | 0 | 0 | 0 |
| **New.CleanUp.ReferenceOTU83840** | 1 | 0 | 0 | 0 | 0 | 1 | 0 | 0 | 0 | 0 | 0 | 1 |
| **New.CleanUp.ReferenceOTU92850** | 2 | 0 | 0 | 0 | 0 | 0 | 0 | 0 | 0 | 1 | 0 | 0 |
| **New.CleanUp.ReferenceOTU93560** | 0 | 0 | 0 | 0 | 0 | 0 | 0 | 3 | 0 | 0 | 0 | 0 |
| **New.CleanUp.ReferenceOTU94604** | 0 | 3 | 0 | 0 | 0 | 0 | 0 | 0 | 0 | 0 | 0 | 0 |
| **New.CleanUp.ReferenceOTU98117** | 0 | 0 | 0 | 0 | 0 | 2 | 0 | 0 | 0 | 0 | 1 | 0 |
| **New.CleanUp.ReferenceOTU101667** | 0 | 0 | 0 | 0 | 0 | 0 | 0 | 1 | 1 | 1 | 0 | 0 |
| **New.CleanUp.ReferenceOTU109131** | 0 | 0 | 0 | 0 | 2 | 1 | 0 | 0 | 0 | 0 | 0 | 0 |
| **New.CleanUp.ReferenceOTU121601** | 0 | 3 | 0 | 0 | 0 | 0 | 0 | 0 | 0 | 0 | 0 | 0 |
| **New.CleanUp.ReferenceOTU122841** | 0 | 0 | 0 | 0 | 0 | 2 | 0 | 1 | 0 | 0 | 0 | 0 |
| **New.CleanUp.ReferenceOTU136276** | 0 | 0 | 0 | 0 | 3 | 0 | 0 | 0 | 0 | 0 | 0 | 0 |
| **New.CleanUp.ReferenceOTU139613** | 0 | 0 | 0 | 3 | 0 | 0 | 0 | 0 | 0 | 0 | 0 | 0 |
| **New.CleanUp.ReferenceOTU148889** | 2 | 0 | 0 | 0 | 0 | 0 | 0 | 0 | 0 | 0 | 1 | 0 |
| **New.CleanUp.ReferenceOTU154221** | 0 | 1 | 0 | 0 | 0 | 1 | 0 | 0 | 0 | 0 | 0 | 1 |
| **New.CleanUp.ReferenceOTU155823** | 0 | 0 | 0 | 0 | 0 | 0 | 0 | 0 | 0 | 3 | 0 | 0 |
| **New.CleanUp.ReferenceOTU157986** | 0 | 0 | 0 | 0 | 0 | 0 | 0 | 1 | 0 | 1 | 1 | 0 |
| **New.CleanUp.ReferenceOTU165038** | 0 | 0 | 0 | 0 | 0 | 3 | 0 | 0 | 0 | 0 | 0 | 0 |
| **New.CleanUp.ReferenceOTU165662** | 0 | 3 | 0 | 0 | 0 | 0 | 0 | 0 | 0 | 0 | 0 | 0 |
| **New.CleanUp.ReferenceOTU170345** | 0 | 1 | 0 | 0 | 0 | 0 | 0 | 0 | 0 | 2 | 0 | 0 |
| **New.CleanUp.ReferenceOTU173101** | 0 | 3 | 0 | 0 | 0 | 0 | 0 | 0 | 0 | 0 | 0 | 0 |
| **New.CleanUp.ReferenceOTU179822** | 2 | 0 | 0 | 0 | 0 | 0 | 0 | 0 | 0 | 1 | 0 | 0 |
| **New.CleanUp.ReferenceOTU180380** | 0 | 2 | 0 | 0 | 0 | 1 | 0 | 0 | 0 | 0 | 0 | 0 |
| **New.CleanUp.ReferenceOTU187107** | 2 | 1 | 0 | 0 | 0 | 0 | 0 | 0 | 0 | 0 | 0 | 0 |
| **New.CleanUp.ReferenceOTU188330** | 0 | 2 | 0 | 0 | 0 | 0 | 0 | 0 | 1 | 0 | 0 | 0 |
| **New.CleanUp.ReferenceOTU193162** | 1 | 1 | 0 | 0 | 0 | 0 | 0 | 1 | 0 | 0 | 0 | 0 |
| **New.CleanUp.ReferenceOTU206326** | 1 | 0 | 0 | 0 | 0 | 0 | 0 | 1 | 0 | 1 | 0 | 0 |
| **New.CleanUp.ReferenceOTU207666** | 0 | 1 | 0 | 0 | 0 | 0 | 0 | 0 | 0 | 2 | 0 | 0 |
| **New.CleanUp.ReferenceOTU214627** | 0 | 0 | 3 | 0 | 0 | 0 | 0 | 0 | 0 | 0 | 0 | 0 |
| **New.CleanUp.ReferenceOTU215361** | 1 | 1 | 0 | 0 | 0 | 0 | 0 | 0 | 1 | 0 | 0 | 0 |
| **New.CleanUp.ReferenceOTU221913** | 1 | 1 | 0 | 0 | 0 | 1 | 0 | 0 | 0 | 0 | 0 | 0 |
| **New.CleanUp.ReferenceOTU242062** | 0 | 1 | 0 | 0 | 0 | 0 | 0 | 0 | 1 | 1 | 0 | 0 |
| **New.CleanUp.ReferenceOTU251110** | 0 | 0 | 0 | 0 | 1 | 2 | 0 | 0 | 0 | 0 | 0 | 0 |
| **GU559753.1.1379** | 1 | 0 | 0 | 0 | 0 | 0 | 0 | 1 | 0 | 0 | 0 | 0 |
| **New.ReferenceOTU552** | 0 | 0 | 0 | 0 | 0 | 2 | 0 | 0 | 0 | 0 | 0 | 0 |
| **New.ReferenceOTU1200** | 0 | 0 | 0 | 0 | 0 | 0 | 0 | 0 | 1 | 1 | 0 | 0 |
| **New.CleanUp.ReferenceOTU535** | 2 | 0 | 0 | 0 | 0 | 0 | 0 | 0 | 0 | 0 | 0 | 0 |
| **New.CleanUp.ReferenceOTU6587** | 0 | 2 | 0 | 0 | 0 | 0 | 0 | 0 | 0 | 0 | 0 | 0 |
| **New.CleanUp.ReferenceOTU6912** | 0 | 2 | 0 | 0 | 0 | 0 | 0 | 0 | 0 | 0 | 0 | 0 |
| **New.CleanUp.ReferenceOTU7070** | 1 | 1 | 0 | 0 | 0 | 0 | 0 | 0 | 0 | 0 | 0 | 0 |
| **New.CleanUp.ReferenceOTU7081** | 0 | 0 | 0 | 0 | 0 | 0 | 0 | 0 | 0 | 2 | 0 | 0 |
| **New.CleanUp.ReferenceOTU7097** | 0 | 0 | 0 | 0 | 0 | 0 | 0 | 0 | 0 | 2 | 0 | 0 |
| **New.CleanUp.ReferenceOTU9535** | 0 | 0 | 0 | 0 | 0 | 0 | 0 | 0 | 1 | 0 | 0 | 1 |
| **New.CleanUp.ReferenceOTU11285** | 0 | 2 | 0 | 0 | 0 | 0 | 0 | 0 | 0 | 0 | 0 | 0 |
| **New.CleanUp.ReferenceOTU13025** | 0 | 0 | 0 | 0 | 0 | 0 | 0 | 0 | 0 | 0 | 2 | 0 |
| **New.CleanUp.ReferenceOTU18204** | 1 | 0 | 0 | 0 | 0 | 0 | 0 | 0 | 1 | 0 | 0 | 0 |
| **New.CleanUp.ReferenceOTU19160** | 2 | 0 | 0 | 0 | 0 | 0 | 0 | 0 | 0 | 0 | 0 | 0 |
| **New.CleanUp.ReferenceOTU19832** | 0 | 0 | 1 | 0 | 0 | 0 | 0 | 1 | 0 | 0 | 0 | 0 |
| **New.CleanUp.ReferenceOTU23849** | 0 | 0 | 0 | 0 | 0 | 0 | 0 | 1 | 1 | 0 | 0 | 0 |
| **New.CleanUp.ReferenceOTU26124** | 0 | 0 | 0 | 0 | 0 | 0 | 0 | 0 | 0 | 1 | 0 | 1 |
| **New.CleanUp.ReferenceOTU27774** | 0 | 0 | 0 | 0 | 0 | 0 | 0 | 0 | 0 | 2 | 0 | 0 |
| **New.CleanUp.ReferenceOTU32574** | 1 | 1 | 0 | 0 | 0 | 0 | 0 | 0 | 0 | 0 | 0 | 0 |
| **New.CleanUp.ReferenceOTU33712** | 0 | 0 | 0 | 0 | 0 | 0 | 0 | 0 | 0 | 2 | 0 | 0 |
| **New.CleanUp.ReferenceOTU36937** | 0 | 1 | 0 | 0 | 0 | 0 | 0 | 1 | 0 | 0 | 0 | 0 |
| **New.CleanUp.ReferenceOTU42501** | 0 | 0 | 0 | 0 | 1 | 1 | 0 | 0 | 0 | 0 | 0 | 0 |
| **New.CleanUp.ReferenceOTU43351** | 0 | 2 | 0 | 0 | 0 | 0 | 0 | 0 | 0 | 0 | 0 | 0 |
| **New.CleanUp.ReferenceOTU44731** | 0 | 0 | 0 | 0 | 0 | 1 | 0 | 1 | 0 | 0 | 0 | 0 |
| **New.CleanUp.ReferenceOTU47977** | 0 | 0 | 0 | 0 | 0 | 0 | 0 | 0 | 0 | 2 | 0 | 0 |
| **New.CleanUp.ReferenceOTU48988** | 0 | 0 | 0 | 2 | 0 | 0 | 0 | 0 | 0 | 0 | 0 | 0 |
| **New.CleanUp.ReferenceOTU53130** | 0 | 0 | 0 | 0 | 0 | 0 | 0 | 2 | 0 | 0 | 0 | 0 |
| **New.CleanUp.ReferenceOTU54673** | 0 | 1 | 0 | 0 | 0 | 0 | 0 | 0 | 1 | 0 | 0 | 0 |
| **New.CleanUp.ReferenceOTU55100** | 2 | 0 | 0 | 0 | 0 | 0 | 0 | 0 | 0 | 0 | 0 | 0 |
| **New.CleanUp.ReferenceOTU55346** | 1 | 0 | 0 | 0 | 0 | 0 | 0 | 0 | 1 | 0 | 0 | 0 |
| **New.CleanUp.ReferenceOTU56677** | 0 | 0 | 0 | 0 | 0 | 0 | 0 | 0 | 0 | 0 | 2 | 0 |
| **New.CleanUp.ReferenceOTU62470** | 0 | 1 | 0 | 0 | 0 | 0 | 0 | 0 | 0 | 1 | 0 | 0 |
| **New.CleanUp.ReferenceOTU66173** | 0 | 0 | 0 | 0 | 0 | 0 | 0 | 1 | 0 | 0 | 1 | 0 |
| **New.CleanUp.ReferenceOTU68581** | 0 | 0 | 0 | 0 | 0 | 2 | 0 | 0 | 0 | 0 | 0 | 0 |
| **New.CleanUp.ReferenceOTU70472** | 0 | 1 | 0 | 0 | 0 | 0 | 0 | 0 | 1 | 0 | 0 | 0 |
| **New.CleanUp.ReferenceOTU70660** | 0 | 1 | 0 | 0 | 0 | 0 | 0 | 0 | 1 | 0 | 0 | 0 |
| **New.CleanUp.ReferenceOTU74972** | 0 | 0 | 0 | 0 | 0 | 0 | 0 | 0 | 1 | 1 | 0 | 0 |
| **New.CleanUp.ReferenceOTU82903** | 0 | 0 | 0 | 0 | 0 | 0 | 0 | 0 | 0 | 0 | 0 | 2 |
| **New.CleanUp.ReferenceOTU87344** | 0 | 1 | 0 | 0 | 0 | 0 | 0 | 1 | 0 | 0 | 0 | 0 |
| **New.CleanUp.ReferenceOTU87817** | 1 | 0 | 0 | 0 | 0 | 0 | 0 | 0 | 0 | 0 | 1 | 0 |
| **New.CleanUp.ReferenceOTU89207** | 0 | 1 | 0 | 1 | 0 | 0 | 0 | 0 | 0 | 0 | 0 | 0 |
| **New.CleanUp.ReferenceOTU91441** | 0 | 0 | 0 | 0 | 0 | 0 | 0 | 0 | 0 | 2 | 0 | 0 |
| **New.CleanUp.ReferenceOTU92201** | 0 | 2 | 0 | 0 | 0 | 0 | 0 | 0 | 0 | 0 | 0 | 0 |
| **New.CleanUp.ReferenceOTU93047** | 0 | 0 | 0 | 0 | 2 | 0 | 0 | 0 | 0 | 0 | 0 | 0 |
| **New.CleanUp.ReferenceOTU93661** | 0 | 2 | 0 | 0 | 0 | 0 | 0 | 0 | 0 | 0 | 0 | 0 |
| **New.CleanUp.ReferenceOTU100231** | 0 | 1 | 0 | 0 | 0 | 1 | 0 | 0 | 0 | 0 | 0 | 0 |
| **New.CleanUp.ReferenceOTU101383** | 0 | 0 | 0 | 0 | 0 | 0 | 0 | 2 | 0 | 0 | 0 | 0 |
| **New.CleanUp.ReferenceOTU107581** | 0 | 0 | 0 | 0 | 0 | 0 | 0 | 0 | 0 | 2 | 0 | 0 |
| **New.CleanUp.ReferenceOTU107852** | 0 | 0 | 0 | 0 | 0 | 0 | 0 | 0 | 1 | 0 | 1 | 0 |
| **New.CleanUp.ReferenceOTU107927** | 0 | 0 | 0 | 0 | 1 | 1 | 0 | 0 | 0 | 0 | 0 | 0 |
| **New.CleanUp.ReferenceOTU110695** | 1 | 0 | 0 | 0 | 0 | 1 | 0 | 0 | 0 | 0 | 0 | 0 |
| **New.CleanUp.ReferenceOTU111750** | 0 | 0 | 0 | 0 | 0 | 0 | 0 | 2 | 0 | 0 | 0 | 0 |
| **New.CleanUp.ReferenceOTU111954** | 1 | 1 | 0 | 0 | 0 | 0 | 0 | 0 | 0 | 0 | 0 | 0 |
| **New.CleanUp.ReferenceOTU112179** | 0 | 0 | 0 | 1 | 0 | 0 | 0 | 0 | 0 | 0 | 0 | 1 |
| **New.CleanUp.ReferenceOTU112638** | 0 | 0 | 2 | 0 | 0 | 0 | 0 | 0 | 0 | 0 | 0 | 0 |
| **New.CleanUp.ReferenceOTU115880** | 0 | 1 | 0 | 0 | 0 | 0 | 0 | 0 | 0 | 0 | 1 | 0 |
| **New.CleanUp.ReferenceOTU116599** | 0 | 1 | 0 | 0 | 0 | 0 | 0 | 0 | 0 | 0 | 1 | 0 |
| **New.CleanUp.ReferenceOTU117528** | 1 | 0 | 0 | 0 | 0 | 0 | 0 | 0 | 0 | 0 | 0 | 1 |
| **New.CleanUp.ReferenceOTU118243** | 0 | 0 | 0 | 0 | 0 | 0 | 0 | 0 | 0 | 1 | 0 | 1 |
| **New.CleanUp.ReferenceOTU119293** | 0 | 0 | 0 | 0 | 0 | 0 | 0 | 0 | 2 | 0 | 0 | 0 |
| **New.CleanUp.ReferenceOTU120381** | 0 | 0 | 0 | 0 | 0 | 0 | 0 | 2 | 0 | 0 | 0 | 0 |
| **New.CleanUp.ReferenceOTU123054** | 0 | 0 | 0 | 0 | 1 | 1 | 0 | 0 | 0 | 0 | 0 | 0 |
| **New.CleanUp.ReferenceOTU124089** | 2 | 0 | 0 | 0 | 0 | 0 | 0 | 0 | 0 | 0 | 0 | 0 |
| **New.CleanUp.ReferenceOTU126562** | 0 | 0 | 0 | 0 | 0 | 0 | 0 | 0 | 1 | 1 | 0 | 0 |
| **New.CleanUp.ReferenceOTU127717** | 0 | 0 | 0 | 0 | 0 | 0 | 0 | 2 | 0 | 0 | 0 | 0 |
| **New.CleanUp.ReferenceOTU128643** | 0 | 0 | 0 | 0 | 1 | 1 | 0 | 0 | 0 | 0 | 0 | 0 |
| **New.CleanUp.ReferenceOTU129685** | 0 | 0 | 0 | 0 | 0 | 0 | 0 | 0 | 0 | 2 | 0 | 0 |
| **New.CleanUp.ReferenceOTU131326** | 2 | 0 | 0 | 0 | 0 | 0 | 0 | 0 | 0 | 0 | 0 | 0 |
| **New.CleanUp.ReferenceOTU133380** | 0 | 0 | 0 | 0 | 0 | 0 | 0 | 0 | 1 | 0 | 1 | 0 |
| **New.CleanUp.ReferenceOTU133735** | 0 | 0 | 0 | 0 | 0 | 0 | 0 | 0 | 0 | 1 | 0 | 1 |
| **New.CleanUp.ReferenceOTU136941** | 2 | 0 | 0 | 0 | 0 | 0 | 0 | 0 | 0 | 0 | 0 | 0 |
| **New.CleanUp.ReferenceOTU138844** | 0 | 1 | 0 | 0 | 0 | 0 | 0 | 1 | 0 | 0 | 0 | 0 |
| **New.CleanUp.ReferenceOTU139495** | 0 | 0 | 0 | 0 | 1 | 0 | 0 | 1 | 0 | 0 | 0 | 0 |
| **New.CleanUp.ReferenceOTU140934** | 1 | 0 | 0 | 0 | 0 | 0 | 0 | 0 | 1 | 0 | 0 | 0 |
| **New.CleanUp.ReferenceOTU143292** | 0 | 0 | 0 | 0 | 0 | 0 | 0 | 2 | 0 | 0 | 0 | 0 |
| **New.CleanUp.ReferenceOTU145068** | 0 | 0 | 0 | 0 | 0 | 0 | 0 | 1 | 1 | 0 | 0 | 0 |
| **New.CleanUp.ReferenceOTU145139** | 0 | 0 | 0 | 0 | 0 | 0 | 0 | 0 | 0 | 1 | 1 | 0 |
| **New.CleanUp.ReferenceOTU147169** | 1 | 0 | 0 | 0 | 0 | 0 | 0 | 0 | 0 | 1 | 0 | 0 |
| **New.CleanUp.ReferenceOTU151232** | 0 | 2 | 0 | 0 | 0 | 0 | 0 | 0 | 0 | 0 | 0 | 0 |
| **New.CleanUp.ReferenceOTU154285** | 2 | 0 | 0 | 0 | 0 | 0 | 0 | 0 | 0 | 0 | 0 | 0 |
| **New.CleanUp.ReferenceOTU160554** | 0 | 2 | 0 | 0 | 0 | 0 | 0 | 0 | 0 | 0 | 0 | 0 |
| **New.CleanUp.ReferenceOTU164943** | 0 | 0 | 0 | 0 | 0 | 1 | 0 | 0 | 1 | 0 | 0 | 0 |
| **New.CleanUp.ReferenceOTU172242** | 1 | 0 | 0 | 0 | 1 | 0 | 0 | 0 | 0 | 0 | 0 | 0 |
| **New.CleanUp.ReferenceOTU172586** | 0 | 0 | 0 | 0 | 0 | 0 | 0 | 2 | 0 | 0 | 0 | 0 |
| **New.CleanUp.ReferenceOTU174271** | 2 | 0 | 0 | 0 | 0 | 0 | 0 | 0 | 0 | 0 | 0 | 0 |
| **New.CleanUp.ReferenceOTU178851** | 1 | 0 | 0 | 0 | 0 | 0 | 0 | 0 | 0 | 0 | 0 | 1 |
| **New.CleanUp.ReferenceOTU179333** | 0 | 2 | 0 | 0 | 0 | 0 | 0 | 0 | 0 | 0 | 0 | 0 |
| **New.CleanUp.ReferenceOTU185948** | 0 | 0 | 0 | 0 | 0 | 0 | 0 | 0 | 0 | 2 | 0 | 0 |
| **New.CleanUp.ReferenceOTU186061** | 1 | 0 | 0 | 0 | 0 | 0 | 0 | 0 | 0 | 0 | 0 | 1 |
| **New.CleanUp.ReferenceOTU189342** | 0 | 0 | 0 | 0 | 0 | 0 | 0 | 0 | 0 | 2 | 0 | 0 |
| **New.CleanUp.ReferenceOTU192564** | 0 | 2 | 0 | 0 | 0 | 0 | 0 | 0 | 0 | 0 | 0 | 0 |
| **New.CleanUp.ReferenceOTU192990** | 0 | 1 | 0 | 0 | 1 | 0 | 0 | 0 | 0 | 0 | 0 | 0 |
| **New.CleanUp.ReferenceOTU193057** | 2 | 0 | 0 | 0 | 0 | 0 | 0 | 0 | 0 | 0 | 0 | 0 |
| **New.CleanUp.ReferenceOTU194661** | 0 | 0 | 0 | 0 | 0 | 1 | 0 | 0 | 0 | 1 | 0 | 0 |
| **New.CleanUp.ReferenceOTU196401** | 0 | 0 | 0 | 0 | 0 | 2 | 0 | 0 | 0 | 0 | 0 | 0 |
| **New.CleanUp.ReferenceOTU196560** | 0 | 0 | 0 | 0 | 0 | 0 | 0 | 2 | 0 | 0 | 0 | 0 |
| **New.CleanUp.ReferenceOTU196706** | 1 | 0 | 0 | 0 | 0 | 0 | 0 | 1 | 0 | 0 | 0 | 0 |
| **New.CleanUp.ReferenceOTU198564** | 0 | 0 | 0 | 0 | 0 | 0 | 0 | 1 | 0 | 1 | 0 | 0 |
| **New.CleanUp.ReferenceOTU199394** | 0 | 0 | 1 | 1 | 0 | 0 | 0 | 0 | 0 | 0 | 0 | 0 |
| **New.CleanUp.ReferenceOTU202715** | 0 | 0 | 0 | 0 | 2 | 0 | 0 | 0 | 0 | 0 | 0 | 0 |
| **New.CleanUp.ReferenceOTU209225** | 0 | 0 | 0 | 0 | 0 | 0 | 0 | 0 | 1 | 0 | 0 | 1 |
| **New.CleanUp.ReferenceOTU210469** | 0 | 0 | 0 | 0 | 0 | 0 | 0 | 1 | 0 | 1 | 0 | 0 |
| **New.CleanUp.ReferenceOTU212282** | 1 | 0 | 0 | 0 | 0 | 0 | 0 | 0 | 0 | 1 | 0 | 0 |
| **New.CleanUp.ReferenceOTU214989** | 0 | 0 | 0 | 0 | 0 | 0 | 0 | 0 | 0 | 0 | 0 | 2 |
| **New.CleanUp.ReferenceOTU215646** | 0 | 0 | 0 | 0 | 0 | 0 | 0 | 0 | 0 | 2 | 0 | 0 |
| **New.CleanUp.ReferenceOTU216843** | 0 | 0 | 0 | 0 | 0 | 0 | 0 | 0 | 2 | 0 | 0 | 0 |
| **New.CleanUp.ReferenceOTU217120** | 1 | 1 | 0 | 0 | 0 | 0 | 0 | 0 | 0 | 0 | 0 | 0 |
| **New.CleanUp.ReferenceOTU217379** | 1 | 1 | 0 | 0 | 0 | 0 | 0 | 0 | 0 | 0 | 0 | 0 |
| **New.CleanUp.ReferenceOTU219313** | 0 | 0 | 0 | 0 | 0 | 0 | 0 | 0 | 2 | 0 | 0 | 0 |
| **New.CleanUp.ReferenceOTU220475** | 0 | 0 | 0 | 0 | 0 | 0 | 0 | 0 | 2 | 0 | 0 | 0 |
| **New.CleanUp.ReferenceOTU221955** | 0 | 0 | 0 | 0 | 0 | 1 | 0 | 0 | 0 | 0 | 1 | 0 |
| **New.CleanUp.ReferenceOTU222676** | 1 | 0 | 0 | 0 | 0 | 0 | 0 | 0 | 1 | 0 | 0 | 0 |
| **New.CleanUp.ReferenceOTU226779** | 0 | 0 | 0 | 0 | 0 | 0 | 0 | 0 | 0 | 1 | 1 | 0 |
| **New.CleanUp.ReferenceOTU226886** | 0 | 1 | 0 | 0 | 0 | 0 | 0 | 0 | 0 | 0 | 1 | 0 |
| **New.CleanUp.ReferenceOTU228184** | 0 | 1 | 0 | 0 | 0 | 0 | 0 | 0 | 1 | 0 | 0 | 0 |
| **New.CleanUp.ReferenceOTU228628** | 1 | 0 | 0 | 0 | 0 | 0 | 0 | 0 | 0 | 1 | 0 | 0 |
| **New.CleanUp.ReferenceOTU249505** | 2 | 0 | 0 | 0 | 0 | 0 | 0 | 0 | 0 | 0 | 0 | 0 |
| **New.CleanUp.ReferenceOTU251874** | 0 | 1 | 0 | 0 | 0 | 0 | 0 | 1 | 0 | 0 | 0 | 0 |
| **New.CleanUp.ReferenceOTU252040** | 0 | 0 | 0 | 0 | 0 | 0 | 0 | 2 | 0 | 0 | 0 | 0 |
| **New.CleanUp.ReferenceOTU254105** | 1 | 0 | 0 | 0 | 0 | 1 | 0 | 0 | 0 | 0 | 0 | 0 |
| **Sum of OTU counts within genus** | 125707 | 86453 | 4866 | 5396 | 27704 | 46595 | 30944 | 49631 | 80320 | 89248 | 34005 | 33518 |
| **Total OTU counts in sample** | 199344 | 145130 | 145477 | 140499 | 132846 | 221728 | 197444 | 208429 | 189896 | 194669 | 183205 | 200691 |

| ***Table S12.*** *Result of searching the NCBI 16s rDNA sequence (Baceria and Archaea) database using Megablast (done in april 2019) using the most abundant Clostridium sensu stricto OTUs.* | | | | | | | |
| --- | --- | --- | --- | --- | --- | --- | --- |
| **OTU identifyers** | **Description** | **Max score** | **Total score** | **Query cover** | **E value** | **Ident** | **Accession** |
| **AB509213.1.1431** | Clostridium luticellarii strain FW431 16S ribosomal RNA, partial sequence | 2623 | 2623 | 100% | 0.0 | 99.72% | NR_145907.1 |
| **CBXI010000035.40.1552** | Clostridium tyrobutyricum strain KCTC 5387, complete genome | 2750 | 16493 | 100% | 0.0 | 100.00% | CP014170.1 |

**AB509213.1.1431 OTU sequence:**

GACGAACGCTGGCGGCGTGCCTAACACATGCAAGTCGAGCGAAGAAGCTCCTTCGGGAGGTTCTTAGCGGCGGACGGGTG

AGTAACACGTGGGTAACCTGCCTCAAAGAGGGGGATAGCCTCCCGAAAGGGAGATTAATACCGCATAATAAGTGCAGTTC

GCATGAACCGCACTTTAAAGGAGAAATCCGCTTTGAGATGGACCCGCGGCGCATTAGCTAGTTGGTAAGGCAGCGGCTTA

CCAAGGCAACGATGCGTAGCCGACCTGAGAGGGTGAACGGCCACATTGGAACTGAGAGACGGTCCAGACTCCTACGGGAG

GCAGCAGTGGGGAATATTGCACAATGGGCGAAAGCCTGATGCAGCAACGCCGCGTGAGTGAAGAAGGTTTTCGGATTGTA

AAGCTCTGTCATCTGGGACGATAATGACGGTACCAGATGAGGAAGCCACGGCTAACTACGTGCCAGCAGCCGCGGTAATA

CGTAGGTGGCAAGCGTTGTCCGGAATTACTGGGCGTAAAGGGTGCGCAGGCGGACATTTAAGTGAGATGTGAAAGACCCG

GGCTTAACTTGGGCAGTGCATTTCAAACTGGATGTCTGGAGTGCAGGAGAGGAGAACGGAATTCCTAGTGTAGCGGTGAA

ATGCGTAGAGATTAGGAAGAACACCAGTGGCGAAGGCGGTTCTCTGGACTGTAACTGACGCTGAGGCACGAAAGCGTGGG

TAGCAAACAGGATTAGATACCCTGGTAGTCCACGCCGTAAACGATGAGTACTAGGTGTAGGAGGTATCGACNCCTTCTGT

GCCGCAGTAAACACAATAAGTACTCCGCCTGGGAAGTACGATCGCAAGATTAAAACTCAAAGGAATTGACGGGGCCCGCA

CAAGCAGCGGAGCATGTGGTTTAATTCGAAGCAACGCGAAGAACCTTACCTGGACTTGACATCCCCTGCATATCTTAGAG

ATAAGAGAAGCCCTTCGGGGCAGGGAGACAGGTGGTGCATGGTTGTCGTCAGCTCGTGTCGTGAGATGTTAGGTTAAGTC

CTGCAACGAGCGCAACCCCTATTGTTAGTTGCTAGCAGTAAGATGAGCACTCTAACGAGACAGCCGCGGTTAACGCGGAG

GAAGGTGGGGATGACGTCAAATCATCATGCCCCTTATGTCCAGGGCAACACACGTGCTACAATGGGCAGAACAGAGAGAA

GCAAGACCGCGAGGTGGAGCGAACCTTGAAAACTGCTCCCAGTTCGGATTGCAGGCTGAAACCCGCCTGCATGAAGCTGG

AGTTGCTAGTAATCGCGAATCAGCATGTCGCGGTGAATACGTTCCCGGGCCTTGTACACACCGCCCGTCACACCATGAGA

GCTGGCAACACCCGAAGTCCGTAGTCTAACGAAAGAGGACGCGGCCGAAGGTGGGGTTAGTGATTGGGGTG

**CBXI010000035.40.1552 OTU sequence:**

AGAGTTTGATCCTGGCTCAGGACGAACGCTGGCGGCGTGCCTAACACATGCAAGTCGAGCGATGAAACCCCTTCGGGGGT

GGATTAGCGGCGGACGGGTGAGTAACACGTGGGTAACCTGCCTCAAAGTGGGGGATAGCCTTCCGAAAGGAAGATTAATA

CCGCATAAAGCCAAGTTTCACATGGAATTTGGATGAAAGGAGTAATTCGCTTTGAGATGGACCCGCGGCGCATTAGTTAG

TTGGTGGGGTAATGGCCTACCAAGACAGCGATGCGTAGCCGACCTGAGAGGGTGATCGGCCACATTGGAACTGAGATACG

GTCCAGACTCCTACGGGAGGCAGCAGTGGGGAATATTGCACAATGGGCGAAAGCCTGATGCAGCAACGCCGCGTGAGTGA

TGAAGGTCTTCGGATTGTAAAGCTCTGTCTTTTGGGACGATAATGACGGTACCAAAGGAGGAAGCCACGGCTAACTACGT

GCCAGCAGCCGCGGTAATACGTAGGTGGCGAGCGTTGTCCGGATTTACTGGGCGTAAAGGGTGCGTAGGCGGATGTTTAA

GTGAGATGTGAAATACCCGGGCTTAACTTGGGTGCTGCATTTCAAACTGGATATCTAGAGTGCAGGAGAGGAGAATGGAA

TTCCTAGTGTAGCGGTGAAATGCGTAGAGATTAGGAAGAACACCAGTGGCGAAGGCGATTCTCTGGACTGTAACTGACGC

TGAGGCACGAAAGCGTGGGTAGCAAACAGGATTAGATACCCTGGTAGTCCACGCCGTAAACGATGAGTACTAGGTGTAGG

AGGTATCGACCCCTTCTGTGCCGCAGTAAACACATTAAGTACTCCGCCTGGGAAGTACGATCGCAAGATTAAAACTCAAA

GGAATTGACGGGGGCCCGCACAAGCAGCGGAGCATGTGGTTTAATTCGAAGCAACGCGAAGAACCTTACCTGGACTTGAC

ATCCCCTGAATAACCTAGAGATAGGCGAAGCCCTTCGGGGCAGGGAGACAGGTGGTGCATGGTTGTCGTCAGCTCGTGTC

GTGAGATGTTAGGTTAAGTCCTGCAACGAGCGCAACCCTTATTGTTAGTTGCTAACATTCAGTTGAGCACTCTAACGAGA

CTGCCGCGGTTAACGCGGAGGAAGGTGGGGATGACGTCAAATCATCATGCCCCTTATGTCCAGGGCAACACACGTGCTAC

AATGGGCAGAACAAAGAGAAGCAATACCGCGAGGTGGAGCCAAACTCAAAAACTGCTCTCAGTTCGGATTGCAGGCTGAA

ACTCGCCTGCATGAAGCTGGAGTTGCTAGTAATCGCGAATCAGCATGTCGCGGTGAATACGTTCCCGGGCCTTGTACACA

CCGCCCGTCACACCATGAGAGCTGGCAACACCCGAAGTCCGTAGTCTAACGTAAGAGGACGCGGCCGAAGGTGGGGTTAG

TGATTGGGGTGAAGTCGTAACAAGGTAGCCGTAGGAGAACCTGCGGCTG

| **Table S13.** The table shows the OTU counts as classified within the Candidatus Methanogranum genus. The samples were taken at the end of the steady states of phase III (day 90) (pH 5.8) and phase IV (day 125) (pH 5.5) of continuous **methanol** based **propionate** elongation in an anaerobic open-culture reactor and at the end of the steady state of phase V (day 69) (methanol in influent 250 mM) and during the last day of phase VI (day 106) (methanol in influent 400 mM) of continuous **methanol** based **propionate and acetate** elongation in an anaerobic open-culture reactor at 309 K. The inocula samples from the continuous methanol based acetate elongation reactor and the propionate elongation batch experiment with initial pH 7 (Figure 1) are shown as well. For all biomass samples the duplo results are shown indicated by 1 &2. OTUs where only one hit was found in total in all 12 samples were omitted due to the size of the table. | | | | | | | | | | | | |
| --- | --- | --- | --- | --- | --- | --- | --- | --- | --- | --- | --- | --- |
|  | **OTU counts** | | | | | | | | | | | |
|  | Inoculum: continuous methanol based acetate elongation | | Inoculum: Propionate; pH 5.8 (Figure 1) | | Propionate; **pH 5.8**; 250 mM methanol | | Propionate; **pH 5.5**; 250 mM methanol | | Propionate&acetate; pH 5.8; **250 mM methanol** | | Propionate&acetate; pH 5.8; **400 mM methanol** | |
| **OTU identifiers** | 1 | 2 | 1 | 2 | 1 | 2 | 1 | 2 | 1 | 2 | 1 | 2 |
| **JF980498.1.1419** | 12109 | 8781 | 15769 | 18348 | 38560 | 71682 | 48792 | 44685 | 33959 | 36779 | 61411 | 75857 |
| **New.CleanUp.ReferenceOTU147471** | 17 | 28 | 58 | 40 | 107 | 203 | 110 | 99 | 43 | 78 | 129 | 179 |
| **New.ReferenceOTU188** | 83 | 64 | 7 | 16 | 70 | 98 | 45 | 59 | 135 | 173 | 55 | 73 |
| **New.CleanUp.ReferenceOTU233223** | 83 | 61 | 3 | 2 | 38 | 57 | 40 | 43 | 112 | 145 | 46 | 67 |
| **New.CleanUp.ReferenceOTU119462** | 0 | 0 | 5 | 7 | 32 | 63 | 73 | 32 | 0 | 3 | 97 | 85 |
| **New.CleanUp.ReferenceOTU192467** | 18 | 7 | 0 | 4 | 28 | 41 | 16 | 29 | 33 | 41 | 29 | 55 |
| **New.CleanUp.ReferenceOTU69684** | 0 | 2 | 1 | 0 | 12 | 27 | 42 | 17 | 0 | 0 | 35 | 50 |
| **EU885156.1.1224** | 1 | 0 | 2 | 2 | 6 | 14 | 12 | 4 | 16 | 17 | 12 | 45 |
| **New.CleanUp.ReferenceOTU16757** | 6 | 0 | 5 | 3 | 5 | 22 | 17 | 21 | 14 | 3 | 7 | 18 |
| **New.CleanUp.ReferenceOTU234806** | 0 | 3 | 1 | 2 | 0 | 8 | 11 | 5 | 7 | 13 | 17 | 10 |
| **New.CleanUp.ReferenceOTU196551** | 0 | 0 | 27 | 35 | 0 | 5 | 4 | 5 | 0 | 0 | 0 | 1 |
| **New.CleanUp.ReferenceOTU134286** | 0 | 0 | 1 | 1 | 4 | 12 | 21 | 15 | 0 | 1 | 10 | 5 |
| **New.ReferenceOTU55** | 3 | 0 | 0 | 0 | 0 | 1 | 7 | 3 | 15 | 6 | 15 | 18 |
| **New.CleanUp.ReferenceOTU117169** | 0 | 0 | 20 | 26 | 0 | 1 | 5 | 4 | 1 | 1 | 5 | 2 |
| **New.CleanUp.ReferenceOTU79012** | 0 | 2 | 0 | 2 | 0 | 6 | 2 | 13 | 3 | 6 | 17 | 12 |
| **New.CleanUp.ReferenceOTU109059** | 0 | 6 | 0 | 0 | 2 | 2 | 7 | 6 | 7 | 9 | 16 | 6 |
| **AB921775.1.1444** | 4 | 0 | 0 | 6 | 4 | 9 | 10 | 1 | 5 | 1 | 12 | 8 |
| **New.CleanUp.ReferenceOTU20474** | 2 | 5 | 6 | 4 | 1 | 4 | 3 | 2 | 3 | 12 | 9 | 6 |
| **New.CleanUp.ReferenceOTU196242** | 0 | 0 | 1 | 5 | 3 | 5 | 4 | 0 | 3 | 4 | 17 | 11 |
| **New.CleanUp.ReferenceOTU152494** | 6 | 2 | 1 | 4 | 2 | 13 | 1 | 1 | 4 | 1 | 9 | 7 |
| **New.CleanUp.ReferenceOTU253003** | 2 | 0 | 0 | 0 | 4 | 2 | 2 | 6 | 6 | 2 | 7 | 5 |
| **New.CleanUp.ReferenceOTU69386** | 0 | 0 | 0 | 0 | 2 | 4 | 4 | 5 | 4 | 4 | 5 | 6 |
| **New.CleanUp.ReferenceOTU112588** | 4 | 0 | 0 | 0 | 0 | 0 | 2 | 9 | 5 | 5 | 2 | 7 |
| **New.CleanUp.ReferenceOTU207066** | 0 | 0 | 0 | 2 | 10 | 17 | 1 | 0 | 0 | 0 | 0 | 0 |
| **New.CleanUp.ReferenceOTU141379** | 0 | 0 | 9 | 4 | 4 | 1 | 5 | 2 | 0 | 0 | 2 | 0 |
| **New.CleanUp.ReferenceOTU116640** | 0 | 0 | 2 | 3 | 0 | 0 | 4 | 0 | 0 | 2 | 10 | 6 |
| **New.CleanUp.ReferenceOTU30068** | 0 | 0 | 1 | 2 | 2 | 1 | 4 | 2 | 1 | 2 | 1 | 9 |
| **New.CleanUp.ReferenceOTU171136** | 1 | 2 | 0 | 0 | 0 | 0 | 1 | 2 | 2 | 6 | 5 | 5 |
| **New.CleanUp.ReferenceOTU63323** | 0 | 0 | 0 | 0 | 0 | 0 | 2 | 6 | 2 | 1 | 1 | 10 |
| **New.CleanUp.ReferenceOTU183707** | 0 | 0 | 0 | 0 | 3 | 17 | 0 | 0 | 0 | 0 | 0 | 0 |
| **New.CleanUp.ReferenceOTU148250** | 3 | 0 | 1 | 1 | 0 | 1 | 1 | 3 | 3 | 4 | 2 | 0 |
| **New.CleanUp.ReferenceOTU151261** | 1 | 2 | 0 | 0 | 0 | 0 | 3 | 8 | 0 | 1 | 2 | 1 |
| **KP192931.1.1196** | 0 | 0 | 0 | 1 | 0 | 0 | 1 | 4 | 1 | 2 | 4 | 4 |
| **New.CleanUp.ReferenceOTU9383** | 1 | 0 | 2 | 0 | 1 | 0 | 3 | 1 | 1 | 1 | 2 | 1 |
| **New.CleanUp.ReferenceOTU186468** | 0 | 0 | 0 | 0 | 2 | 2 | 3 | 0 | 0 | 4 | 1 | 0 |
| **New.CleanUp.ReferenceOTU107265** | 0 | 0 | 2 | 3 | 0 | 0 | 0 | 0 | 3 | 3 | 0 | 1 |
| **JF807145.1.1256** | 2 | 1 | 0 | 0 | 2 | 2 | 2 | 2 | 0 | 0 | 0 | 0 |
| **New.CleanUp.ReferenceOTU123309** | 0 | 0 | 0 | 4 | 0 | 0 | 1 | 0 | 4 | 2 | 0 | 0 |
| **New.CleanUp.ReferenceOTU126884** | 0 | 0 | 2 | 1 | 4 | 4 | 0 | 0 | 0 | 0 | 0 | 0 |
| **New.CleanUp.ReferenceOTU133176** | 0 | 0 | 3 | 5 | 0 | 1 | 0 | 0 | 0 | 0 | 0 | 1 |
| **New.CleanUp.ReferenceOTU225997** | 0 | 3 | 0 | 0 | 0 | 0 | 2 | 0 | 1 | 3 | 0 | 0 |
| **New.CleanUp.ReferenceOTU7405** | 0 | 0 | 4 | 5 | 0 | 0 | 0 | 0 | 0 | 0 | 0 | 0 |
| **New.CleanUp.ReferenceOTU97403** | 0 | 0 | 0 | 0 | 0 | 4 | 0 | 0 | 3 | 0 | 0 | 2 |
| **New.CleanUp.ReferenceOTU240184** | 2 | 0 | 1 | 1 | 0 | 0 | 0 | 4 | 0 | 0 | 0 | 1 |
| **New.CleanUp.ReferenceOTU107334** | 0 | 0 | 0 | 0 | 3 | 3 | 2 | 0 | 0 | 0 | 0 | 0 |
| **New.CleanUp.ReferenceOTU241897** | 0 | 0 | 0 | 0 | 1 | 1 | 2 | 0 | 0 | 0 | 2 | 2 |
| **New.CleanUp.ReferenceOTU65778** | 1 | 1 | 0 | 0 | 0 | 0 | 0 | 1 | 1 | 1 | 1 | 2 |
| **New.CleanUp.ReferenceOTU191889** | 0 | 0 | 0 | 0 | 2 | 3 | 0 | 1 | 0 | 0 | 0 | 2 |
| **New.CleanUp.ReferenceOTU42629** | 3 | 0 | 1 | 3 | 0 | 0 | 0 | 0 | 0 | 0 | 0 | 0 |
| **New.CleanUp.ReferenceOTU64142** | 0 | 0 | 0 | 0 | 4 | 1 | 0 | 0 | 0 | 0 | 2 | 0 |
| **New.CleanUp.ReferenceOTU119694** | 0 | 0 | 1 | 2 | 3 | 0 | 0 | 1 | 0 | 0 | 0 | 0 |
| **New.CleanUp.ReferenceOTU120915** | 0 | 0 | 0 | 3 | 0 | 0 | 0 | 0 | 0 | 0 | 1 | 3 |
| **New.CleanUp.ReferenceOTU195218** | 0 | 0 | 0 | 0 | 3 | 1 | 0 | 0 | 0 | 1 | 2 | 0 |
| **New.CleanUp.ReferenceOTU133806** | 0 | 0 | 0 | 0 | 0 | 0 | 4 | 0 | 0 | 0 | 2 | 0 |
| **New.CleanUp.ReferenceOTU216729** | 0 | 0 | 0 | 0 | 1 | 0 | 4 | 0 | 0 | 0 | 0 | 1 |
| **New.CleanUp.ReferenceOTU68359** | 0 | 0 | 0 | 0 | 3 | 0 | 3 | 0 | 0 | 0 | 0 | 0 |
| **New.CleanUp.ReferenceOTU10438** | 0 | 0 | 0 | 0 | 0 | 2 | 2 | 0 | 0 | 0 | 1 | 1 |
| **New.CleanUp.ReferenceOTU230831** | 0 | 0 | 0 | 0 | 0 | 0 | 2 | 0 | 1 | 0 | 2 | 1 |
| **New.ReferenceOTU832** | 0 | 1 | 3 | 0 | 0 | 1 | 0 | 0 | 0 | 0 | 1 | 0 |
| **New.CleanUp.ReferenceOTU1944** | 0 | 0 | 3 | 1 | 0 | 0 | 0 | 0 | 0 | 0 | 2 | 0 |
| **New.CleanUp.ReferenceOTU57685** | 0 | 0 | 0 | 0 | 3 | 0 | 0 | 0 | 0 | 0 | 3 | 0 |
| **New.CleanUp.ReferenceOTU170923** | 0 | 0 | 0 | 0 | 3 | 3 | 0 | 0 | 0 | 0 | 0 | 0 |
| **New.CleanUp.ReferenceOTU228565** | 0 | 1 | 0 | 0 | 0 | 1 | 2 | 1 | 0 | 0 | 0 | 0 |
| **New.CleanUp.ReferenceOTU235600** | 0 | 0 | 0 | 0 | 0 | 1 | 2 | 0 | 0 | 1 | 0 | 1 |
| **New.CleanUp.ReferenceOTU215853** | 0 | 0 | 0 | 0 | 0 | 1 | 1 | 0 | 3 | 0 | 0 | 0 |
| **New.CleanUp.ReferenceOTU3437** | 0 | 0 | 0 | 0 | 1 | 4 | 0 | 0 | 0 | 0 | 0 | 0 |
| **New.CleanUp.ReferenceOTU29315** | 0 | 0 | 0 | 2 | 2 | 1 | 0 | 0 | 0 | 0 | 0 | 0 |
| **New.CleanUp.ReferenceOTU61169** | 0 | 0 | 0 | 0 | 1 | 1 | 0 | 0 | 0 | 0 | 2 | 1 |
| **New.CleanUp.ReferenceOTU112846** | 0 | 0 | 1 | 0 | 0 | 1 | 0 | 0 | 1 | 0 | 0 | 2 |
| **New.CleanUp.ReferenceOTU149871** | 0 | 0 | 0 | 4 | 0 | 0 | 0 | 1 | 0 | 0 | 0 | 0 |
| **New.CleanUp.ReferenceOTU153697** | 0 | 0 | 0 | 0 | 0 | 2 | 0 | 3 | 0 | 0 | 0 | 0 |
| **New.CleanUp.ReferenceOTU184905** | 0 | 0 | 0 | 0 | 2 | 2 | 0 | 0 | 0 | 0 | 0 | 1 |
| **New.CleanUp.ReferenceOTU185412** | 0 | 0 | 0 | 2 | 0 | 3 | 0 | 0 | 0 | 0 | 0 | 0 |
| **New.CleanUp.ReferenceOTU239463** | 1 | 0 | 0 | 0 | 0 | 1 | 0 | 0 | 0 | 0 | 3 | 0 |
| **New.CleanUp.ReferenceOTU75057** | 0 | 0 | 0 | 0 | 0 | 0 | 2 | 1 | 0 | 1 | 0 | 0 |
| **New.CleanUp.ReferenceOTU12416** | 0 | 0 | 1 | 0 | 0 | 0 | 1 | 0 | 1 | 0 | 0 | 1 |
| **New.CleanUp.ReferenceOTU20275** | 0 | 0 | 0 | 0 | 1 | 0 | 1 | 1 | 0 | 0 | 1 | 0 |
| **New.CleanUp.ReferenceOTU71198** | 0 | 0 | 0 | 0 | 0 | 0 | 1 | 1 | 0 | 0 | 2 | 0 |
| **New.CleanUp.ReferenceOTU95996** | 0 | 0 | 0 | 0 | 0 | 0 | 1 | 1 | 0 | 0 | 0 | 2 |
| **New.CleanUp.ReferenceOTU136709** | 0 | 0 | 0 | 0 | 0 | 0 | 1 | 1 | 0 | 0 | 0 | 2 |
| **New.CleanUp.ReferenceOTU217172** | 0 | 0 | 3 | 0 | 0 | 0 | 1 | 0 | 0 | 0 | 0 | 0 |
| **New.CleanUp.ReferenceOTU12076** | 0 | 0 | 0 | 4 | 0 | 0 | 0 | 0 | 0 | 0 | 0 | 0 |
| **New.CleanUp.ReferenceOTU17814** | 0 | 0 | 0 | 0 | 1 | 0 | 0 | 2 | 1 | 0 | 0 | 0 |
| **New.CleanUp.ReferenceOTU60170** | 0 | 0 | 0 | 0 | 0 | 1 | 0 | 1 | 0 | 0 | 1 | 1 |
| **New.CleanUp.ReferenceOTU86970** | 0 | 0 | 0 | 0 | 0 | 4 | 0 | 0 | 0 | 0 | 0 | 0 |
| **New.CleanUp.ReferenceOTU123346** | 0 | 0 | 0 | 0 | 0 | 0 | 0 | 0 | 1 | 0 | 0 | 3 |
| **New.CleanUp.ReferenceOTU128689** | 0 | 0 | 0 | 0 | 0 | 2 | 0 | 0 | 1 | 0 | 0 | 1 |
| **New.CleanUp.ReferenceOTU146053** | 0 | 0 | 0 | 0 | 0 | 1 | 0 | 0 | 2 | 0 | 1 | 0 |
| **New.CleanUp.ReferenceOTU238856** | 0 | 0 | 0 | 0 | 0 | 0 | 0 | 0 | 3 | 1 | 0 | 0 |
| **New.CleanUp.ReferenceOTU116228** | 1 | 0 | 0 | 0 | 0 | 0 | 1 | 0 | 0 | 0 | 1 | 0 |
| **New.CleanUp.ReferenceOTU185857** | 0 | 1 | 0 | 0 | 0 | 0 | 1 | 0 | 0 | 0 | 1 | 0 |
| **HM038373.1.1256** | 0 | 0 | 0 | 1 | 0 | 0 | 0 | 0 | 0 | 0 | 0 | 2 |
| **JQ345702.1.914** | 0 | 0 | 0 | 0 | 0 | 0 | 0 | 0 | 0 | 3 | 0 | 0 |
| **New.CleanUp.ReferenceOTU66666** | 1 | 1 | 0 | 0 | 0 | 0 | 0 | 0 | 1 | 0 | 0 | 0 |
| **New.CleanUp.ReferenceOTU74737** | 0 | 0 | 1 | 0 | 1 | 0 | 0 | 0 | 0 | 0 | 0 | 1 |
| **New.CleanUp.ReferenceOTU83061** | 0 | 0 | 0 | 0 | 2 | 1 | 0 | 0 | 0 | 0 | 0 | 0 |
| **New.CleanUp.ReferenceOTU94146** | 0 | 0 | 2 | 0 | 0 | 0 | 0 | 0 | 1 | 0 | 0 | 0 |
| **New.CleanUp.ReferenceOTU107918** | 0 | 0 | 0 | 0 | 0 | 3 | 0 | 0 | 0 | 0 | 0 | 0 |
| **New.CleanUp.ReferenceOTU113018** | 0 | 0 | 0 | 0 | 3 | 0 | 0 | 0 | 0 | 0 | 0 | 0 |
| **New.CleanUp.ReferenceOTU115769** | 0 | 0 | 0 | 0 | 0 | 0 | 0 | 0 | 0 | 0 | 1 | 2 |
| **New.CleanUp.ReferenceOTU133786** | 2 | 0 | 0 | 0 | 0 | 0 | 0 | 1 | 0 | 0 | 0 | 0 |
| **New.CleanUp.ReferenceOTU136076** | 0 | 0 | 0 | 0 | 0 | 0 | 0 | 0 | 0 | 0 | 3 | 0 |
| **New.CleanUp.ReferenceOTU150770** | 0 | 0 | 0 | 0 | 0 | 0 | 0 | 0 | 1 | 0 | 0 | 2 |
| **New.CleanUp.ReferenceOTU153077** | 0 | 0 | 0 | 0 | 0 | 0 | 0 | 0 | 0 | 2 | 0 | 1 |
| **New.CleanUp.ReferenceOTU161645** | 0 | 0 | 0 | 0 | 3 | 0 | 0 | 0 | 0 | 0 | 0 | 0 |
| **New.CleanUp.ReferenceOTU172862** | 0 | 0 | 0 | 0 | 3 | 0 | 0 | 0 | 0 | 0 | 0 | 0 |
| **New.CleanUp.ReferenceOTU183142** | 0 | 0 | 1 | 2 | 0 | 0 | 0 | 0 | 0 | 0 | 0 | 0 |
| **New.CleanUp.ReferenceOTU189995** | 0 | 1 | 0 | 0 | 1 | 1 | 0 | 0 | 0 | 0 | 0 | 0 |
| **New.CleanUp.ReferenceOTU201381** | 1 | 1 | 0 | 0 | 0 | 0 | 0 | 0 | 1 | 0 | 0 | 0 |
| **New.CleanUp.ReferenceOTU202034** | 0 | 0 | 0 | 0 | 0 | 0 | 0 | 0 | 0 | 0 | 0 | 3 |
| **New.CleanUp.ReferenceOTU207556** | 0 | 0 | 0 | 0 | 0 | 1 | 0 | 0 | 0 | 0 | 0 | 2 |
| **New.CleanUp.ReferenceOTU38810** | 0 | 0 | 0 | 0 | 0 | 0 | 2 | 0 | 0 | 0 | 0 | 0 |
| **New.CleanUp.ReferenceOTU84877** | 0 | 0 | 0 | 0 | 0 | 0 | 2 | 0 | 0 | 0 | 0 | 0 |
| **New.CleanUp.ReferenceOTU17446** | 0 | 0 | 0 | 0 | 0 | 1 | 1 | 0 | 0 | 0 | 0 | 0 |
| **New.CleanUp.ReferenceOTU55695** | 0 | 0 | 0 | 0 | 0 | 0 | 1 | 0 | 0 | 0 | 0 | 1 |
| **New.CleanUp.ReferenceOTU71685** | 0 | 0 | 0 | 0 | 0 | 0 | 1 | 0 | 0 | 0 | 1 | 0 |
| **New.CleanUp.ReferenceOTU91089** | 0 | 0 | 0 | 0 | 1 | 0 | 1 | 0 | 0 | 0 | 0 | 0 |
| **New.CleanUp.ReferenceOTU155607** | 0 | 0 | 0 | 0 | 0 | 0 | 1 | 1 | 0 | 0 | 0 | 0 |
| **New.CleanUp.ReferenceOTU178363** | 0 | 0 | 0 | 0 | 1 | 0 | 1 | 0 | 0 | 0 | 0 | 0 |
| **New.CleanUp.ReferenceOTU239760** | 0 | 0 | 0 | 0 | 0 | 0 | 1 | 1 | 0 | 0 | 0 | 0 |
| **GQ339877.1.1256** | 0 | 0 | 0 | 1 | 0 | 0 | 0 | 0 | 0 | 0 | 1 | 0 |
| **New.CleanUp.ReferenceOTU1466** | 0 | 0 | 0 | 0 | 0 | 0 | 0 | 0 | 2 | 0 | 0 | 0 |
| **New.CleanUp.ReferenceOTU17592** | 0 | 0 | 0 | 0 | 0 | 0 | 0 | 0 | 0 | 2 | 0 | 0 |
| **New.CleanUp.ReferenceOTU19941** | 0 | 0 | 0 | 0 | 0 | 0 | 0 | 0 | 0 | 1 | 1 | 0 |
| **New.CleanUp.ReferenceOTU23235** | 0 | 0 | 0 | 0 | 0 | 1 | 0 | 0 | 0 | 0 | 0 | 1 |
| **New.CleanUp.ReferenceOTU36040** | 1 | 0 | 0 | 0 | 0 | 0 | 0 | 0 | 0 | 1 | 0 | 0 |
| **New.CleanUp.ReferenceOTU40819** | 0 | 0 | 0 | 0 | 0 | 0 | 0 | 0 | 0 | 2 | 0 | 0 |
| **New.CleanUp.ReferenceOTU40925** | 0 | 0 | 0 | 0 | 0 | 0 | 0 | 0 | 0 | 0 | 1 | 1 |
| **New.CleanUp.ReferenceOTU45218** | 0 | 0 | 0 | 2 | 0 | 0 | 0 | 0 | 0 | 0 | 0 | 0 |
| **New.CleanUp.ReferenceOTU54748** | 0 | 0 | 0 | 0 | 0 | 2 | 0 | 0 | 0 | 0 | 0 | 0 |
| **New.CleanUp.ReferenceOTU69687** | 0 | 0 | 0 | 0 | 0 | 0 | 0 | 0 | 2 | 0 | 0 | 0 |
| **New.CleanUp.ReferenceOTU100980** | 0 | 0 | 0 | 0 | 0 | 0 | 0 | 0 | 0 | 2 | 0 | 0 |
| **New.CleanUp.ReferenceOTU104077** | 0 | 0 | 1 | 0 | 0 | 1 | 0 | 0 | 0 | 0 | 0 | 0 |
| **New.CleanUp.ReferenceOTU105269** | 0 | 0 | 0 | 1 | 0 | 0 | 0 | 0 | 1 | 0 | 0 | 0 |
| **New.CleanUp.ReferenceOTU106545** | 0 | 0 | 0 | 0 | 0 | 1 | 0 | 0 | 0 | 0 | 0 | 1 |
| **New.CleanUp.ReferenceOTU115998** | 0 | 0 | 0 | 0 | 0 | 1 | 0 | 0 | 0 | 0 | 1 | 0 |
| **New.CleanUp.ReferenceOTU121015** | 0 | 0 | 0 | 0 | 0 | 1 | 0 | 0 | 1 | 0 | 0 | 0 |
| **New.CleanUp.ReferenceOTU134390** | 0 | 0 | 0 | 0 | 0 | 0 | 0 | 0 | 0 | 0 | 2 | 0 |
| **New.CleanUp.ReferenceOTU135175** | 0 | 0 | 0 | 0 | 1 | 0 | 0 | 0 | 0 | 0 | 0 | 1 |
| **New.CleanUp.ReferenceOTU135205** | 0 | 0 | 0 | 0 | 0 | 0 | 0 | 0 | 0 | 0 | 0 | 2 |
| **New.CleanUp.ReferenceOTU140874** | 0 | 0 | 0 | 0 | 0 | 0 | 0 | 0 | 1 | 0 | 1 | 0 |
| **New.CleanUp.ReferenceOTU144492** | 0 | 0 | 0 | 0 | 0 | 0 | 0 | 0 | 0 | 0 | 2 | 0 |
| **New.CleanUp.ReferenceOTU154226** | 0 | 0 | 0 | 0 | 0 | 2 | 0 | 0 | 0 | 0 | 0 | 0 |
| **New.CleanUp.ReferenceOTU173599** | 0 | 0 | 0 | 0 | 0 | 0 | 0 | 0 | 2 | 0 | 0 | 0 |
| **New.CleanUp.ReferenceOTU180044** | 0 | 0 | 0 | 0 | 0 | 0 | 0 | 0 | 0 | 0 | 0 | 2 |
| **New.CleanUp.ReferenceOTU180337** | 0 | 0 | 0 | 0 | 0 | 0 | 0 | 0 | 0 | 1 | 1 | 0 |
| **New.CleanUp.ReferenceOTU180359** | 0 | 0 | 0 | 0 | 0 | 0 | 0 | 1 | 0 | 1 | 0 | 0 |
| **New.CleanUp.ReferenceOTU186160** | 0 | 0 | 0 | 0 | 0 | 0 | 0 | 0 | 0 | 0 | 0 | 2 |
| **New.CleanUp.ReferenceOTU192522** | 0 | 0 | 0 | 0 | 2 | 0 | 0 | 0 | 0 | 0 | 0 | 0 |
| **New.CleanUp.ReferenceOTU193611** | 0 | 0 | 0 | 0 | 0 | 1 | 0 | 0 | 0 | 0 | 1 | 0 |
| **New.CleanUp.ReferenceOTU196473** | 0 | 0 | 2 | 0 | 0 | 0 | 0 | 0 | 0 | 0 | 0 | 0 |
| **New.CleanUp.ReferenceOTU198411** | 0 | 1 | 0 | 0 | 0 | 0 | 0 | 0 | 1 | 0 | 0 | 0 |
| **New.CleanUp.ReferenceOTU198909** | 0 | 0 | 0 | 0 | 0 | 0 | 0 | 0 | 0 | 0 | 2 | 0 |
| **New.CleanUp.ReferenceOTU200547** | 0 | 0 | 0 | 0 | 0 | 1 | 0 | 0 | 0 | 0 | 0 | 1 |
| **New.CleanUp.ReferenceOTU208320** | 0 | 0 | 0 | 0 | 0 | 2 | 0 | 0 | 0 | 0 | 0 | 0 |
| **New.CleanUp.ReferenceOTU218862** | 0 | 0 | 0 | 0 | 0 | 2 | 0 | 0 | 0 | 0 | 0 | 0 |
| **New.CleanUp.ReferenceOTU236064** | 0 | 0 | 0 | 0 | 0 | 2 | 0 | 0 | 0 | 0 | 0 | 0 |
| **New.CleanUp.ReferenceOTU241534** | 0 | 0 | 0 | 0 | 2 | 0 | 0 | 0 | 0 | 0 | 0 | 0 |
| **New.CleanUp.ReferenceOTU245076** | 0 | 0 | 0 | 0 | 1 | 1 | 0 | 0 | 0 | 0 | 0 | 0 |
| **New.CleanUp.ReferenceOTU15718** | 0 | 0 | 0 | 0 | 0 | 0 | 1 | 0 | 0 | 0 | 0 | 0 |
| **New.CleanUp.ReferenceOTU11669** | 0 | 0 | 0 | 0 | 0 | 1 | 0 | 0 | 0 | 0 | 0 | 0 |
| **New.CleanUp.ReferenceOTU17868** | 0 | 0 | 0 | 0 | 0 | 0 | 0 | 0 | 0 | 0 | 1 | 0 |
| **New.CleanUp.ReferenceOTU19503** | 0 | 0 | 0 | 0 | 0 | 0 | 0 | 0 | 0 | 0 | 0 | 1 |
| **New.CleanUp.ReferenceOTU44116** | 0 | 0 | 0 | 1 | 0 | 0 | 0 | 0 | 0 | 0 | 0 | 0 |
| **New.CleanUp.ReferenceOTU52029** | 0 | 0 | 0 | 0 | 1 | 0 | 0 | 0 | 0 | 0 | 0 | 0 |
| **New.CleanUp.ReferenceOTU70402** | 0 | 0 | 0 | 0 | 0 | 1 | 0 | 0 | 0 | 0 | 0 | 0 |
| **New.CleanUp.ReferenceOTU87427** | 0 | 0 | 0 | 0 | 1 | 0 | 0 | 0 | 0 | 0 | 0 | 0 |
| **New.CleanUp.ReferenceOTU93358** | 0 | 0 | 0 | 0 | 0 | 0 | 0 | 0 | 0 | 1 | 0 | 0 |
| **New.CleanUp.ReferenceOTU96117** | 0 | 0 | 0 | 0 | 0 | 1 | 0 | 0 | 0 | 0 | 0 | 0 |
| **New.CleanUp.ReferenceOTU146393** | 0 | 0 | 0 | 0 | 0 | 0 | 0 | 0 | 0 | 1 | 0 | 0 |
| **New.CleanUp.ReferenceOTU153478** | 0 | 0 | 0 | 0 | 0 | 0 | 0 | 1 | 0 | 0 | 0 | 0 |
| **New.CleanUp.ReferenceOTU158867** | 0 | 0 | 0 | 0 | 0 | 1 | 0 | 0 | 0 | 0 | 0 | 0 |
| **New.CleanUp.ReferenceOTU177958** | 0 | 0 | 1 | 0 | 0 | 0 | 0 | 0 | 0 | 0 | 0 | 0 |
| **New.CleanUp.ReferenceOTU189262** | 0 | 0 | 0 | 0 | 0 | 1 | 0 | 0 | 0 | 0 | 0 | 0 |
| **New.CleanUp.ReferenceOTU207068** | 0 | 0 | 0 | 0 | 0 | 0 | 0 | 0 | 0 | 0 | 0 | 1 |
| **New.CleanUp.ReferenceOTU207251** | 0 | 0 | 1 | 0 | 0 | 0 | 0 | 0 | 0 | 0 | 0 | 0 |
| **New.CleanUp.ReferenceOTU219348** | 0 | 1 | 0 | 0 | 0 | 0 | 0 | 0 | 0 | 0 | 0 | 0 |
| **New.CleanUp.ReferenceOTU219577** | 0 | 1 | 0 | 0 | 0 | 0 | 0 | 0 | 0 | 0 | 0 | 0 |
| **New.CleanUp.ReferenceOTU232250** | 0 | 0 | 0 | 0 | 0 | 0 | 0 | 0 | 0 | 0 | 1 | 0 |
| **New.CleanUp.ReferenceOTU240797** | 0 | 0 | 0 | 0 | 0 | 0 | 0 | 0 | 0 | 0 | 0 | 1 |
| **Sum of OTU counts within genus** | 12359 | 8979 | 15956 | 18568 | 38965 | 72400 | 49309 | 45119 | 34425 | 37357 | 62043 | 76629 |
| **Total OTU counts in sample** | 199344 | 145130 | 145477 | 140499 | 132846 | 221728 | 197444 | 208429 | 189896 | 194669 | 183205 | 200691 |

**JF980498.1.1419**

CTGGTTGATCCTGCCGGCGGCCACCGCTATAGGAATTCGATTAAGACATGCGAGTCGAGAGTCGTAATGGACTCGGCGGA

CTGCTCAGTAACACGTGGATAACGTGCCCTTAAGTGGAGGATAATCTCGGGAAATTGAGGATAATACTCCATAGATCATG

ACACCTGGAATGAGTCATGGTTCAAAGTTCCGGCGCTTAAGGATCGGTCTGCGGCCTATCAGGTAGTAGTGGGTGTAATG

TACCTACTAGCCTATGACGGGTATGGGCCTTGAGAGAGGGAGCCCAGAGTTGGATTCTGAGACACGAATCCAGGCCCTAC

GGGGCGCAGCAGTCGCGAAAACTTCACAATGGGCGCAAGCCCGATGAGGGAACTCCTAGTGCTAGCACTTTTTKTGTTAG

CTTTTCTTCAGCGTAGATAACTGAAGGAATAAGGGCTGGGTAAGACGGGTGCCAGCCGCCGCGGTAATACCTGCAGCCCA

AGTGGTGGTCGATTTTATTGAGTCTAAAACGTTCGTAGCCGGTCTGGTAAATCCTTGGGTAAATCGGAAAGCTTAACTTT

CCGAATTCCGAGGAGACTGCCAGACTTGGGACCGGGAGAGGCTAGAGGTACTTCTGGGGTAGGGGTAAAATCCTGTAATC

CTAGAAGGACCACCGGTGGCGAAGGCGTCTAGCTAGAACGGATCCGACGGTGAGGGACGAAGCCCTGGGTCGCAAACGGG

ATTAGATACCCCGGTAGTCCAGGGTGTAAACGCTGCAGACTTGGTGTTGGAGATCCTTCGAGGGTATTCAGTGCCGGAGA

GAAGTTGTTAAGTCTGCTACTTGGGGAGTACGTCCGCAAGGATGAAACTTAAAGGAATTGGTGGGGGAGCACCGCAACGG

GAGGAGCGTGCGGTTTAATTGGATTCAACACCGGAAAACTCACCAGGGGAGACTGTTACATGAAAGCCAGGCTAATGACC

TTGCTCGATTTTCAGAGAAGTGGTGCATGGCCGTCGTCAGTTCGTACCGTAAGGCGTTCTCTTAAGTGAGATAACGAACG

AGACCCTCACTAATATTTGCTACTCCGTTCTCCGGAACGGAGGCACATTATTGGGACCGCTGGCGCTAAGCCAGAGGAAG

GAGAGGTCAACGGTAGGTCAGCATGCCCTGAATCTCCTGGGCTACACGCGCGCTACAAAGGGCGGGACAATGGGTTCCGA

CACCGAAAGGTGAAGGTAATCTCGAAACCCGTCCGTAGTTCGGATTGAGGGTTGTAACTCACCCTCATGAAGCTGGATTC

CGTAGTAATCGCGAATCAACAACTCGCGGTGAATATGCCCCTGCTCCTTGCACACACCGCCCGTCAAACCATCCGAGTTG

GGTTTCAGTGAAGCTGCCTCTAACTAGGGTTGTTGAACTGAGATTTAGCAAGGAAGGTT
